# Tetracyanoethylene as a Building Block in the π-Expansion of 1,4-Dihydropyrrolo[3,2-*b*]pyrroles

**DOI:** 10.1021/acs.joc.4c01555

**Published:** 2024-10-24

**Authors:** Guler Yagiz Erdemir, Iryna Knysh, Kamil Skonieczny, Denis Jacquemin, Daniel T. Gryko

**Affiliations:** † Institute of Organic Chemistry, Polish Academy of Sciences, Kasprzaka, 01-224 Warsaw, Poland; ‡ Department of Chemistry, Faculty of Science, Gazi University, Ankara 06560, Turkey; § CNRS, CEISAM UMR 6230, Nantes Université, F-44000 Nantes, France; ∥ Institut Universitaire de France, 75005 Paris, France

## Abstract

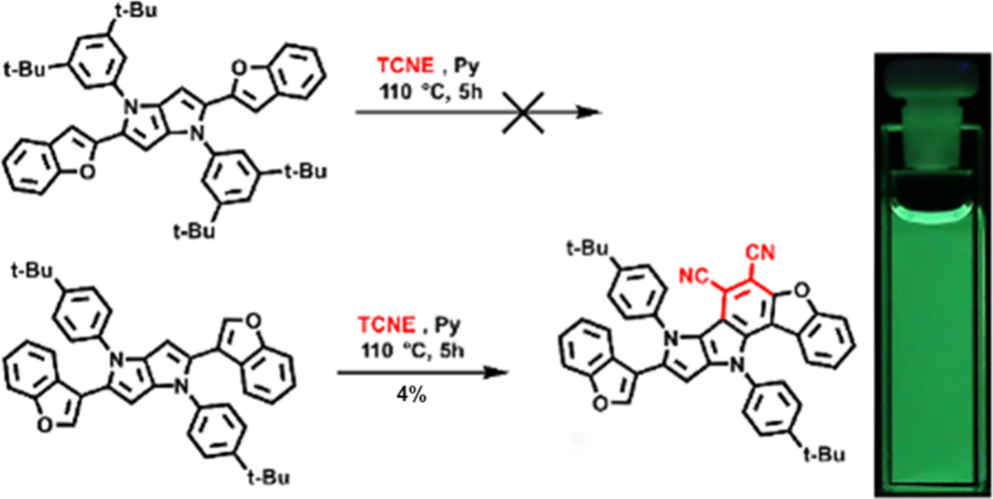

The outcome of the reaction of tetracyanoethylene with 1,4-dihydropyrrolo[3,2-*b*]pyrroles (DHPPs) strongly depends on the character of the substituents present at positions 2 and 5. With electron-withdrawing substituents, the reaction does not occur at all, while, in contrast, the presence of electron-donating substituents yields addition–elimination products. When thiazol-2-yl substituents are located at positions 2 and 5, addition occurs at the thiazole ring, rather than of the DHPP core. In cases where very electron-rich heterocycles are present at positions 2 and 5, a second addition occurs followed by aromatization, leading to the formation of an additional benzene ring bridging two heterocyclic scaffolds. The reaction occurs only at one site since the presence of the strongly electron-withdrawing tricyanoethylene group has a profound impact on electron density at the remaining free position 6. The DHPPs possessing a tricyanoethylene group are strongly polarized and thus enable a push–pull system showing red-shifted absorption and negligible fluorescence. In contrast, dyes possessing a 1,2-dicyanobenzene moiety exhibit strong emission bathochromically shifted by over 100 nm compared to parent 1,4-dihydrotetraarylpyrroles[3,2-*b*]pyrroles (TAPPs). Computational studies shed light on the evolution of the photophysical properties as a function of the substitution pattern of the final systems.

## Introduction

Push–pull (donor–acceptor) chromophores^1^ are fascinating systems that have been extensively studied for applications in various research fields, e.g., bioimaging, organic solar cell devices, optoelectronic devices, and optical data transfer.^2−5^ Their popularity stems from their appreciable combination of optical properties such as large fluorescence quantum yield, large Stokes shift, and polarity-sensitive fluorescence, which originates from their large dipole moment.^6^ The development of optoelectronic devices benefited from the fact that these strongly polarized dyes display intramolecular charge transfer (CT) and often emit in the near-infrared due to the large Stokes shifts resulting from the CT character.^7,8^ Numerous classical D-A-type chromophores such as 4-amino-1,8-naphthaleneimides,^9^ 2-amino-6-acylnaphthalenes,^10^ 7-aminocoumarins, and aminobenzo[*g*]coumarins^11^ possessing an electron-accepting group at position 3 have been extensively investigated in this regard.^12^ It is well-known that by altering the strength of electron-donating and/or electron-withdrawing groups it is possible to design dyes with improved optoelectronic properties.^3,13−16^

When considering possible donors that could be used to create these types of systems, it is worth paying attention to 1,4-dihydropyrrolo[3,2-*b*]pyrroles (DHPPs), which are highly electron-rich two-ring aromatic heterocycles, the synthesis of which has been significantly improved in the past decade.^17^ Due to the combination of superb physicochemical properties and easy availability, they have recently been used in areas such as photochromic analysis of halocarbons,^18^ organic field-effect transistors (OFETs),^19^ organic light-emitting diodes (OLEDs),^20^ resistive memory devices,^21^ bulk heterojunction organic and dye-sensitized solar cells.^22^ DHPPs are also a promising platform for the synthesis of π-expanded heteroanalogues of polycyclic aromatic hydrocarbons.^23−28^ Their ease of synthesis and susceptibility to chemical modifications make them ideal candidates for the construction of donor–acceptor systems.

Among many possible electron-withdrawing groups, the dicyanovinyl moiety is of particular importance.^29^ Less known, albeit even more powerful, is the tricyanovinyl group that typically originates from the reaction of tetracyanoethylene (TCNE) with pyrrole and other electron-rich aromatic compounds.^30−32^ The reaction involves an initial addition followed by the elimination of HCN leading to dyes possessing the general formula: Ar–C(CN)=C(CN)_2_. This almost orthogonal addition (as compared to other reaction processes) often occurs just by heating.^32−34^ The reaction’s versatility was enhanced through the introduction of pyridine, facilitating a subsequent process yielding dyes with alkyl substituents CH(CN)–CH(CN) that effectively bind and fortify the two aromatic moieties.^35,36^

The starting hypothesis in our investigation was that given the exceptional electron-rich character of 1,4-dihydropyrrolo[3,2-*b*]pyrroles^29,34^^a^, TCNE would undergo spontaneous addition at position 3, followed by a possible second addition at position 6. This should result in the formation of new D-A-type and A-D-A-type chromophores with the two tricyanovinyl moieties playing the role of an electron acceptor (A), while the DHPP core acts as an electron donor (D). It is rare to find a combination of two structural motifs with such different electronic characteristics within one molecule; therefore, very interesting spectroscopic properties can be expected from such dyes. Our preliminary results have shown that the reaction discussed above actually takes places although the substitution only occurs at one position of DHPP.^17a^ Taking into account the constantly growing interest in push–pull chromophores, we concluded that it would be worth further examining the possibilities and scope offered by this reaction as well as the properties of the obtained products. In this article, we report the results of our efforts.

## Results and Discussion

### Design and Synthesis

Research performed during the past decade has revealed that the electronic communication between the DHPP core and substituents present at positions 2 and 5 is particularly strong.^29^ In particular, the effect imparted by aryl substituents bearing electron-withdrawing groups can be considered similar to the one obtained when the electron-withdrawing groups are directly attached to the heterocyclic core. In analogy, the presence of electron-donating substituents at positions 2 and 5 markedly increases the electron density at positions 3 and 6. Since the reaction with TCNE with aromatic compounds relies on a large electron density at the aromatic ring, in order to increase the chances of success, we designed TAPPs possessing electron-rich substituents at positions 2 and 5. In particular, we resolved to use both benzene-derived substituents and five-membered heterocycles possessing one or two heteroatoms.

In contrast, substituents on the nitrogen atoms have less impact on the spectroscopic properties of DHPPs and are usually intended to control other properties of the resultant compounds such as solubility. Therefore, with the expectation that the final compounds will likely aggregate, we installed long alkyl chains as *N*-substituents to ensure good solubility.

**Scheme 1:**
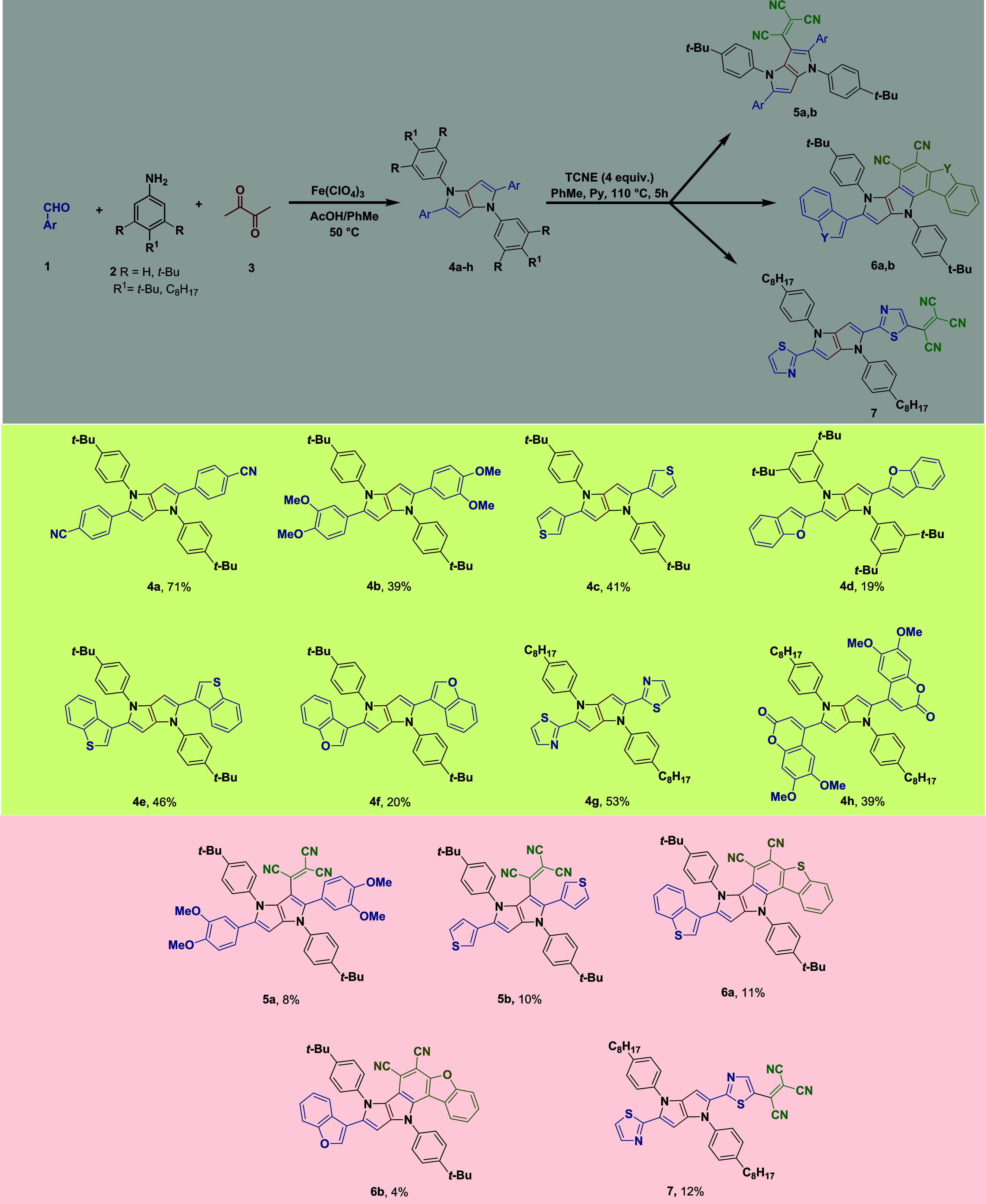
Synthesis of DHPPs **4a**–**h** and D-A-Type Chromophores **5a,b; 6a,b;** and **7**

We obtained the necessary DHPPs **4a**–**h** in good yields, starting from the appropriate aromatic aldehydes **1** and amines **2** using a multicomponent reaction catalyzed by iron perchlorate [Fe(ClO_4_)_3_·*x*H_2_O] (Scheme 1).^17a^ This small library consists primarily of DHPPs possessing electron-rich substituents at positions 2 and 5; however, compounds **4a**, **4g**, and **4h** bearing moderately electron-withdrawing substituents were also prepared, with the intention of broadly defining the scope of the final step. The key reaction, i.e., condensation with TCNE, was performed in boiling toluene in the presence of pyridine. All DHPPs were subjected to the condensation reaction, where success was met with all of those that possess electron-donating substituents, including compound **4g**-bearing thiazol-2-yl substituents. In this latter case, however, ^1^H NMR spectra pointed out a different product. The in-depth analysis of COSY, HMBC, HSQC and NOESY has revealed that an addition of TCNE occurred at positions 5 of thiazole and not at position 3 of DHPP core (see ESI for detailed analysis Figures S1–S7). This somehow surprising result can be rationalized *postfactum*, taking into consideration the following: (1) 2-dialkylaminothiazoles undergo TCNE addition at position 5;^37^ (2) steric hindrance around position 3 of DHPP core. All of the expected products were isolated in a small yield except DHPPs **4a** and **4h** bearing stronger electron-withdrawing substituents, which were unreactive under these conditions. Interestingly, compounds **4e** and **4f** having benzothiophene and benzofuran substituents, respectively, underwent further transformation, leading to products with a fused dicyanobenzene ring, apparently as a result of a second addition to the DHPP core with concomitant HCN elimination. To the best of our knowledge, this type of reactivity is unprecedented for any biaryl compounds; however, a similar outcome was observed when 2-phenylindoles were treated with DDQ (2,3-dichloro-4,5-dicyanobenzoquinone) via a Diels–Alder/oxidation/retro-Diels–Alder reaction sequence.^38^ Despite many attempts and the use of TCNE in excess, we were unable to obtain disubstituted products. Evidently, deactivation of the core of the DHPP by introducing strongly electron-withdrawing tricyanovinyl or dicyanobenzo substituents causes the reaction to stop after the first substitution.

### Photophysical Properties

**Figure 1:**
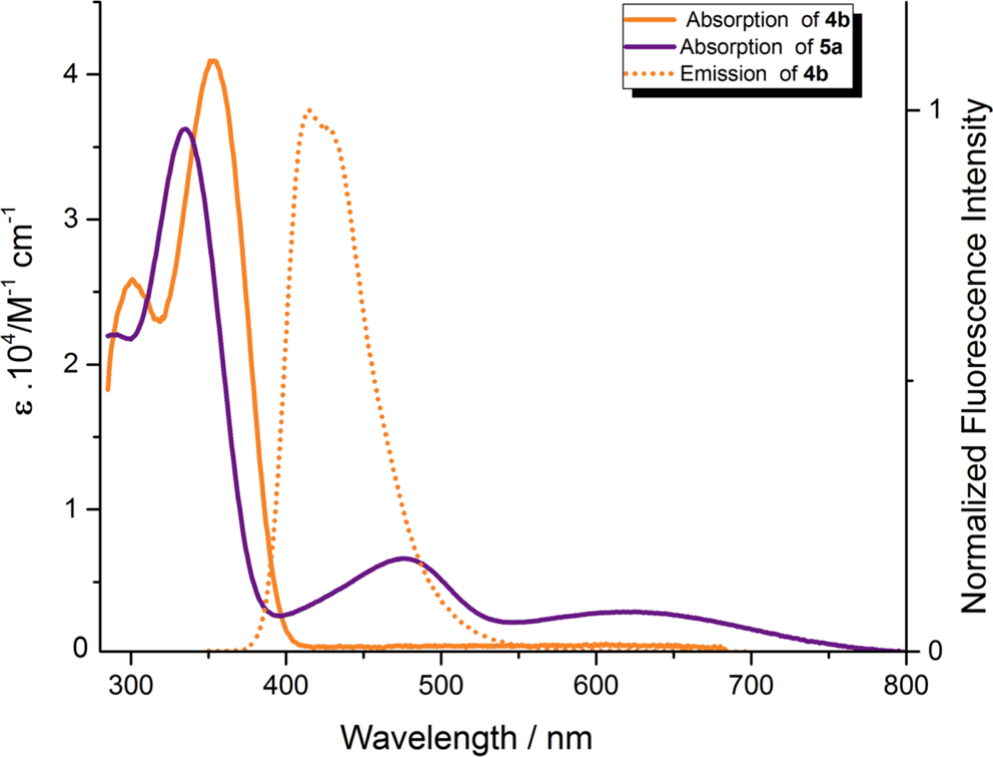
Molar extinction spectrum (solid line) and normalized fluorescence emission spectrum (short-dotted line, excitation at 340 nm of **4b**) of compounds **4b** (orange) and **5a** (purple) in toluene.

**Figure 2:**
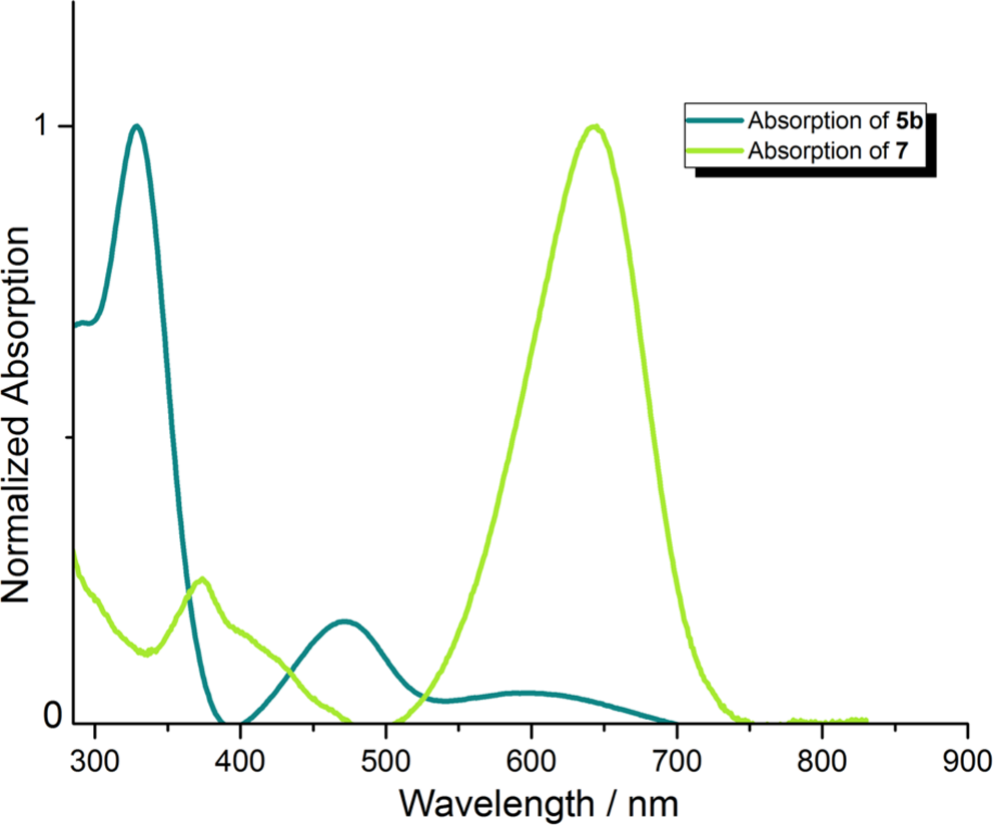
Molar extinction spectrum of compounds **5b** (blue) and **7** (green) in toluene.

**Figure 3:**
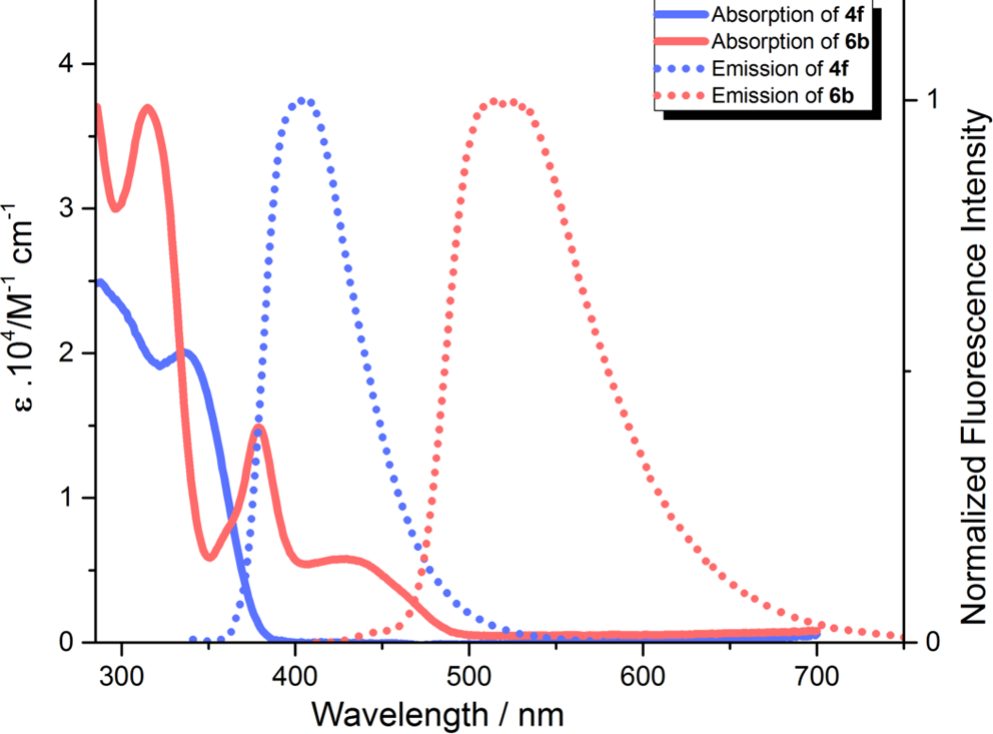
Molar extinction spectrum (solid line) and normalized fluorescence emission spectrum (short-dotted line, excitation at 330 nm of **4f** and excitation at 400 nm of **6b**) of compounds **4f** (blue) and **6b** (red) in toluene.

Having new TAPPs **4b**–**c**, **4e**-**g** in hand, we studied their photophysical properties and compared them with analogous TAPPs previously reported^17^ (Figures 1, 2 and 3, and Table 1). The photophysical features of the new TAPPs **4b**, **4c**, **4e**, **4f**, and **4g** were studied in toluene. In general, both absorption and emission are analogous to data for other TAPPs. As expected, absorption bands corresponding to intense π → π* transitions are visible, with λ_abs_^max^ located in the ultraviolet (UV) part of the electromagnetic spectra. Large changes in geometry between the ground and the excited states are responsible for red-shifted violet-blue emission (Figure 1), λ_em_^max^ were found at wavelengths ranging from 402 to 460 nm leading to Stokes shifts ranging 3000–5000 cm^–1^. Fluorescence quantum yields (Φ_fl_) of these TAPPs are in the 0.17–0.64 range (Table 1).

**Table 1:** Photophysical Properties of Derivatives **4b**, **4c**, **4e**, **4f**, **4g** in Toluene and **5a**, **5b**, **6a 6b** and **7** Obtained in Toluene, DCM and DMSO

comp.	solvent	λ_max (Abs)_ (nm)	ε@λ_max_ (M^–1^ cm^–1^)	λ_max (Em)_ (nm)	Stokes shift (cm^–1^)	Φ_fl_
**4b**	Tol	353, 300	40700, 26100	418	4400	0.64^a^
**4c**	Tol	350, 297	33300, 25400	402	3700	0.31^a^
**4e**	Tol	350, 305	23200, 17500	415	4500	0.17^a^
**4f**	Tol	335	20200	404	5100	0.22^a^
**4g**	Tol	394	42800	460	3600	0.46^b^
**5a**	Tol	630, 477, 335	3000, 6600, 36300	nd	nd	nd
	DCM	610, 482, 333	3400, 7500, 38500	nd	nd	nd
	DMSO	654, 481, 336	2900, 6000, 34800	nd	nd	nd
**5b**	Tol	590, 471, 328	2800, 6400, 33,800,	nd	nd	nd
	DCM	604, 478, 329	3600, 7000, 32000,	nd	nd	nd
	DMSO	605, 475, 330	2500, 15700	nd	nd	nd
**6a**	Tol	436, 389, 331	6400, 9200, 40500	518	3600	0.08^b^
	DCM	443, 393, 330	6800, 10,100, 46000	557	4600	0.11^b^
	DMSO	445,396, 329	2500, 3900, 17800	590	5500	0.05^b^
**6b**	Tol	429, 379, 315	5700, 1500, 37000	520	5400	0.45^b^
	DCM	440, 382, 313, 279	6100, 15100, 39300, 43400	557	4800	0.19^b^
**7**	Tol	643, 374	66700, 17200	nd	nd	nd
	DCM	660	62800	nd	nd	nd
	DMSO	639, 397	9100, 13300	nd	nd	nd

a Standard: Quinine Sulfate in H_2_SO_4_ (0.5 M Φ_fl_ = 0.54);

b Standard: Coumarin 143 in EtOH (Φ_fl_ = 0.54); nd: not detect

In contrast to parent TAPPs, the D–A-type chromophores bearing the tricyanovinylidene motif were found to have absorption bands that are markedly shifted to longer wavelengths, e.g., λ_abs_^max^ = 630 nm for **5a**. The significant red shift (about 300 nm) combined with broad bands clearly indicates the CT character of these bands, a conclusion supported by theoretical calculations (*vide infra*). In analogy to previously described push–pull chromophores bearing a tricyanovinylidene motif, dyes **5a, 5b** do not possess any measurable emission (Table 1), an outcome that was rationalized with theory. Interestingly, in the case of TAPP **7**, absorption is bathochromically shifted as far as 654 nm in toluene (Table 1 and Figure 2). The emission of this dye is below the detection limit. These effects are analogous to the previously described ones for 1,4-bis(4-octylphenyl)–2-(4-morpholinephenyl)-5-(4-nitrophenyl)-1,4-dihydropyrrolo[3,2-*b*]pyrrole which is also the DHPP-based strongly polarized donor–acceptor system.^39^

An entirely different situation occurs in the case of dyes **6a** and **6b** possessing a built-in benzene ring decorated with two cyano groups (see Figure S47a–c). Compared to TAPPs **4e**–**f**, dyes **6a** and **6b** exhibit significantly red-shifted absorption (ca. 80–90 nm in toluene) (Table 1). This can be attributed to both the expanded π-conjugation of their structures and polarization imparted by the DHPP donor moiety and 1,2-dicyanobenzene acceptor moiety. Both **6a** and **6b** exhibit positive yet limited solvatochromism with a ca. 10 nm bathochromic shift upon going from toluene to DMSO (Figure S47a), in line with a CT nature. The two factors delineated above are also responsible for a significant bathochromic shift of the emission (ca. 100 nm) compared to the precursor compounds (**4e** and **4f**, see Figure S47b,c). Importantly, the fluorescence intensity for dyes **6a** and **6b** are quite large, especially in toluene, and Φ_fl_ is significantly higher for **6b**. This is unsurprisingly accompanied by a decrease in fluorescence quantum yields due to increasing solvent polarity (Table 1). As theory reveals, a disfavorable intersystem crossing (ISC) in dye **6b** (*vide infra*) takes place; this change is likely not related to the “heavy atom” effect of sulfur.

### Computational Studies

**Figure 4:**
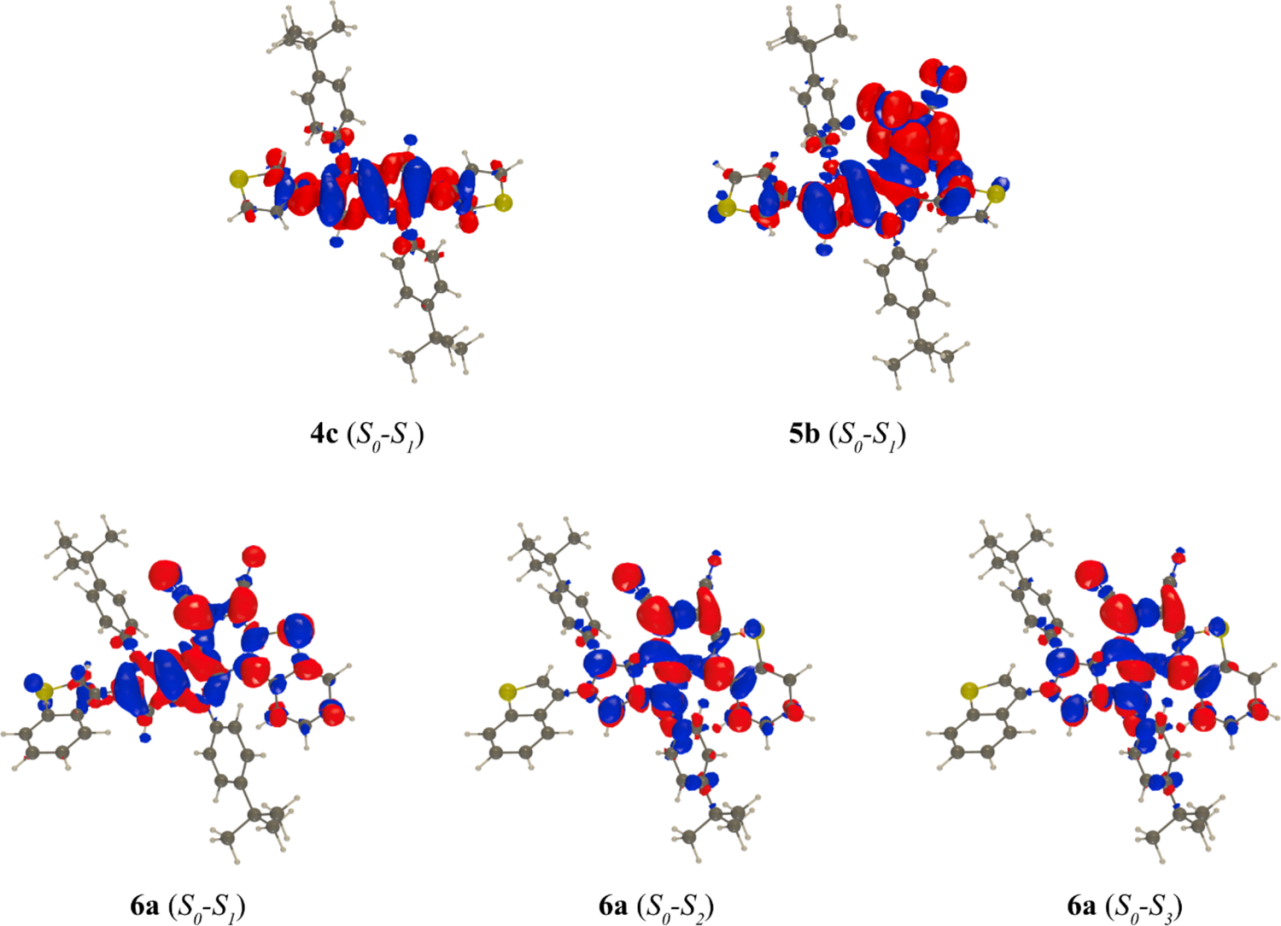
Electron density difference plot corresponding to the absorption of the selected dyes. The blue and red lobes correspond to a decrease and increase of electron density, respectively. Contour threshold 0.001 au.

To obtain complementary insights into the photophysics of the investigated compounds, we performed theoretical calculations on four relevant compounds using methods that are detailed in the SI. As expected, both TAPPs **4c** and **4e** behave like standard TAPP dyes, *i.e*., the lowest transition is bright, and it corresponds to a mild quadrupolar charge transfer (CT) from the core of the dye to the bond linking it to the side thienyl substituents. This is well illustrated by the electron density difference (EDD) plots that can be found in Figures 4 and S1 in the SI. Theory predicts 0–0 energies at 3.13 and 3.17 eV for TAPPs **4c** and **4e**, slightly red-shifted compared to the experimental values at 3.31 and 3.27 eV, respectively. As can be seen in Table 2, the relaxation of the excited state is moderate with Stokes shifts of ca. 5000–6000 cm^–1^, consistent with the measured values and the presence of a significant emission experimentally (see also below).

**Table 2:** Main Theoretical Results: The Computed Vertical Absorption and Emission Wavelength (in nm) As Well As The Computed 0-0 Energies (in eV)^a^

comp.	state	λ^vert-abs^	λ^vert-fl^		SS	Δ*E*^0–0^
**4c**	*S*_1_	328 (0.426)	399 (0.999)		5453	3.134
**4e**	*S*_1_	318 (0.401)	405 (0.939)		6779	3.171
**5b**	*S*_1_	626 (0.046)	1908(0.002)		10,745	1.103
	*S*_2_	513 (0.134)				
**6a**	*S*_1_	430 (0.066)	534 (0.049)		4543	2.299
	*S*_2_	383 (0.159)				
	*S*_3_	321 (0.915)				

a We also provide the computed Stokes shift (in cm^–1^). All of these values have been obtained using a combined CC2/TD-DFT approach (see computational details). In parentheses, we provide the CC2 oscillator strengths for both absorption and emission.

In dye **5b**, the lowest transition is much more red-shifted and corresponds to a clear CT from the electron-donating DHPP core to the strongly electron-accepting tricyanoethenyl moiety; theory predicts it at 626 nm with a low oscillator strength (Figure 4 and Table 2). The second absorption, computed at 513 nm, has a similar character but is significantly brighter. These two transitions correspond to the experimental 590 and 471 nm bands (Table 1). When optimizing the geometry of the *S*_1_ state, the electron-accepting moiety twists, becoming nearly perpendicular to the core of the dye (TICT). This leads to a structure characterized by a huge CT, of dark nature, that theory locates at 1908 nm with a trifling oscillator strength (Table 2). Consequently, the absence of a recorded emission in dye **5b** can be attributed to dark state quenching.

In heterocycle **6a**, we considered the three lowest states for absorption, and one can notice a diversity of electronic transition moving from clear CT to locally excited when going from *S*_1_ to *S*_3_ (Figure 4). The three absorptions computed at 430, 383, and 321 nm obviously correspond to the measured band maxima at 436, 389, and 331 nm, respectively. The red shifts, as compared to TAPPs **4c** and **4e**, are explained by the combined effects of the CT character and the nearly perfectly planar character of the dye core and fused benzothiophene motif. In contrast with **5b**, the fused character prevents the emergence of TICT and the relaxation of the *S*_1_ state results in a structure of similar character as for absorption, with a vertical emission computed at 534 nm and a low-yet-non-negligible oscillator strength, consistent with the measurement of a clear but weak fluorescence at 518 nm. The CT character of that emission qualitatively explains the measured solvatofluorochromism.

In an effort to obtain more quantitative estimates of the fluorescence efficiencies, we have determined the radiative and internal conversion rates using vibronic calculations^40^ and evaluated the possibility of nonradiative deactivation through intersystem crossing and the presence of low-lying minimum energy crossing points (MECPs). First, let us note that the theoretical results listed in Table S1 in the SI indicate that the spin–orbit couplings are typically small in these systems (<0.3 cm^–1^) despite the presence of sulfur atoms, except in one case (*S*_1_*-T*_3_ in **4e**) but it is an uphill process (the triplet is above the singlet). Regarding, the radiative and internal conversion rates, neglecting the presence of MECPs, the results are given in Table S1 in the SI. In summary, **5b** is nonfluorescent, due to its trifling radiative rate and large internal conversion rate, the former being reflected in very small oscillator strength (Table 2). In contrast, both **4c** and **4e** would have almost quantitative emissions due to their very large quantitative rates and logically small internal conversion ones, hinting that another deactivation pathway is at play. Finally, dye **6a** has moderate emission intensity, and the theory predicts a rather low quantum yield of 0.26 due to its low radiative rate and sizable internal conversion. We have therefore searched for the presence of MECPs, first for both **4c** and **4e** (see SI for results and details). We found that the lowest MECP corresponds to a strong twisting on one side of the molecule, as illustrated in Figures S9 and S10. As can be seen in these figures, the energy of these MECPs is higher than the ones of the excited state minimum but close to one of the FC energies, i.e., they are quite accessible. Taking into account the presence of the MECP, we compute emission quantum yields of 0.46 for **4c** and 0.16 for **4e** (Table S2), which are reasonably in line with the experimental values of 0.31 and 0.17, respectively. It is, therefore, the slightly more accessible MECP in **4e** that mainly explains its lower fluorescence efficiency.

## Conclusions

We have discovered that if exceptionally electron-rich 1,4-dihydropyrrolo[3,2-*b*]pyrroles (DHPPs) are bridged with electron-rich aromatics in such a way that the most electron-rich position is free, the reaction with tetracyanoethylene proceeds to formal insertion of the C(CN)=C(CN) moiety, effectively building an electron-deficient benzene ring between two heterocycles. In cases when aryl substituent is electron-rich but its most nucleophilic position is occupied, a single molecule of TCNE undergoes addition either to the DHPP core or to electron-rich aryl substituent, forming a strongly polarized donor–acceptor system. The presence of electron-withdrawing groups does not enable the addition to occur. The π-expansion of the dye structure via the introduction of the new aromatic ring resulted in a serious bathochromic shift of emission compared to the parent TAPP. The absence of TICT is due to the rigidity preventing strong excited-state deformation and the limited emission yield is explainable by the rather small radiative rate (strong CT) combined with a significant internal conversion rate (low-lying state). The photophysics of the products of single addition–elimination reaction is dominated by dark transitions and no fluorescence is observed due to twisted intramolecular charge transfer with the tricyanoethenyl group rotating in the excited state leading to dark state quenching.

## Experimental Section

### General Information

All reagents and solvents were purchased from commercial sources and were used as received unless otherwise noted. The reaction progress was monitored by means of thin-layer chromatography (TLC), which was performed on a Kieselgel 60. The identity of prepared compounds was proved by ^1^H NMR and ^13^C {^1^H} NMR as well as by mass spectrometry (via EI-HRMS, APCI-HRMS or ESI-HRMS). NMR spectra were measured on a Varian 500 or Varian 600 MHz instrument. Chemical shifts (δ, ppm) were determined with tetramethylsilane (TMS) as the internal reference; *J* values are given in Hz. Mass spectra were obtained with an EI ion source and an EBE double-focusing geometry mass analyzer or spectrometer equipped with an electrospray ion source with a Q-TOF type mass analyzer. Melting points were measured using an EZ-Melt automated melting point apparatus. UV–vis spectra were measured using a Shimadzu UV-3600i Plus spectrophotometer. Emission spectra were measured using an Edinburgh Instruments FS5 spectrofluorometer. The spectroscopic measurements were carried out at concentrations of 10^–6^ M to avoid aggregation and inner filter effects.

### Typical Procedure for the Synthesis of 1,4-Dihydropyrrolo[3,2-*b*]pyrroles (**4a**–**h**)

Glacial acetic acid (2 mL), toluene (2 mL), aldehyde (2 mmol, 2 equiv) and aniline (2 mmol, 2 equiv) were placed in a 50 mL round-bottom flask equipped with a magnetic stir bar. The mixture was heated at 50 °C for 1 or 2 h, depending on the aldehyde. After that time, Fe(ClO_4_)_3_*x*H_2_O (6 mol %) was added, followed by butane-2,3-dione (1 mmol, 1 equiv). The resulting mixture was stirred at 50 °C (in an oil bath) in an open flask for 16 h. The oil bath was then removed, 5 mL of acetonitrile was added to the reaction mixture and the resulting precipitate was filtered off, washed with acetonitrile (10 mL), and dried under vacuum to afford pure products **4a**–**h** as cream or yellow solids.^17a^

#### 1,4-Bis(4-(*tert*-butyl)phenyl)-2,5-bis(4-cyanophenyl)-1,4-dihydropyrrolo[3,2-*b*]pyrrole (**4a**)

Yellow solid (407 mg, 71%). Spectral and optical properties concur with literature data.^17a^

#### 1,4-Bis(4-(*tert*-butyl)phenyl)-2,5-bis(3,4-dimethoxyphenyl)-1,4-dihydropyrrolo[3,2-*b*]pyrrole (**4b**)

Cream solid (252 mg, 39%). M.p.: 247–248 °C; ^1^H NMR (500 MHz, THF-*d*_8_) δ 7.43 (d, *J* = 8.5 Hz, 4H), 7.22 (d, *J* = 8.5 Hz, 4H), 6.82 (dd, *J* = 8.3, 1.9 Hz, 2H), 6.77 (d, *J* = 8.3 Hz, 2H), 6.62 (d, *J* = 1.8 Hz, 2H), 6.29 (s, 2H), 3.74 (s, 6H), 3.47 (s, 6H), 1.35 (s, 18H); ^13^C{^1^H} NMR (126 MHz, THF-*d*_8_) δ 151.7, 150.83, 150.80, 140.8, 137.8, 133.9, 129.5, 128.3, 127.6, 122.6, 114.8, 114.3, 96.1, 57.7, 57.3, 36.8, 33.4; HRMS (APCI): *m*/*z* [M + H]^+^calcd for C_42_H_47_N_2_O_4_^+^: 643.3536; found: 643.3530.

#### 1,4-Bis(4-(*tert*-butyl)phenyl)-2,5-di(thiophen-2-yl)-1,4-dihydropyrrolo[3,2-*b*]pyrrole (**4c**)

Cream solid (220 mg, 41%). M.p.: 313 °C (dec.); ^1^H NMR (600 MHz, CDCl_3_) δ 7.39 (d, *J* = 8.5 Hz, 4H), 7.26 (d, *J* = 8.5 Hz, 4H), 7.16 (dd, *J* = 5.0, 3.0 Hz, 2H), 6.93 (dd, *J* = 5.0, 1.1 Hz, 2H), 6.80 (dd, *J* = 2.9, 1.2 Hz, 2H), 6.33 (s, 2H), 1.35 (s, 18H); ^13^C{^1^H} NMR (151 MHz, CDCl_3_) δ 149.1, 137.3, 134.4, 131.4, 131.1, 127.7, 125.9, 125.1, 124.6, 119.8, 93.5, 34.6, 31.4; HRMS (APCI): *m*/*z* [M + H]^+^ calcd for C_34_H_35_N_2_S_2_^+^: 535.2242; found: 535.2247.

#### 2,5-Bis(benzofuran-2-yl)-1,4-bis(3,5-di-*tert*-butylphenyl)-1,4-dihydropyrrolo[3,2-*b*]pyrrole (**4d**)

Yellow solid (136 mg, 19%). Spectral and optical properties concur with literature data.^17b^

#### 2,5-Bis(benzo[*b*]thiophen-3-yl)-1,4-bis(4-(*tert*-butyl)phenyl)-1,4-dihydropyrrolo[3,2-*b*]pyrrole (**4e**)

Cream solid (293 mg, 46%). M.p.: 315–316 °C; ^1^H NMR (500 MHz, CDCl_3_) δ 7.94 (d, *J* = 6.9 Hz, 2H), 7.85 (d, *J* = 7.1 Hz, 2H), 7.33 (m, 4H), 7.30 (d, *J* = 8.4 Hz, 4H), 7.22 (d, *J* = 8.3 Hz, 4H), 6.99 (s, 2H), 6.59 (s, 2H), 1.30 (s, 18H); ^13^C{^1^H} NMR (126 MHz, CDCl_3_) δ 148.5, 139.9, 138.3, 137.1, 130.5, 129.3, 129.2, 125.9, 124.6, 124.3, 124.2, 124.1, 123.6, 122.5, 95.5, 34.5, 31.3; HRMS (APCI): *m*/*z* [M + H]^+^ calcd for C_42_H_39_N_2_S_2_^+^: 635.2555; found: 635.2555.

#### 2,5-Bis(benzofuran-3-yl)-1,4-bis(4-(*tert*-butyl)phenyl)-1,4-dihydropyrrolo[3,2-*b*]pyrrole (**4f**)

Yellow solid (121 mg, 20%). M.p.: 308–310 °C; ^1^H NMR (500 MHz, CDCl_3_) δ 7.57 (d, *J* = 7.8 Hz, 2H), 7.46 (d, *J* = 8.2 Hz, 2H), 7.39 (d, *J* = 8.7 Hz, 4H), 7.35 (d, *J* = 8.5 Hz, 4H), 7.28 (t, *J* = 7.7 Hz, 2H), 7.20–7.15 (m, 4H), 6.57 (s, 2H), 1.35 (s, 18H); ^13^C{^1^H} NMR (126 MHz, CDCl_3_) δ 157.6, 152.0, 144.4, 139.7, 133.8, 129.4, 128.8, 128.5, 127.6, 127.0, 125.4, 123.7, 117.3, 114.0, 97.0, 37.2, 34.0; HRMS (APCI): *m*/*z* [M + H]^+^ calcd for C_42_H_39_N_2_O_2_^+^: 603.3012; found: 603.3015.

#### 1,4-Bis(4-octylphenyl)-2,5-bis(thiazol-2-yl)-1,4-dihydropyrrolo[3,2-*b*]pyrrole (**4g**)

Yellow solid (344 mg, 53%). M.p.: 129–130 °C; ^1^H NMR (500 MHz, CDCl_3_) δ 7.67 (d, *J* = 3.3 Hz, 2H), 7.33 (d, *J* = 8.3 Hz, 4H), 7.27 (d, *J* = 8.6 Hz, 4H), 7.07 (d, *J* = 3.3 Hz, 2H), 6.78 (s, 2H), 2.72–2.66 (m, 4H), 1.67 (q, *J* = 7.3 Hz, 4H), 1.33 (m, 20H), 0.90 (t, *J* = 6.9 Hz, 6H); ^13^C{^1^H} NMR (126 MHz, CDCl_3_) δ 159.9, 142.7, 142.4, 136.1, 133.7, 131.8, 129.3, 126.9, 117.4, 95.6, 35.6, 31.9, 31.3, 29.5, 29.3 (signal from 2 carbon atoms), 22.7, 14.1; HRMS (APCI): *m*/*z* [M + H]^+^ calcd for C_40_H_49_N_4_S_2_^+^: 649.3399; found: 649.3403.

#### 2,5-Bis(6,7-dimethoxy-2*H*-chromen-2-on-4-yl)-1,4-bis(4-octylphenyl)-1,4-dihydropyrrolo[3,2-*b*]pyrrole (**4h**)

Yellow solid (340 mg, 39%). Spectral and optical properties concur with literature data.^17a^

### Typical Procedure for the Synthesis of D-A-Type Chromophores (**5a**, **5b**, **6a**, **6b**, and **7**)

Having dissolved TAPPs (**4a**–**h**) (0.5 mmol, 1 equiv) in hot toluene (20 mL), TCNE (2 mmol, 4 equiv) and pyridine (0.5 mL) were added, respectively, in the reaction medium. The reaction mixture, which was yellowish-brown, was refluxed for 5 h at 110 °C (oil bath). Following the completion of the reaction, the solvent was removed, and column chromatography (silica, DCM/hexanes, 2:1) was used to purify any leftover residue. To obtain **5a**–**5b** or **6a**–**6b** or **7**, the eluents of the product were gathered from the column, evaporated, and then cleaned with hot MeOH.^17a^

#### 2-(1,4-Bis(4-(*tert*-butyl)phenyl)-2,5-bis(3,4-dimethoxyphenyl)-1,4-dihydropyrrolo[3,2-*b*]pyrrol-3-yl)ethene-1,1,2-tricarbonitrile (**5a**)

Black solid (29 mg, 8%). M.p.: 224–225 °C; ^1^H NMR (500 MHz, CDCl_3_) δ 7.46 (d, *J* = 8.5 Hz, 2H), 7.42 (d, *J* = 8.6 Hz, 2H), 7.20 (d, *J* = 8.5 Hz, 2H), 7.12 (d, *J* = 8.6 Hz, 2H), 6.89 (d, *J* = 8.3 Hz, 1H), 6.85 (dd, *J* = 8.3, 1.9 Hz, 1H), 6.82–6.75 (m, 2H), 6.48 (d, *J* = 1.8 Hz, 1H), 6.44 (d, *J* = 1.8 Hz, 1H), 6.33 (s, 1H), 3.91 (s, 3H), 3.87 (s, 3H), 3.51 (s, 3H), 3.49 (s, 3H), 1.38 (s, 9H), 1.34 (s, 9H); ^13^C{^1^H} NMR (126 MHz, CDCl_3_) δ 154.1, 154.0, 153.1, 151.6, 151.0, 150.8, 142.7, 141.3, 137.72, 137.66, 136.2, 134.7, 130.6, 129.1, 128.2, 128.0, 127.9, 127.8, 127.5, 124.23, 124.18, 115.8, 115.6, 114.9, 114.5, 114.2, 113.7, 113.6, 104.2, 96.0, 92.5, 58.54, 58.48, 58.3, 58.0, 34.0, 33.9; HRMS (APCI): *m*/*z* [M + H]^+^ calcd for C_47_H_46_N_5_O_4_^+^: 744.3550; found: 744.3554.

#### 2-(1,4-Bis(4-(*tert*-butyl)phenyl)-2,5-di(thiophen-3-yl)-1,4-dihydropyrrolo[3,2-*b*]pyrrol-3-yl)ethene-1,1,2-tricarbonitrile (**5b**)

Black solid (32 mg, 10%). M.p.: 240–242 °C; ^1^H NMR (500 MHz, CDCl_3_) δ 7.50 (d, *J* = 8.6 Hz, 2H), 7.43 (d, *J* = 8.6 Hz, 2H), 7.33 (dd, *J* = 5.0, 3.0 Hz, 1H), 7.22 (m, 3H), 7.22–7.17 (m, 1H), 7.17 (d, *J* = 8.7 Hz, 2H), 6.89 (dd, *J* = 2.9, 1.2 Hz, 1H), 6.84 (dd, *J* = 5.0, 1.2 Hz, 1H), 6.75 (dd, *J* = 5.0, 1.2 Hz, 1H), 6.37 (s, 1H), 1.41 (s, 9H), 1.36 (s, 9H); ^13^C{^1^H} NMR (126 MHz, CDCl_3_) δ 154.6, 154.2, 137.9, 137.5, 137.2, 136.0, 135.6, 134.6, 132.3, 131.4, 130.9, 130.6, 130.5, 129.7, 129.1, 128.4, 127.9, 127.8, 127.7, 124.9, 115.4, 114.3, 114.1, 104.6, 96.3, 92.9, 37.53, 37.45, 34.00, 33.95; HRMS (APCI): *m*/*z* [M + H]^+^ calcd for C_39_H_34_N_5_S_2_^+^: 636.2256; found: 636.2258.

#### 2-(Benzo[*b*]thiophen-3-yl)-3,11-bis(4-(*tert*-butyl)phenyl)-3,11-dihydrobenzo[4,5]thieno[2,3-*g*]pyrrolo[3,2-*b*]indole-4,5-dicarbonitrile (**6a**)

Yellow solid (38 mg, 11%). M.p.: 248–249 °C; ^1^H NMR (600 MHz, CDCl_3_) δ 7.91 (dd, *J* = 7.3, 1.5 Hz, 1H), 7.86 (d, *J* = 8.0 Hz, 1H), 7.83 (dd, *J* = 7.2, 1.5 Hz, 1H), 7.52 (d, *J* = 8.6 Hz, 2H), 7.42 (d, *J* = 8.5 Hz, 2H), 7.40–7.33 (m, 5H), 7.34 (d, *J* = 8.5 Hz, 2H), 6.92 (s, 1H), 6.89 (t, *J* = 7.3 Hz, 1H), 6.72 (d, *J* = 8.3 Hz, 1H), 6.63 (s, 1H), 1.40 (s, 9H), 1.35 (s, 9H); ^13^C{^1^H} NMR (151 MHz, CDCl_3_) δ 152.8, 151.3, 139.7, 139.4, 139.1, 138.7, 138.1, 137.6, 136.9, 136.3, 135.9, 132.5, 128.1, 127.8, 127.4, 127.2, 126.9, 126.7, 126.5, 126.2, 124.7, 124.6, 124.4, 124.2, 123.8, 123.1, 122.6, 122.3, 121.7, 117.3, 116.0, 115.1, 102.6, 102.2, 93.9, 34.9, 34.8, 31.4, 31.3; HRMS (APCI): *m*/*z* [M]^+^calcd for C_46_H_36_N_4_S_2_: 708.2381; found: 708.2387.

#### 2-(Benzofuran-3-yl)-3,11-bis(4-(*tert*-butyl)phenyl)-3,11-dihydrobenzofuro[2,3-*g*]pyrrolo[3,2-*b*]indole-4,5-dicarbonitrile (**6b**)

Yellow solid (4 mg, 4%). M.p.: 330 °C (dec.); ^1^H NMR (600 MHz, CDCl_3_) δ 7.76 (d, *J* = 7.7 Hz, 1H), 7.70 (d, *J* = 8.4 Hz, 2H), 7.63 (d, *J* = 8.2 Hz, 1H), 7.58 (d, *J* = 8.4 Hz, 2H), 7.58 (d, *J* = 8.5 Hz, 2H), 7.48 (m, 3H), 7.41 (t, *J* = 8.0 Hz, 1H), 7.33 (t, *J* = 8.0 Hz, 1H), 7.28 (d, *J* = 7.2 Hz, 1H), 6.88 (t, *J* = 7.3 Hz, 1H), 6.81 (s, 1H), 6.66 (s, 1H), 5.61 (d, *J* = 7.9 Hz, 1H), 1.52 (s, 9H), 1.43 (s, 9H); ^13^C{^1^H} NMR (126 MHz, CDCl_3_) δ 159.2, 157.2, 156.5, 155.61, 155.58, 144.9, 141.7, 139.6, 139.0, 138.8, 135.7, 131.3, 131.0, 130.3, 129.7, 129.5, 129.1, 127.6, 127.5, 125.7 (signal from 2 carbon atoms), 124.3, 123.8, 123.2, 117.8, 117.3, 116.6, 115.9, 115.8, 114.4, 114.2, 102.9, 95.1, 94.7, 37.8, 37.6, 34.1, 34.0; HRMS (APCI): *m*/*z* [M + H]^+^calcd for C_46_H_37_N_4_O_2_^+^: 677.2917; found: 677.2919.

#### 2-(2-(1,4-Bis(4-octylphenyl)-5-(thiazol-2-yl)-1,4-dihydropyrrolo[3,2-*b*]pyrrol-2-yl)thiazol-5-yl)ethene-1,1,2-tricarbonitrile (**7**)

Black-green solid (44 mg, 12%). M.p.: 220–222 °C; ^1^H NMR (600 MHz, CDCl_3_) δ 8.36 (s, 1H), 7.75 (d, *J* = 3.2 Hz, 1H), 7.40 (AA′BB′, *J* = 8.3 Hz, 2H), 7.35 (AA′BB′, *J* = 8.3 Hz, 2H), 7.32 (s, 4H), 7.20 (d, *J* = 3.2 Hz, 1H), 7.17 (d, *J* = 0.9 Hz, 1H), 6.66 (s, 1H), 2.74–2.70 (m, 4H), 1.74–1.66 (m, 4H), 1.43–1.28 (m, 20H), 0.92–0.88 (m, 6H); ^13^C{^1^H} NMR (151 MHz, CDCl_3_) δ 167.9, 158.2, 156.3, 145.8, 143.9, 143.3, 140.2, 138.6, 135.2, 135.0, 133.7, 132.4, 130.2, 129.6, 128.6, 127.82, 127.79, 127.1, 119.4, 112.5, 112.4, 100.6, 94.3, 78.9, 35.73, 35.67, 35.4, 31.90, 31.87, 31.3, 31.1, 29.5, 29.4 (signal from 2 carbon atoms), 29.28, 29.27, 29.26, 22.68, 22.67, 14.1; HRMS (APCI): *m*/*z* [M + H]^+^ calcd for C_45_H_48_N_7_S_2_^+^: 750.3413; found: 750.3402.

### Theory

See SI for theoretical methods.

## Data Availability

The data underlying this study are available in the published article and its Supporting Information.

## Supporting Information Available

Photophysical data; theoretical methods and additional data; copies of ^1^H and ^13^C{^1^H} NMR data (PDF)
