# Supplementary material for: Tetracyanoethylene as a Building Block in the π-Expansion of 1,4-Dihydropyrrolo[3,2-b]pyrroles
Source: J Org Chem. 2024 Oct 24;89(21):15513–22. doi: 10.1021/acs.joc.4c01555 (PMC11536355; doi:10.1021/acs.joc.4c01555)
Supplement: Supplementary file 1 — jo4c01555_si_001.pdf [file jo4c01555_si_001.pdf]

Tetracyanoethylene as a Building Block in the π-Expansion of 1,4-Dihydropyrrolo[3,2-*b*]pyrroles

Guler Yagiz Erdemir[a,b], Iryna Knysh[c], Kamil Skonieczny,[a] Denis Jacquemin[c,d]* and Daniel T. Gryko[a]*

[a]Institute of Organic Chemistry, Polish Academy of Sciences, Kasprzaka 01-224, Warsaw, Poland

[b]Department of Chemistry, Faculty of Science, Gazi University, Ankara 06560, Turkey

[c]Nantes Université, CNRS, CEISAM UMR 6230, F-44000 Nantes, France

[d]Institut Universitaire de France, 75005 Paris, France

Corresponding Authors

* E-mail: Denis.Jacquemin@univ-nantes.fr

* E-mail: dtgryko@icho.edu.pl

Table of Contents

1. **General Information……………………………………………………………………..S2**
2. **Synthesis and characterization data of products…………………………………………………S3**
   - **2.1 Typical procedure for the synthesis of 1,4-dihydropyrrolo[3,2-b]pyrrole (4a-h)…………….S3**
   - **2.2 Typical procedure for the synthesis of D-A-type chromophores (5a, 5b 6a, 6b and 7)………S6**
   - **2.3 Structure assignment for compound 7…………………………………………………………S10**
3. **Proposed mechanism for D−A-type chromophores ………………………………………………S16**
4. **Theoretical calculations……………………………………………………………………...S16**
   - **4.1 Methods ………………………………………………………………………………S16**
   - **4.2 Additional results ……………………………………………………………………..S19**
   - **4.3 Cartesian coordinates ...……………………………………………………………………S22**
5. **Copies of ¹H NMR, ¹³C{¹H} NMR spectra and reports of HRMS of the compounds…………...S50**
6. **Photophysical properties……………………………………………………………………S65**
7. **References ……………………………………………………………………………………S72**

1. General information

All reagents and solvents were purchased from commercial sources and were used as received unless otherwise noted. The reaction progress was monitored by means of thin-layer chromatography (TLC), which was performed on Kieselgel 60. The identity of prepared compounds was proved by ^{1}H NMR and ^{13}C NMR as well as by mass spectrometry (via EI-HRMS, APCI-HRMS or ESI-HRMS). NMR spectra were measured on Varian 500 MHz or Varian 600 MHz instrument. Chemical shifts (δ, ppm) were determined with tetramethylsilane (TMS) as the internal reference; *J* values are given in Hz. Mass spectra were obtained with EI ion source and the EBE double focusing geometry mass analyzer or spectrometer equipped with electro-spray ion source with Q-TOF type mass analyzer. Melting points were measured using EZ-Melt Automated Melting Point Apparatus. UV-vis spectra were measured using Shimadzu UV-3600i Plus spectrophotometer. Emission spectra were measured using Edinburgh Instruments FS5 spectrofluorometer. The spectroscopic measurements were carried out at the concentrations of 10^{-6} M to avoid aggregation and inner filter effects.

2. Synthesis and characterization data of products

2.1 Typical procedure for the synthesis of 1,4-dihydropyrrolo[3,2-b]pyrrole (4a-h)

Glacial acetic acid (2 mL), toluene (2 mL), aldehyde (2 mmol, 2 *eq.*) and aniline (2 mmol, 2 *eq.*) were placed in a 50 mL round-bottom flask equipped with a magnetic stir bar. The mixture was heated at 50 °C for 1 or 2 h, depending on the aldehyde. After that time, $Fe(ClO_4)_3xH_2O$ (6 mol%) was added, followed by butane-2,3-dione (1 mmol, 1 *eq.*). The resulting mixture was stirred at 50 °C (oil bath) in an open flask for 16 h. The oil bath was then removed, 5 mL of acetonitrile was added to the reaction mixture and the resulting precipitate was filtered off, washed with acetonitrile (10 mL) and dried under vacuum to afford pure products **4a-h** as cream or yellow solids.[1]

1,4-Bis(4-(*tert*-butyl)phenyl)-2,5-bis(4-cyanophenyl)-1,4-dihydropyrrolo[3,2-*b*]pyrrole (4a):

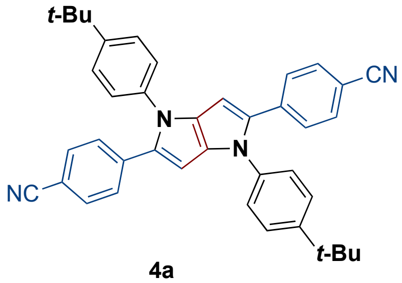

4a

Yellow solid (407 mg, 71%). Spectral and optical properties concur with literature data.[1]

1,4-Bis(4-(*tert*-butyl)phenyl)-2,5-bis(3,4-dimethoxyphenyl)-1,4-dihydropyrrolo[3,2-*b*]pyrrole (4b):

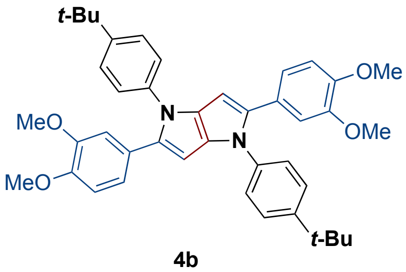

4b

Cream solid (252 mg, 39%). M.p.: 247-248 °C; ^{1}H NMR (500 MHz, THF-d_8) δ 7.43 (d, J = 8.5 Hz, 4H), 7.22 (d, J = 8.5 Hz, 4H), 6.82 (dd, J = 8.3, 1.9 Hz, 2H), 6.77 (d, J = 8.3 Hz, 2H), 6.62 (d, J = 1.8 Hz, 2H), 6.29 (s, 2H), 3.74 (s, 6H), 3.47 (s, 6H), 1.35 (s, 18H); ^{13}C{^{1}H} NMR (126 MHz, THF-d_8) δ 151.7, 150.83, 150.80, 140.8, 137.8, 133.9, 129.5, 128.3, 127.6, 122.6, 114.8, 114.3, 96.1, 57.7, 57.3, 36.8, 33.4; HRMS (APCI): *m/z* [M+H]$^+$calculated for $C_{42}H_{47}N_2O_4^+$: 643.3536; found: 643.3530.

1,4-Bis(4-(*tert*-butyl)phenyl)-2,5-di(thiophen-2-yl)-1,4-dihydropyrrolo[3,2-*b*]pyrrole (4c):

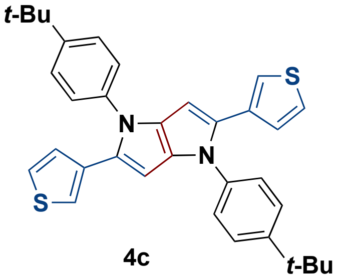

4c

Cream solid (220 mg, 41%). M.p.: 313 °C (dec.); ^{1}H NMR (600 MHz, $CDCl_3$) δ 7.39 (d, J = 8.5 Hz, 4H), 7.26 (d, J = 8.5 Hz, 4H), 7.16 (dd, J = 5.0, 3.0 Hz, 2H), 6.93 (dd, J = 5.0, 1.1 Hz, 2H), 6.80 (dd, J = 2.9, 1.2 Hz, 2H), 6.33 (s, 2H), 1.35 (s, 18H); ^{13}C{^{1}H} NMR (151 MHz, $CDCl_3$) δ 149.1, 137.3, 134.4, 131.4, 131.1, 127.7, 125.9, 125.1, 124.6, 119.8, 93.5, 34.6, 31.4; HRMS (APCI): *m/z* [M+H]$^+$calculated for $C_{34}H_{35}N_2S_2^+$: 535.2242; found: 535.2247.

2,5-Bis(benzofuran-2-yl)-1,4-bis(3,5-di-*tert*-butylphenyl)-1,4-dihydropyrrolo[3,2-*b*]pyrrole (4d):

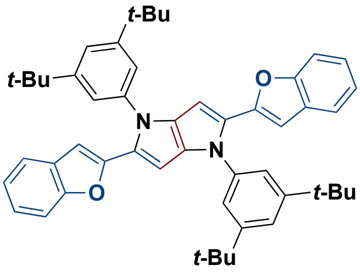

4d

Yellow solid (136 mg, 19%). Spectral and optical properties concur with literature data.[2]

2,5-Bis(benzo[*b*]thiophen-3-yl)-1,4-bis(4-(*tert*-butyl)phenyl)-1,4-dihydropyrrolo[3,2-*b*]pyrrole (4e):

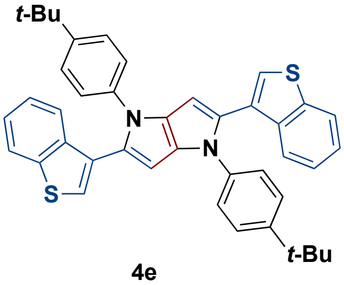

4e

Cream solid (293 mg, 46%). M.p.: 315-316 °C; ^{1}H NMR (500 MHz, CDCl$_3$) δ 7.94 (d, *J* = 6.9 Hz, 2H), 7.85 (d, *J* = 7.1 Hz, 2H), 7.33 (m, 4H), 7.30 (d, *J* = 8.4 Hz, 4H), 7.22 (d, *J* = 8.3 Hz, 4H), 6.99 (s, 2H), 6.59 (s, 2H), 1.30 (s, 18H); ^{13}C{^{1}H} NMR (126 MHz, CDCl$_3$) δ 148.5, 139.9, 138.3, 137.1, 130.5, 129.3, 129.2, 125.9, 124.6, 124.3, 124.2, 124.1, 123.6, 122.5, 95.5, 34.5, 31.3; HRMS (APCI): *m/z* [M+H]$^+$calculated for $C_{42}H_{39}N_2S_2^+$: 635.2555; found: 635.2555.

2,5-Bi(benzofuran-3-yl)-1,4-bis(4-(*tert*-butyl)phenyl)-1,4-dihydropyrrolo[3,2-*b*]pyrrole (4f):

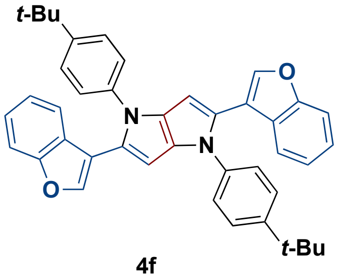

4f

Yellow solid (121 mg, 20%). M.p.: 308-310 °C; ^{1}H NMR (500 MHz, CDCl$_3$) δ 7.57 (d, *J* = 7.8 Hz, 2H), 7.46 (d, *J* = 8.2 Hz, 2H), 7.39 (d, *J* = 8.7 Hz, 4H), 7.35 (d, *J* = 8.5 Hz, 4H), 7.28 (t, *J* = 7.7 Hz, 2H), 7.20 – 7.15 (m, 4H), 6.57 (s, 2H), 1.35 (s, 18H); ^{13}C{^{1}H} NMR (126 MHz, CDCl$_3$) δ 157.6, 152.0, 144.4, 139.7, 133.8, 129.4, 128.8, 128.5, 127.6, 127.0, 125.4, 123.7, 117.3, 114.0, 97.0, 37.2, 34.0; HRMS (APCI): *m/z* [M+H]$^+$ calculated for $C_{42}H_{39}N_2O_2^+$: 603.3012; found: 603.3015.

1,4-Bis(4-octylphenyl)-2,5-di(thiazol-2-yl)-1,4-dihydropyrrolo[3,2-*b*]pyrrole-2,5-diyl)dithiazole (4g):

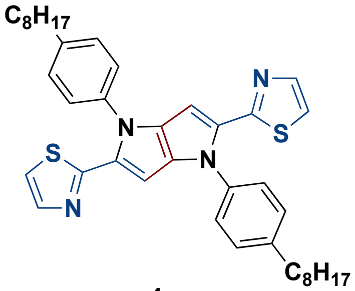

4g

Yellow solid (344 mg, 53%). M.p.: 129-130 ˚C; ^{1}H NMR (500 MHz, CDCl$_3$) δ 7.67 (d, *J* = 3.3 Hz, 2H), 7.33 (d, *J* = 8.3 Hz, 4H), 7.27 (d, *J* = 8.6 Hz, 4H), 7.07 (d, *J* = 3.3 Hz, 2H), 6.78 (s, 2H), 2.72 – 2.66 (m, 4H), 1.67 (q, *J* = 7.3 Hz, 4H), 1.33 (m, 20H), 0.90 (t, *J* = 6.9 Hz, 6H); ^{13}C{^{1}H} NMR (126 MHz, CDCl$_3$) δ 159.9, 142.7, 142.4, 136.1, 133.7, 131.8, 129.3, 126.9, 117.4, 95.6, 35.6, 31.9, 31.3, 29.5, 29.3 (signal from 2 carbon atoms), 22.7, 14.1; HRMS (APCI): *m/z* [M+H]$^+$calculated for $C_{40}H_{49}N_4S_2^+$: 649.3399; found: 649.3403.

2,5-Bis(6,7-dimethoxy-2*H*-chromen-2-on-4-yl)-1,4-bis(4-octylphenyl)-1,4-dihydropyrrolo[3,2-*b*]pyrrole (4h):

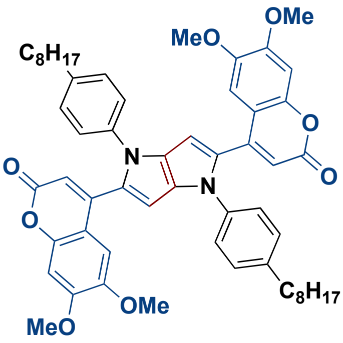

4h

Yellow solid (340 mg, 39%). Spectral and optical properties concur with literature data. [1]

2.2 Typical procedure for the synthesis of D-A-type chromophores (5a, 5b, 6a, 6b and 7)

After dissolving TAPPs (**4a-h**) (0.5 mmol, 1 *eq.*) in hot toluene (20 mL), TCNE (2 mmol, 4 *eq.*) and pyridine (0.5 mL) were added to the reaction medium. The reaction mixture, which was yellowish-brown, was refluxed for five hours at 120 °C (oil bath). Following the

completion of the reaction, the solvent was removed off and column chromatography (silica, DCM/hexanes, 1:2) was used to purify any leftover residue. To obtain **5a-5b or 6a-6b or 7** the eluents of product were gathered from column, evaporated, and then triturated with hot MeOH.[1]

2-(1,4-Bis(4-(*tert*-butyl)phenyl)-2,5-bis(3,4-dimethoxyphenyl)-1,4-dihydropyrrolo[3,2-*b*]pyrrol-3-yl)ethene-1,1,2-tricarbonitrile (5a):

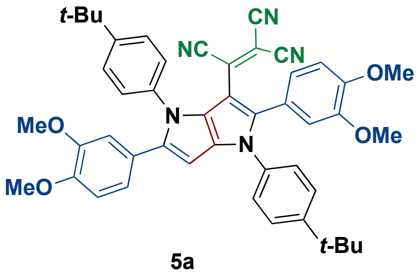

5a

Purified by column chromatography using 230-400 mesh silica gel and a mixture of dichloromethane/hexane (2:1) as an eluent. Black solid (29 mg, 8%). M.p.: 224-225 °C; ^{1}H NMR (500 MHz, $CDCl_3$) δ 7.46 (d, *J* = 8.5 Hz, 2H), 7.42 (d, *J* = 8.6 Hz, 2H), 7.20 (d, *J* = 8.5 Hz, 2H), 7.12 (d, *J* = 8.6 Hz, 2H), 6.89 (d, *J* = 8.3 Hz, 1H), 6.85 (dd, *J* = 8.3, 1.9 Hz, 1H), 6.82-6.75 (m, 2H), 6.48 (d, *J* = 1.8 Hz, 1H), 6.44 (d, *J* = 1.8 Hz, 1H), 6.33 (s, 1H), 3.91 (s, 3H), 3.87 (s, 3H), 3.51 (s, 3H), 3.49 (s, 3H), 1.38 (s, 9H), 1.34 (s, 9H); ^{13}C{^{1}H} NMR (126 MHz, $CDCl_3$) δ 154.1, 154.0, 153.1, 151.6, 151.0, 150.8, 142.7, 141.3, 137.72, 137.66, 136.2, 134.7, 130.6, 129.1, 128.2, 128.0, 127.9, 127.8, 127.5, 124.23, 124.18, 115.8, 115.6, 114.9, 114.5, 114.2, 113.7, 113.6, 104.2, 96.0, 92.5, 58.54, 58.48, 58.3, 58.0, 34.0, 33.9; HRMS (APCI): *m/z* $[M+H]^+$ calculated for $C_{47}H_{46}N_5O_4^+$: 744.3550; found: 744.3554.

2-(1,4-Bis(4-(*tert*-butyl)phenyl)-2,5-di(thiophen-3-yl)-1,4-dihydropyrrolo[3,2-*b*]pyrrol-3-yl)ethene-1,1,2-tricarbonitrile (5b):

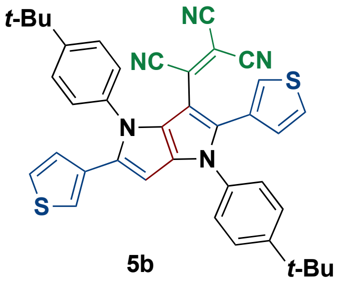

5b

Purified by column chromatography using 230-400 mesh silica gel and a mixture of dichloromethane/hexane (2:1) as an eluent. Black solid (32 mg, 10%). M.p.: 240-242 ˚C; ^{1}H NMR (500 MHz, $CDCl_3$) δ 7.50 (d, *J* = 8.6 Hz, 2H), 7.43 (d, *J* = 8.6 Hz, 2H), 7.33 (dd, *J* = 5.0, 3.0 Hz, 1H), 7.22 (m, 3H), 7.22 – 7.17 (m, 1H), 7.17 (d, *J* = 8.7 Hz, 2H), 6.89 (dd, *J* = 2.9, 1.2 Hz, 1H), 6.84 (dd, *J* = 5.0, 1.2 Hz, 1H), 6.75 (dd, *J* = 5.0, 1.2 Hz, 1H), 6.37 (s, 1H), 1.41 (s, 9H), 1.36 (s, 9H); ^{13}C{^{1}H} NMR (126 MHz, $CDCl_3$) δ 154.6, 154.2, 137.9, 137.5, 137.2, 136.0, 135.6, 134.6, 132.3, 131.4, 130.9, 130.6, 130.5, 129.7, 129.1, 128.4, 127.9, 127.8, 127.7, 124.9, 115.4, 114.3, 114.1, 104.6, 96.3, 92.9, 37.53, 37.45, 34.00, 33.95; HRMS (APCI): *m/z* $[M+H]^+$ calculated for $C_{39}H_{34}N_5S_2^+$: 636.2256; found: 636.2258.

2-(Benzo[*b*]thiophen-3-yl)-3,11-bis(4-(*tert*-butyl)phenyl)-3,11-dihydrobenzo[4,5]thieno[2,3-*g*]pyrrolo[3,2-*b*]indole-4,5-dicarbonitrile (6a):

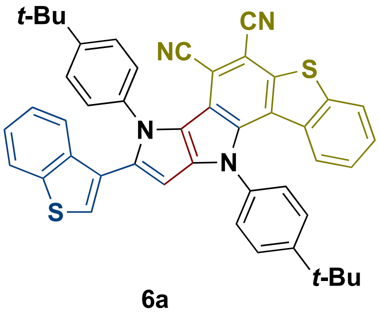

6a

Yellow solid (38 mg, 11%). M.p.: 248-249 ˚C; ^{1}H NMR (600 MHz, $CDCl_3$) δ 7.91 (dd, *J* = 7.3, 1.5 Hz, 1H), 7.86 (d, *J* = 8.0 Hz, 1H), 7.83 (dd, *J* = 7.2, 1.5 Hz, 1H), 7.52 (d, *J* = 8.6 Hz, 2H), 7.42 (d, *J* = 8.5 Hz, 2H), 7.40 – 7.33 (m, 5H), 7.34 (d, *J* = 8.5 Hz, 2H), 6.92 (s, 1H), 6.89 (t, *J* = 7.3 Hz, 1H), 6.72 (d, *J* = 8.3 Hz, 1H), 6.63 (s, 1H), 1.40 (s, 9H), 1.35 (s, 9H); ^{13}C{^{1}H} NMR (151 MHz, $CDCl_3$) δ 152.8, 151.3, 139.7, 139.4, 139.1, 138.7, 138.1, 137.6, 136.9, 136.3,

135.9, 132.5, 128.1, 127.8, 127.4, 127.2, 126.9, 126.7, 126.5, 126.2, 124.7, 124.6, 124.4, 124.2, 123.8, 123.1, 122.6, 122.3, 121.7, 117.3, 116.0, 115.1, 102.6, 102.2, 93.9, 34.9, 34.8, 31.4, 31.3; HRMS (APCI): *m/z* $[M]^+$calculated for $C_{46}H_{36}N_4S_2$: 708.2381; found: 708.2387.

2-(Benzofuran-3-yl)-3,11-bis(4-(*tert*-butyl)phenyl)-3,11-dihydrobenzofuro[2,3-*g*]pyrrolo[3,2-*b*]indole-4,5-dicarbonitrile (6b):

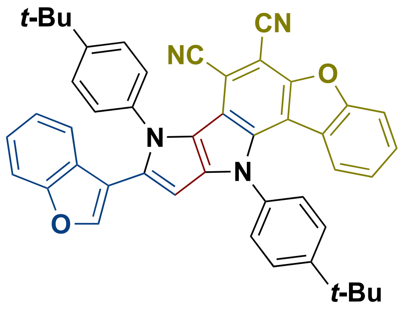

6b

Yellow solid (4 mg, 4%). M.p.: 330 °C (dec.); ^{1}H NMR (600 MHz, $CDCl_3$) δ 7.76 (d, *J* = 7.7 Hz, 1H), 7.70 (d, *J* = 8.4 Hz, 2H), 7.63 (d, *J* = 8.2 Hz, 1H), 7.58 (d, *J* = 8.4 Hz, 2H), 7.58 (d, *J* = 8.5 Hz, 2H), 7.48 (m, 3H), 7.41 (t, *J* = 8.0 Hz, 1H), 7.33 (t, *J* = 8.0 Hz, 1H), 7.28 (d, *J* = 7.2 Hz, 1H), 6.88 (t, *J* = 7.3 Hz, 1H), 6.81 (s, 1H), 6.66 (s, 1H), 5.61 (d, *J* = 7.9 Hz, 1H), 1.52 (s, 9H), 1.43 (s, 9H); $^{13}C\{^1H\}$ NMR (126 MHz, $CDCl_3$) δ 159.2, 157.2, 156.5, 155.61, 155.58, 144.9, 141.7, 139.6, 139.0, 138.8, 135.7, 131.3, 131.0, 130.3, 129.7, 129.5, 129.1, 127.6, 127.5, 125.7 (signal from 2 carbon atoms), 124.3, 123.8, 123.2, 117.8, 117.3, 116.6, 115.9, 115.8, 114.4, 114.2, 102.9, 95.1, 94.7, 37.8, 37.6, 34.1, 34.0; HRMS (APCI): *m/z* $[M+H]^+$calculated for $C_{46}H_{37}N_4O_2^+$: 677.2917; found: 677.2919.

2-(2-(1,4-Bis(4-octylphenyl)-5-(thiazol-2-yl)-1,4-dihydropyrrolo[3,2-*b*]pyrrol-2-yl)thiazol-5-yl)ethene-1,1,2-tricarbonitrile (7):

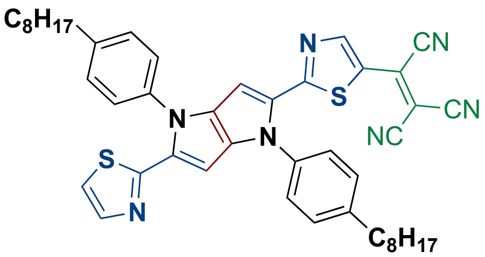

7

Purified by column chromatography using 230-400 mesh silica gel and a mixture of dichloromethane/hexane (2:1) as an eluent. Black-green solid (44 mg, 12%). M.p.: 220-222 °C; ^{1}H NMR (600 MHz, $CDCl_3$) δ 8.36 (s, 1H), 7.75 (d, J = 3.2 Hz, 1H), 7.40 (AA'BB', J = 8.3 Hz, 2H), 7.35 (AA'BB', J = 8.3 Hz, 2H), 7.32 (s, 4H), 7.20 (d, J = 3.2 Hz, 1H), 7.17 (d, J = 0.9 Hz, 1H), 6.66 (s, 1H), 2.74 – 2.70 (m, 4H), 1.74 – 1.66 (m, 4H), 1.43 – 1.28 (m, 20H), 0.92 – 0.88 (m, 6H); ^{13}C{^{1}H} NMR (151 MHz, $CDCl_3$) δ 167.9, 158.3, 156.3, 145.9, 144.0, 143.0, 140.2, 138.3, 135.3, 134.9, 133.7, 132.5, 130.3, 129.6, 128.6, 127.9, 127.8, 127.1, 119.3, 112.5, 112.4, 100.6, 94.6, 79.1, 35.73, 35.67, 31.90, 31.87, 31.3, 31.1, 29.5, 29.4 (signal from 2 carbon atoms), 29.28, 29.27, 29.26, 22.68, 22.67, 14.1; HRMS (APCI): *m/z* $[M+H]^+$ calculated for $C_{45}H_{48}N_7S_2^+$: 750.3413; found: 750.3402.

2.3. Structure assignment for compound 7

The molecular weight of **7** was in line with expectations (i.e. it confirmed the monosubstitution of the parent compound), but due to the distinct properties of compound **7** compared to other tricyanovinyl-substituted pyrrolo[3,2-*b*]pyrroles, it was suspected that in this particular case the reaction occurred on one of the side (hetero)aryl substituents. Therefore we performed an in-depth analysis of its structure using 1D and 2D NMR spectroscopy.

Based on correlations from the ^{1}H-^{1}H COSY experiment, 13 protons from the aromatic region were assigned to two sole signals at 8.36 and 7.32 ppm along with 3 spin systems [4H (AA'BB') + 2H (AB, thiazole) +2H (AB, pyrrolo[3,2-*b*]pyrrole)]. Although in the ^{1}H NMR spectrum the signal at 6.71 ppm appears to be a singlet, ^{1}H-^{1}H COSY NMR shows its weak but clearly visible correlation with the doublet at 7.17 ppm with coupling constant of 0.9 Hz. The characteristic chemical shift (usually below 7 ppm, 6.78 ppm in parent DHPP) together with such a small coupling constant (through 5 bonds, usually not detectable) indicates that the reaction did not take place on the pyrrolo[3,2-*b*]pyrrole core, but rather on one of the side

substituents. The presence of the AA'BB' spin system at 7.40 and 7.35 ppm (J = 8.3 Hz, 4H) indicates the existence of the first, *para*-substituted phenyl ring. In turn, the second *para*-substituted phenyl ring gives singlet at 7.32 ppm (4H). The above observations suggest that the tricyanvinyl substituent is located on one of the two thiazole rings. The first one is unsubstituted, as evidenced by the presence of two doublets at 7.75 and 7.20 ppm, with a coupling constant characteristic for *ortho*-oriented protons in five-membered aromatic heterocyclic rings (J = 3.2 Hz, 2H). On the other hand, the only signal from the second thiazole ring is a singlet located in more upfielded region (8.36 ppm, 1H), which is typical for 5-(tricyanovinyl)substituted thiazoles.[3]

Although it was not possible to unambiguously assign all signals to the corresponding hydrogen and carbon atoms in the ^{1}H NMR and ^{13}C NMR spectra, respectively, the performed analysis allows us to confirm the proposed structure of compound **7** with full confidence.

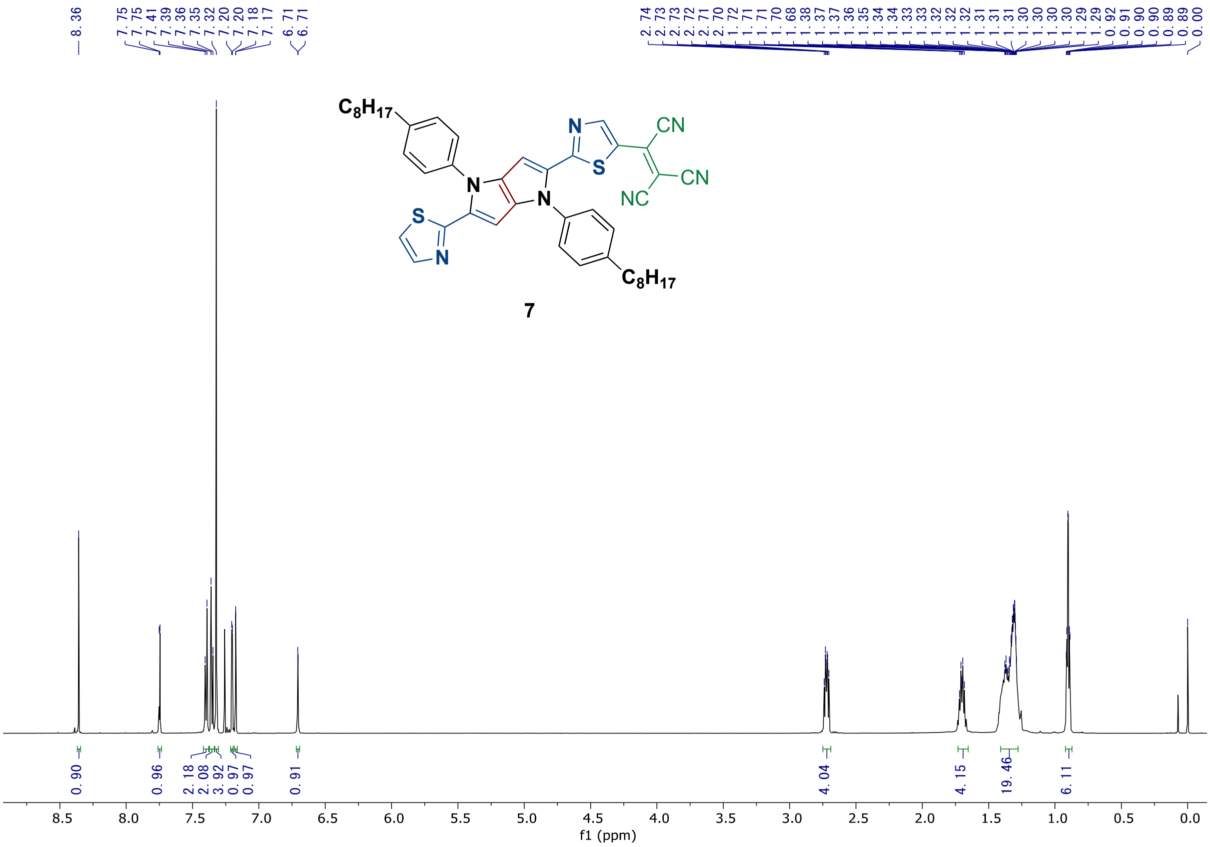

Figure S1. ^{1}H NMR (600 MHz, $CDCl_3$) spectra of compound **7**

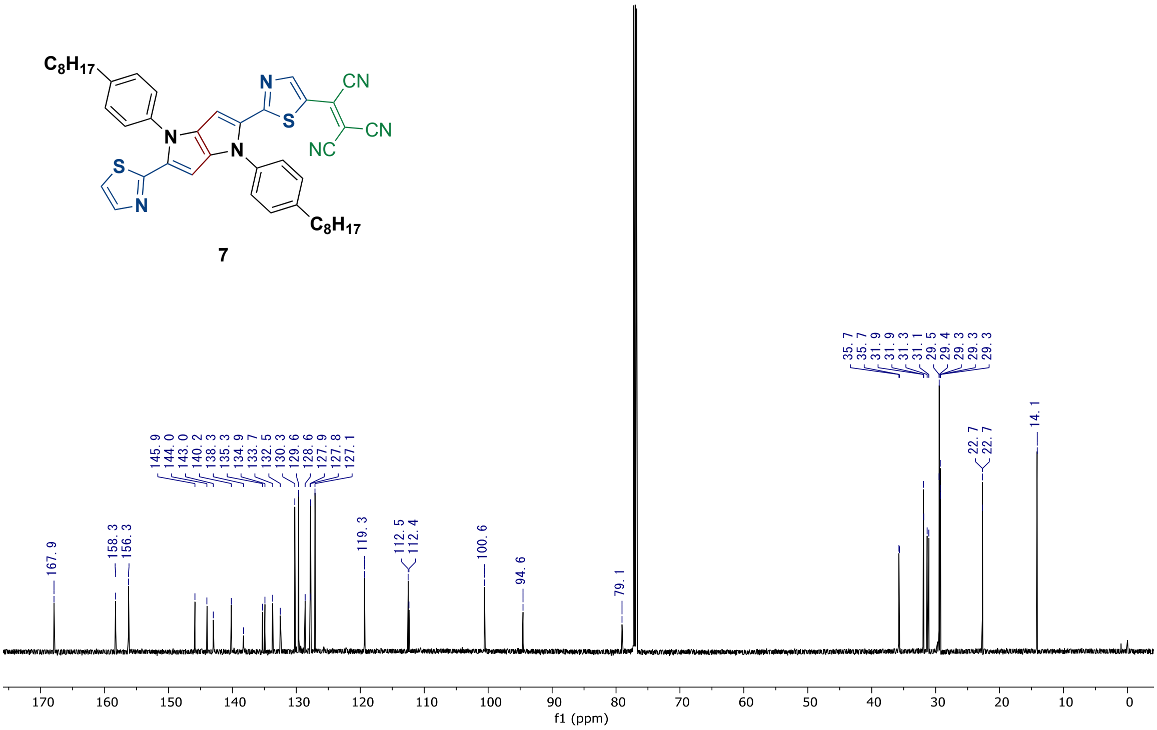

Figure S2.^{13}C NMR (600 MHz, $CDCl_3$) spectra of compound **7**

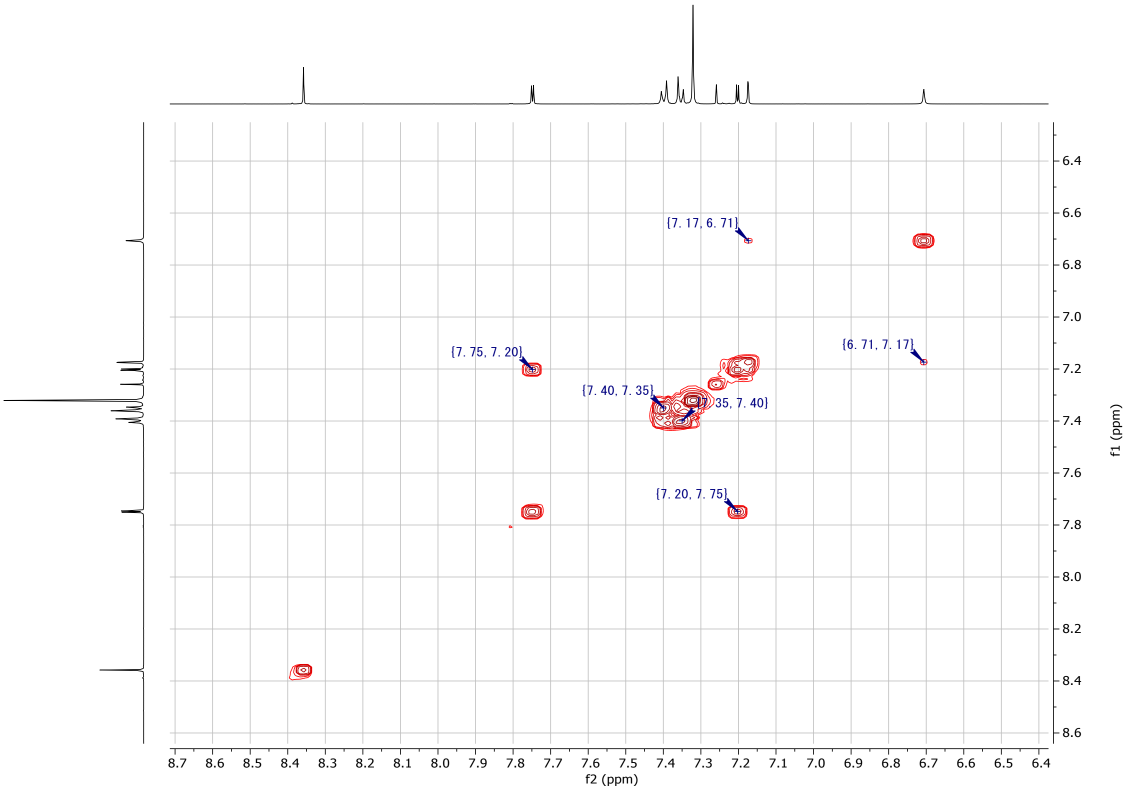

Figure S3. ^{1}H-^{1}H COSY spectra of compound **7**

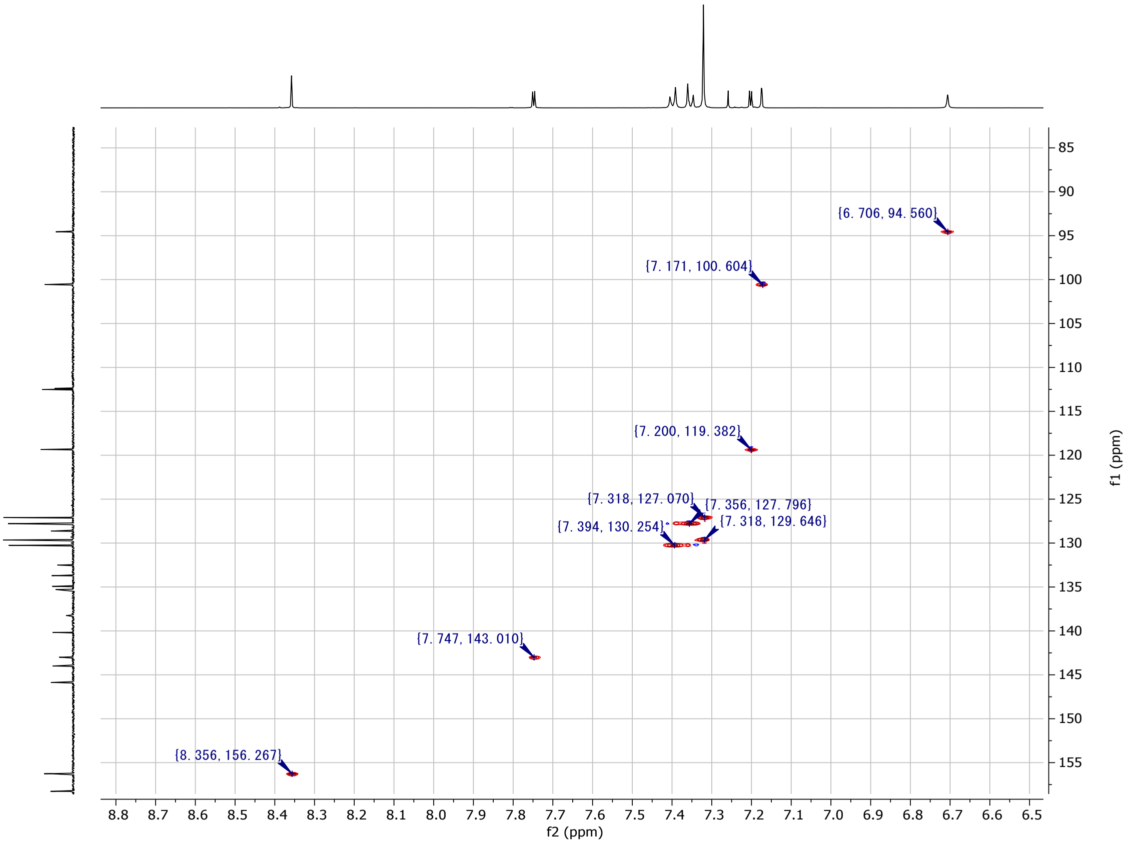

Figure S4. ^{1}H-^{13}C HSQC spectra of compound **7**

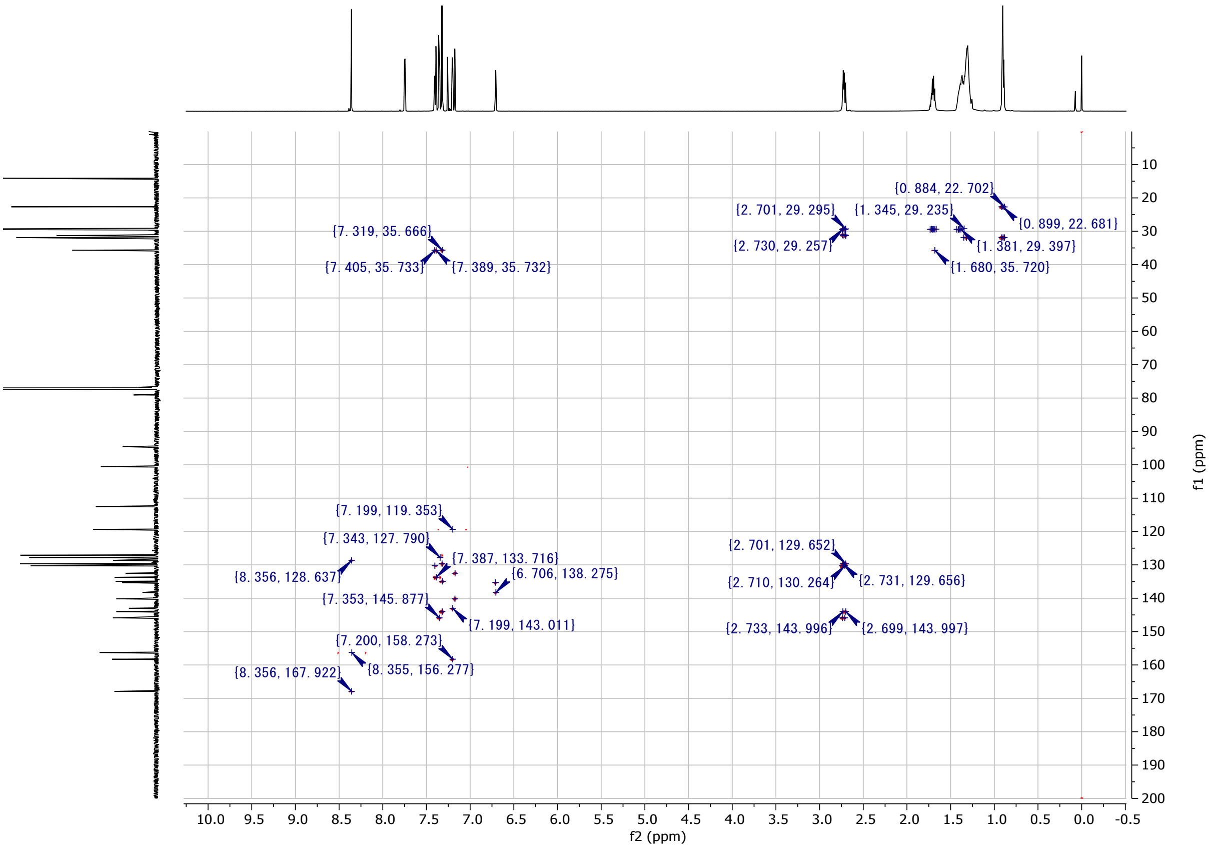

Figure S5. ^{1}H-^{13}C HMBC spectra of compound **7**

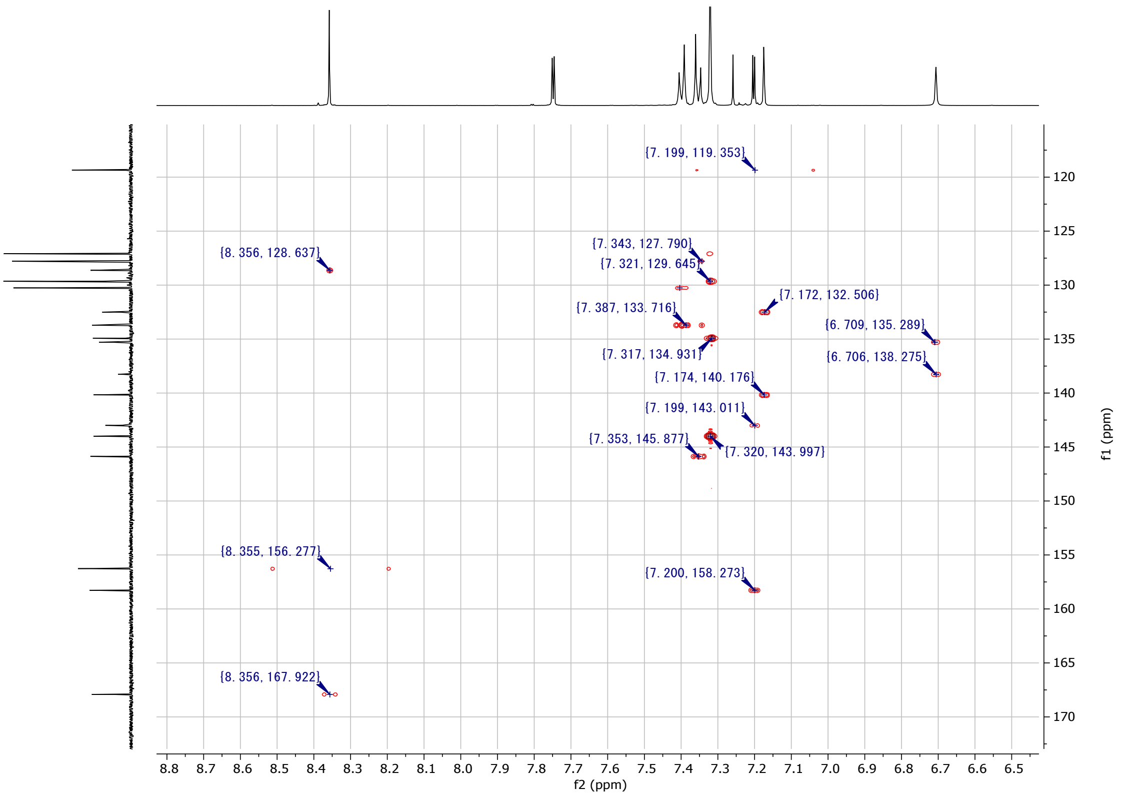

Figure S6. ^{1}H-^{13}C HMBC spectra of compound **7** (aromatic region)

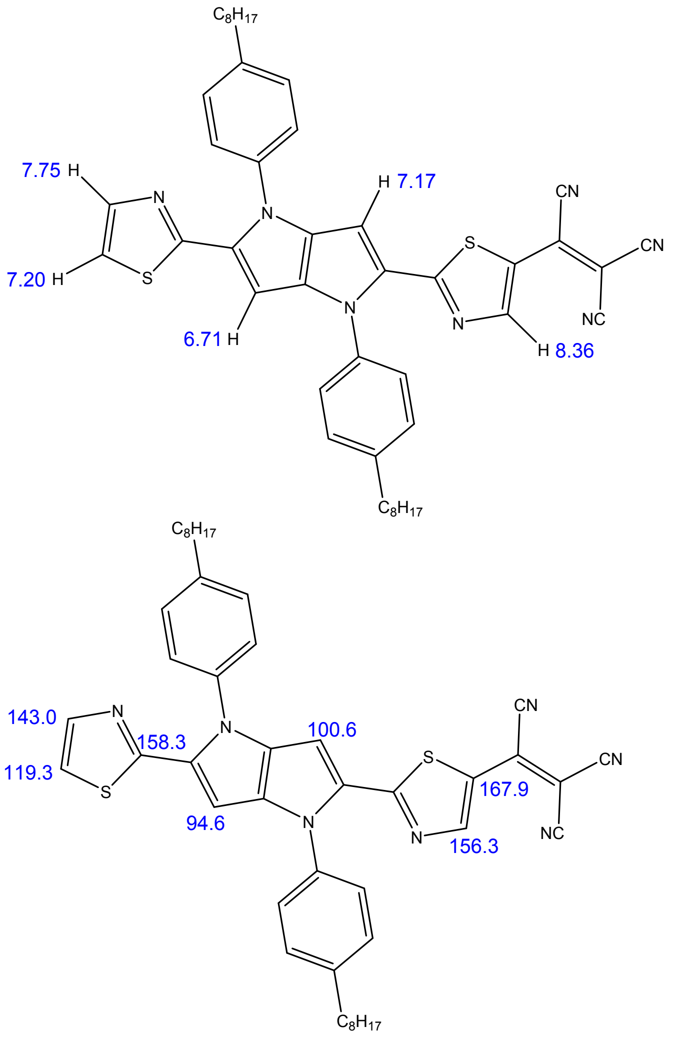

Figure S7. Assignment of signals to the corresponding hydrogen and carbon atoms based on 1D and 2D NMR spectra

2. Proposed mechanism for D−A-type chromophores

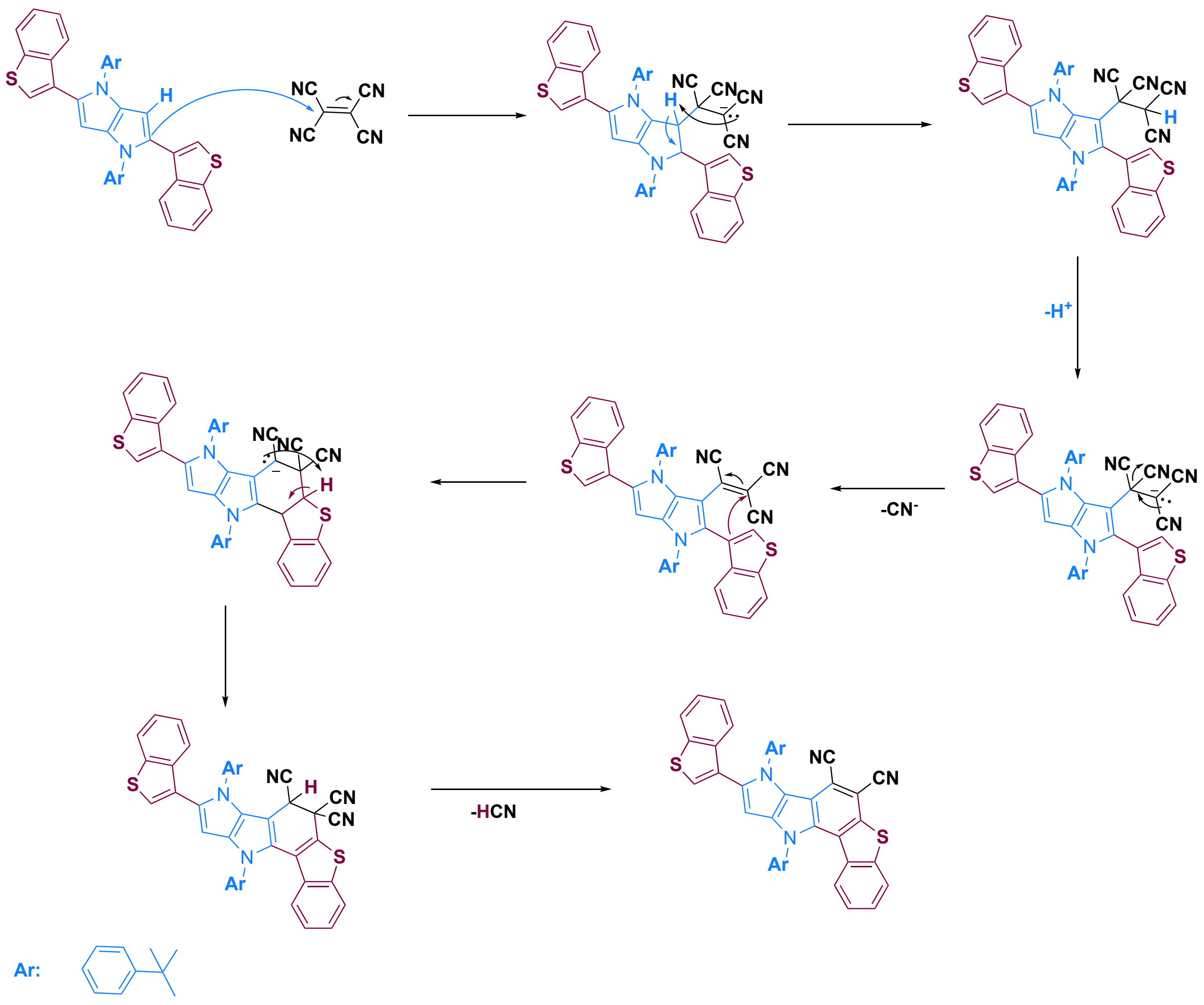

3. Theoretical calculations

3.2.Methods

We have performed the DFT and TD-DFT calculations with Gaussian 16.[4] No simplification was performed, i.e., *t*Bu groups were fully modelled. Conformational search in the ground-state was performed, and we report data for the most stable conformer only. Note that for both **4c** and **4e**, point group symmetry (C_i or C_2) was applied when possible and both led to no imaginary frequency, we therefore selected the C_i structures, the most stable. Default Gaussian16 thresholds and algorithms were used but for an improved optimization threshold

(10^{-5} au on average residual forces), a stricter self-consistent field convergence criterion (10^{-10} a.u.) and the systematic use of the *superfine* DFT integration grid, the denser grid available in Gaussian.

Firstly, the S_0 geometries have been optimized with DFT and the vibrational frequencies have been analytically determined, using the M06-2X *meta*-GGA hybrid exchange-correlation functional.[5] These calculations were performed with the 6-311G(d,p) atomic basis set in gas-phase. Secondly, starting from the optimal ground-state geometries, we have used TD-DFT with the same functional and basis set to optimize the S_1 geometry and compute analytically the vibrational frequencies. All optimized structures correspond to true minima of the potential energy surface. Thirdly, the vertical transition energies were determined with TD-DFT and the same functional, but a diffuse-containing basis set, namely 6-311+G(2d,p), in gas-phase as well as in solution using the cLR^2 variant of the PCM,[6,7] in its *non-equilibrium* limit. Toluene was considered as solvent since this solvent is ideal for PCM applications. It should be stressed here that cLR^2-PCM includes both linear-response and state-specific solvent corrections, and therefore should be well-suited for both local and charge-transfer states, as well as states with a mixed character.

As we are aware of the significant dependency of the TD-DFT results on the selected functional,[8a] the obtained transition energies were also computed using CC2[8b] with the Turbomole 7.3/7.5 codes.[9] The CC2 energies were calculated in gas phase applying the resolution of identity scheme, and using the *aug*-cc-pVDZ atomic basis set. Combining the CC2 and TD-DFT data using a well-known protocol,[10] one can obtain accurate CC2-corrected estimates of the absorption, emission and 0-0 energies that can be straightforwardly compared to experimental values.

To evaluate the possibility of ISC, the minimal S_1 structures (as obtained above) were used. We first evaluated the S-T gaps (several triplets were considered) using the SCS-CC2[11]/*aug*-

cc-pVDZ level of theory, the calculations being performed in the same way as the CC2 calculations described above. This choice of SCS-CC2 rather than CC2 is justified since the former is known to be exceptionally efficient for these gaps.[12] Next, The SOC matrix elements were determined at the M06-2X/*def2*-TZVP level using ORCA.5.0.1[13] with toluene as solvent as modelled with the SMD solvation model. The RI-SOMF(1X) method was used to get the SOCs, and DefGrid3 and TightSCF settings were applied, whereas TDA was turned off.

To perform the rate calculations, we followed the *Fermi Golden Rule* strategy.[14] This required vibrationally resolved spectrum that were determined with the FCClasses 3.01 program.[15,16] We used the time-dependent formulation, applied the FC approximation (HT effects were neglected but for **5b,** though event in that case HT had little impact), and selected the so-called *Vertical Hessian*[17] vibronic models for the calculations. We used a simulation temperature of 298K and internal coordinates, as build by default by FCClasses. During the vibronic calculations, the vibrational contributions and transition dipoles are coming from TD-DFT, whereas the transition energies are CC2-corrected. The radiative and internal conversion rates have been obtained using the same methodology within the TVCF formalism.[18] For the radiative part, we used as broadening a Gaussian having HWHM of 100 cm^{-1}, but this is known to be not important for the radiative rate.[19] For the IC part, we used a 10 cm^{-1} broadening Lorentzian, which is a typical value in the literature[20,21] though we are well aware of the difficulty to interpret such value.[19]

For **4c**, **4e**, and **6a**, the search for the minimum energy crossing point (MECP) between the ground and lowest excited states was achieved at the PCM(Toluene)-M06-2X/6-31G(d) level of theory applying the spin-flip (SF) formalism as implemented in the Q-Chem 5.4 code.[20] The branching plane approach was used to optimize the MECP and a spin contamination threshold of 1.2 was set in order to restrain the search process to singlet states. After the MECP geometries were located the difference in energies between the S_0 FC, S_1, and MECP were

calculated with PCM(Toluene)-SF-TDM06-2X/6-311G(d,p) level of theory (using geometries obtained with conventional TD-DFT for the the S_0 and the S_1 minima, and the SF-TD-DFT ones for the MECP).

3.3.Additional results

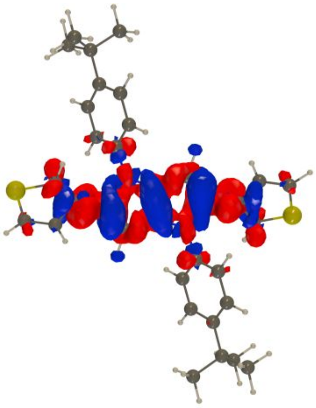

4c (S_0-S_1)

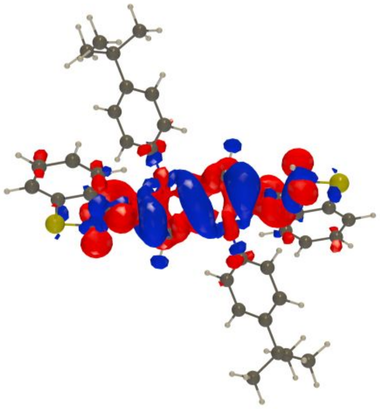

4e (S_0-S_1)

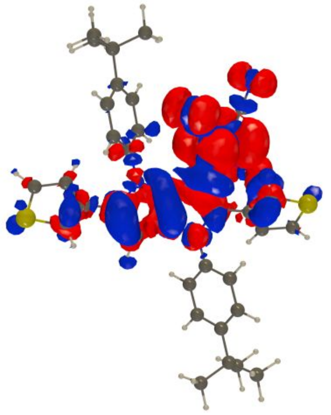

5b (S_0-S_1)

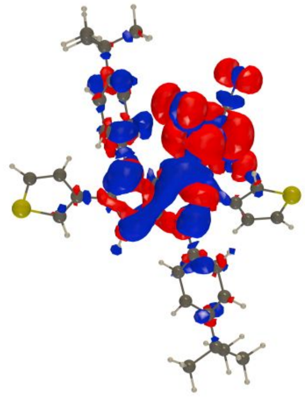

5b (S_0-S_2)

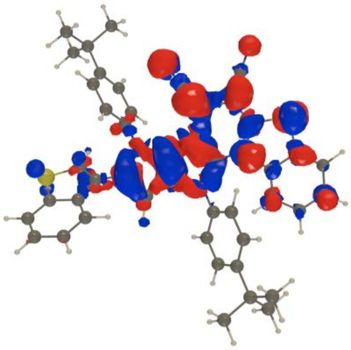

6a (S_0-S_1)

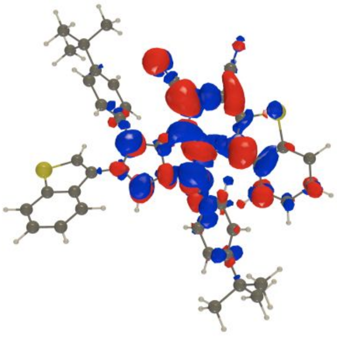

6a (S_0-S_2)

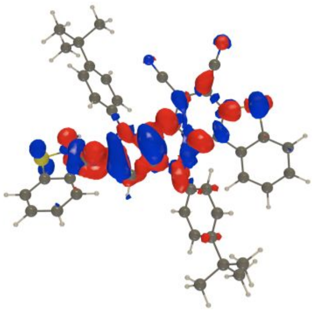

6a (S_0-S_3)

Figure S8: Electron density difference plot corresponding to the absorption of the conjugated compounds. The blue and red lobes correspond to decrease and increase of electron density, respectively. Contour threshold 0.001 au.

We have assessed the possibility of ISC for **4c**, **4e** and **6a**. The results are detailed in the Table below. As can be seen the S_1-T_1 gaps are very large in both **4c** and **4e**, and the more aligned S_1-T_2 have a very small spin-orbit coupling, making ISC rather ineffective. For **6a**, the situation is intermediate so one cannot rule out the presence of ISC, yet the SOC remains smaller than 0.3 cm^{-1}.

Table S1: Singlet-triplet gaps, S_1-T_x, determined on the S_1optimal geometry (in eV) together with the computed spin-orbit couplings (cm^{-1}). For the gaps, a negative value indicates that the triplet is above the singlet.

Comp.	Triplet State	DEST	SOC
**4c**	T_1	1.12	0.22
	T_2	0.03	0.00
**4e**	T_1	1.06	0.24
	T_2	0.34	0.00

	T_3	-0.14	3.30
**6a**	*T_1*	0.33	0.29

In Table S2, we present the computed rates. The radiative and internal conversion rates are determined from vibronic calculations. For the MECP rate, which is part of the non-radiative ones, we used Arrhenius law as suggested in Ref.[20] with a barrier corresponding to the difference between the MECP energy and the average energies of the FC point and S_1 minimum (see representations in Figures S9 and S10). Indeed, in the actual excited-state dynamics, it is hard to determine if all the energy available at the FC point allows to reach the MECP, or if relaxation to the minimal S_1 structure takes place first. The reality is generally in between these two extremes,[22] and as a crude approximation, we simply took the average of these barriers here.

Table S2: Computed rates (in 10^8 s^{-1}) as computed in the FC approximation for the radiative and internal conversion ones (see methods for details), and by the Arrhenius relationship for the MECP.

**Comp.**	**k_r**	**k_{ic}**	**f^b**	**k_{mecp}**	**f^c**
**4c**	4.91	0.12	0.98	5.71	0.46
**4e**	4.14	0.13	0.97	20.60	0.16
**5b**	0.00[a]	1260	0.00	n.d.	n.d.
**6a**	0.25	0.72	0.26		

[a]1.85 x 10^4 in FC, 1.65 x 10^5 in FC-HT; [b] Computed using k_r and k_{ic} only; [c] Computed using k_r, k_{ic} and k_{mecp}.

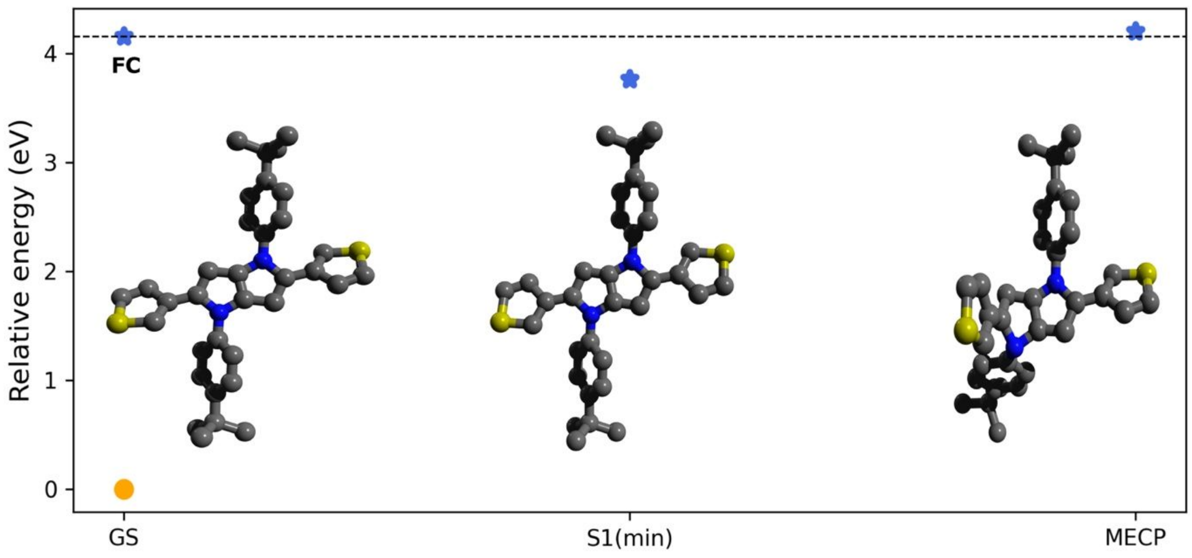

Figure S9: Simplified representation of the structures of **4c** and their relative SF-TD-DFT energies.

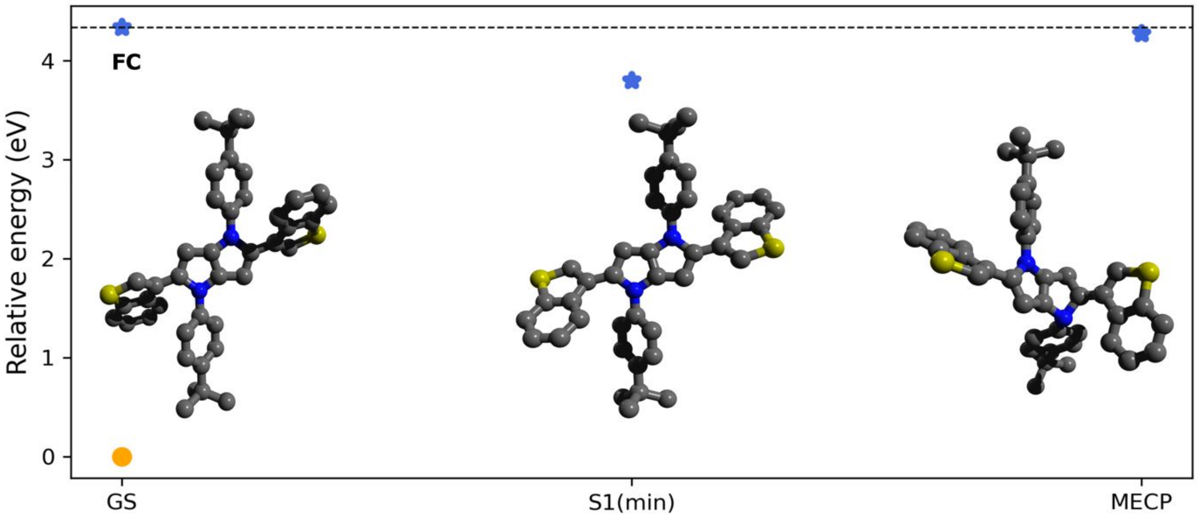

Figure S10: Simplified representation of the structures of **4e** and their relative SF-TD-DFT energies.

3.4.Cartesian coordinates

Below, we provide Cartesian coordinates for the ground and excited-states minima in Å, that have determined at (TD-)DFT level using the approaches detailed in the method section. All

structures are true minima (no imaginary frequency). We also provide the Gibbs free energy for all of them.

4c – S_0 — G= -2221.175702 Eh — n^{imag}=0

C	1.7186740	-1.2488800	0.0883260
C	1.7556610	0.1331810	0.1583340
C	0.4060950	0.5565330	0.0666190
C	-0.4060950	-0.5565330	-0.0666190
C	-1.7186740	1.2488800	-0.0883260
C	-1.7556610	-0.1331810	-0.1583340
H	2.6537320	0.7282840	0.2045870
H	-2.6537320	-0.7282840	-0.2045870
N	0.3976950	-1.6781670	-0.0468880
N	-0.3976950	1.6781670	0.0468880
C	-0.1024030	-3.0048500	0.0082530
C	-1.0566580	-3.4117010	-0.9220880
C	0.3230610	-3.8939450	0.9875850
C	-1.5701920	-4.6989700	-0.8665770
H	-1.3815130	-2.7169650	-1.6883350
C	-0.1871470	-5.1876620	1.0189850
H	1.0561850	-3.5740520	1.7180430
C	-1.1437310	-5.6196840	0.0980930
H	-2.3120310	-4.9911620	-1.6013460
H	0.1701950	-5.8570440	1.7904180
C	0.1024030	3.0048500	-0.0082530

C	1.0566580	3.4117010	0.9220880
C	-0.3230610	3.8939450	-0.9875850
C	1.5701920	4.6989700	0.8665770
H	1.3815130	2.7169650	1.6883350
C	0.1871470	5.1876620	-1.0189850
H	-1.0561850	3.5740520	-1.7180430
C	1.1437310	5.6196840	-0.0980930
H	2.3120310	4.9911620	1.6013460
H	-0.1701950	5.8570440	-1.7904180
C	-1.7309710	-7.0326440	0.1086250
C	-1.1462260	-7.8919590	1.2336590
C	-3.2540910	-6.9491220	0.3051330
C	-1.4258540	-7.7195300	-1.2332840
H	-0.0643360	-8.0075220	1.1302250
H	-1.3579580	-7.4647740	2.2171030
H	-1.5939510	-8.8877870	1.1981700
H	-3.7334110	-6.3876050	-0.4994350
H	-3.6853900	-7.9538980	0.3182670
H	-3.4940430	-6.4582250	1.2514540
H	-1.8387580	-8.7320550	-1.2378600
H	-1.8619920	-7.1743190	-2.0729200
H	-0.3471670	-7.7865190	-1.3951690
C	1.7309710	7.0326440	-0.1086250
C	1.1462260	7.8919590	-1.2336590
C	1.4258540	7.7195300	1.2332840

C	3.2540910	6.9491220	-0.3051330
H	1.3579580	7.4647740	-2.2171030
H	0.0643360	8.0075220	-1.1302250
H	1.5939510	8.8877870	-1.1981700
H	1.8619920	7.1743190	2.0729200
H	1.8387580	8.7320550	1.2378600
H	0.3471670	7.7865190	1.3951690
H	3.6853900	7.9538980	-0.3182670
H	3.7334110	6.3876050	0.4994350
H	3.4940430	6.4582250	-1.2514540
C	2.8685580	-2.1513780	0.0466070
C	4.0813330	-1.8872810	0.7665480
C	2.9590810	-3.3031040	-0.6883830
C	5.0359940	-2.8316060	0.5596900
H	4.2099470	-1.0342940	1.4191350
S	4.4872060	-4.0669990	-0.5072590
H	2.2111720	-3.7413530	-1.3310910
H	6.0269620	-2.8842810	0.9826220
C	-2.8685580	2.1513780	-0.0466070
C	-4.0813330	1.8872810	-0.7665480
C	-2.9590810	3.3031040	0.6883830
C	-5.0359940	2.8316060	-0.5596900
H	-4.2099470	1.0342940	-1.4191350
S	-4.4872060	4.0669990	0.5072590
H	-2.2111720	3.7413530	1.3310910

H	-6.0269620	2.8842810	-0.9826220

4c – S_1 — G= -2221.050173 Eh — n^{imag}=0

C	1.7129900	-1.2532330	0.1975620
C	1.6971650	0.1702410	0.3798780
C	0.4022730	0.5695840	0.1529030
C	-0.4022730	-0.5695840	-0.1529030
C	-1.7129900	1.2532330	-0.1975620
C	-1.6971650	-0.1702410	-0.3798780
H	2.5679250	0.7754200	0.5692740
H	-2.5679250	-0.7754200	-0.5692740
N	0.4095440	-1.6902780	-0.0993070
N	-0.4095440	1.6902780	0.0993070
C	-0.0994600	-3.0077470	-0.0401840
C	-1.0726690	-3.4051510	-0.9595210
C	0.3236520	-3.9038420	0.9402470
C	-1.6030960	-4.6829860	-0.8962680
H	-1.3907020	-2.7138730	-1.7318050
C	-0.2110190	-5.1882070	0.9788730
H	1.0632120	-3.5944110	1.6679930
C	-1.1826320	-5.6094980	0.0699770
H	-2.3530550	-4.9665870	-1.6261640
H	0.1418470	-5.8601400	1.7504830
C	0.0994600	3.0077470	0.0401840
C	1.0726690	3.4051510	0.9595210

C	-0.3236520	3.9038420	-0.9402470
C	1.6030960	4.6829860	0.8962680
H	1.3907020	2.7138730	1.7318050
C	0.2110190	5.1882070	-0.9788730
H	-1.0632120	3.5944110	-1.6679930
C	1.1826320	5.6094980	-0.0699770
H	2.3530550	4.9665870	1.6261640
H	-0.1418470	5.8601400	-1.7504830
C	-1.7925210	-7.0120520	0.0897450
C	-1.2127340	-7.8772380	1.2128010
C	-3.3126550	-6.9040950	0.2984950
C	-1.5109190	-7.7094160	-1.2521040
H	-0.1337810	-8.0108430	1.1005850
H	-1.4086960	-7.4427110	2.1963270
H	-1.6770930	-8.8657380	1.1850990
H	-3.7889770	-6.3353480	-0.5028520
H	-3.7607520	-7.9015310	0.3161850
H	-3.5366430	-6.4081450	1.2461090
H	-1.9424660	-8.7142670	-1.2505930
H	-1.9432110	-7.1587020	-2.0901980
H	-0.4349320	-7.7965520	-1.4221160
C	1.7925210	7.0120520	-0.0897450
C	1.2127340	7.8772380	-1.2128010
C	1.5109190	7.7094160	1.2521040
C	3.3126550	6.9040950	-0.2984950

H	1.4086960	7.4427110	-2.1963270
H	0.1337810	8.0108430	-1.1005850
H	1.6770930	8.8657380	-1.1850990
H	1.9432110	7.1587020	2.0901980
H	1.9424660	8.7142670	1.2505930
H	0.4349320	7.7965520	1.4221160
H	3.7607520	7.9015310	-0.3161850
H	3.7889770	6.3353480	0.5028520
H	3.5366430	6.4081450	-1.2461090
C	2.8727970	-2.0737570	0.1355440
C	4.1558840	-1.6127070	0.6174470
C	2.9830770	-3.3525000	-0.4144970
C	5.1526680	-2.5064900	0.4313900
H	4.3035670	-0.6570790	1.1016060
S	4.5887380	-3.9495730	-0.3424810
H	2.2197260	-3.9413040	-0.8948110
H	6.1900310	-2.4166110	0.7135880
C	-2.8727970	2.0737570	-0.1355440
C	-4.1558840	1.6127070	-0.6174470
C	-2.9830770	3.3525000	0.4144970
C	-5.1526680	2.5064900	-0.4313900
H	-4.3035670	0.6570790	-1.1016060
S	-4.5887380	3.9495730	0.3424810
H	-2.2197260	3.9413040	0.8948110
H	-6.1900310	2.4166110	-0.7135880

4e – S_0 —G= -2528.335110 Eh— n^{imag}=0

C	1.4261660	-1.5279230	0.3862440
C	1.7392150	-0.1873200	0.2682140
C	0.5066200	0.4713860	0.0198480
C	-0.5066200	-0.4713860	-0.0198480
C	-1.4261660	1.5279230	-0.3862440
C	-1.7392150	0.1873200	-0.2682140
H	2.7379300	0.2168890	0.3052340
H	-2.7379300	-0.2168890	-0.3052340
N	0.0517090	-1.7114050	0.2154950
N	-0.0517090	1.7114050	-0.2154950
C	-0.6828430	-2.9170000	0.3408240
C	-1.6950710	-3.1933170	-0.5780360
C	-0.4047380	-3.8321590	1.3479780
C	-2.4001260	-4.3829730	-0.4917470
H	-1.9050680	-2.4826500	-1.3694380
C	-1.1088040	-5.0309760	1.4087730
H	0.3664870	-3.6149880	2.0765150
C	-2.1159850	-5.3369930	0.4934490
H	-3.1767190	-4.5766820	-1.2231390
H	-0.8567540	-5.7277890	2.1972580
C	0.6828430	2.9170000	-0.3408240
C	1.6950710	3.1933170	0.5780360
C	0.4047380	3.8321590	-1.3479780

C	2.4001260	4.3829730	0.4917470
H	1.9050680	2.4826500	1.3694380
C	1.1088040	5.0309760	-1.4087730
H	-0.3664870	3.6149880	-2.0765150
C	2.1159850	5.3369930	-0.4934490
H	3.1767190	4.5766820	1.2231390
H	0.8567540	5.7277890	-2.1972580
C	-2.8947490	-6.6533090	0.5204280
C	-2.4555420	-7.5591910	1.6747320
C	-4.3957100	-6.3593800	0.6796880
C	-2.6568920	-7.4036730	-0.8012630
H	-1.3995210	-7.8287800	1.5940270
H	-2.6198580	-7.0821500	2.6442920
H	-3.0393150	-8.4823340	1.6525390
H	-4.7782060	-5.7552300	-0.1455930
H	-4.9600600	-7.2957620	0.7007230
H	-4.5861600	-5.8209590	1.6113340
H	-3.1970120	-8.3545370	-0.7954740
H	-3.0035440	-6.8240660	-1.6595340
H	-1.5925350	-7.6102670	-0.9387770
C	2.8947490	6.6533090	-0.5204280
C	2.4555420	7.5591910	-1.6747320
C	2.6568920	7.4036730	0.8012630
C	4.3957100	6.3593800	-0.6796880
H	2.6198580	7.0821500	-2.6442920

H	1.3995210	7.8287800	-1.5940270
H	3.0393150	8.4823340	-1.6525390
H	3.0035440	6.8240660	1.6595340
H	3.1970120	8.3545370	0.7954740
H	1.5925350	7.6102670	0.9387770
H	4.9600600	7.2957620	-0.7007230
H	4.7782060	5.7552300	0.1455930
H	4.5861600	5.8209590	-1.6113340
C	2.3557830	-2.6392600	0.6087600
C	2.4524700	-3.8290550	-0.2159340
C	3.2626590	-2.6507460	1.6183350
C	3.4448220	-4.7098040	0.2471660
S	4.2632640	-4.0687040	1.6490540
H	3.3856830	-1.8952250	2.3802690
C	-2.3557830	2.6392600	-0.6087600
C	-2.4524700	3.8290550	0.2159340
C	-3.2626590	2.6507460	-1.6183350
C	-3.4448220	4.7098040	-0.2471660
S	-4.2632640	4.0687040	-1.6490540
H	-3.3856830	1.8952250	-2.3802690
C	-3.7066300	5.9241280	0.3925050
C	-2.9606550	6.2481710	1.5102760
H	-4.4749050	6.5915510	0.0215370
H	-3.1430930	7.1864250	2.0200200
C	-1.7231670	4.1709070	1.3653920

C	-1.9768550	5.3718450	1.9978830
H	-0.9692070	3.4924740	1.7469380
H	-1.4137060	5.6419510	2.8828390
C	1.7231670	-4.1709070	-1.3653920
C	1.9768550	-5.3718450	-1.9978830
H	0.9692070	-3.4924740	-1.7469380
H	1.4137060	-5.6419510	-2.8828390
C	3.7066300	-5.9241280	-0.3925050
C	2.9606550	-6.2481710	-1.5102760
H	4.4749050	-6.5915510	-0.0215370
H	3.1430930	-7.1864250	-2.0200200

4e – S_1 — G= -2528.207974 Eh — n^{imag}=0

C	1.4472900	-1.5319780	0.3266380
C	1.6791890	-0.1228540	0.4658360
C	0.4882660	0.4923370	0.1547970
C	-0.4882660	-0.4923370	-0.1547970
C	-1.4472900	1.5319780	-0.3266380
C	-1.6791890	0.1228540	-0.4658360
H	2.6446300	0.3117360	0.6667260
H	-2.6446300	-0.3117360	-0.6667260
N	0.1043190	-1.7420990	-0.0134540
N	-0.1043190	1.7420990	0.0134540
C	-0.6520980	-2.9273390	0.1527760

C	-1.7027900	-3.1864200	-0.7297490
C	-0.3697100	-3.8368250	1.1664540
C	-2.4431650	-4.3502940	-0.5984320
H	-1.9116930	-2.4892580	-1.5332010
C	-1.1175090	-5.0034170	1.2766280
H	0.4380490	-3.6390930	1.8603280
C	-2.1651210	-5.2924740	0.3996220
H	-3.2468390	-4.5316910	-1.3033780
H	-0.8664260	-5.6921330	2.0725460
C	0.6520980	2.9273390	-0.1527760
C	1.7027900	3.1864200	0.7297490
C	0.3697100	3.8368250	-1.1664540
C	2.4431650	4.3502940	0.5984320
H	1.9116930	2.4892580	1.5332010
C	1.1175090	5.0034170	-1.2766280
H	-0.4380490	3.6390930	-1.8603280
C	2.1651210	5.2924740	-0.3996220
H	3.2468390	4.5316910	1.3033780
H	0.8664260	5.6921330	-2.0725460
C	-2.9900380	-6.5777240	0.4803010
C	-2.5518840	-7.4712280	1.6446150
C	-4.4744600	-6.2249630	0.6730700
C	-2.8175940	-7.3681560	-0.8281800
H	-1.5095540	-7.7831080	1.5413660
H	-2.6698680	-6.9644240	2.6058020

H	-3.1703570	-8.3715820	1.6625230
H	-4.8575760	-5.6257520	-0.1555520
H	-5.0718490	-7.1390370	0.7313530
H	-4.6190740	-5.6588880	1.5964930
H	-3.3943850	-8.2962930	-0.7854130
H	-3.1638050	-6.7953120	-1.6912050
H	-1.7662700	-7.6207150	-0.9877240
C	2.9900380	6.5777240	-0.4803010
C	2.5518840	7.4712280	-1.6446150
C	2.8175940	7.3681560	0.8281800
C	4.4744600	6.2249630	-0.6730700
H	2.6698680	6.9644240	-2.6058020
H	1.5095540	7.7831080	-1.5413660
H	3.1703570	8.3715820	-1.6625230
H	3.1638050	6.7953120	1.6912050
H	3.3943850	8.2962930	0.7854130
H	1.7662700	7.6207150	0.9877240
H	5.0718490	7.1390370	-0.7313530
H	4.8575760	5.6257520	0.1555520
H	4.6190740	5.6588880	-1.5964930
C	2.4422150	-2.5415910	0.4671580
C	2.5326780	-3.8199800	-0.2312060
C	3.5333610	-2.3530640	1.3087740
C	3.6703160	-4.5517610	0.1616390
S	4.6359410	-3.6776800	1.3270710

H	3.6858420	-1.5272050	1.9865050
C	-2.4422150	2.5415910	-0.4671580
C	-2.5326780	3.8199800	0.2312060
C	-3.5333610	2.3530640	-1.3087740
C	-3.6703160	4.5517610	-0.1616390
S	-4.6359410	3.6776800	-1.3270710
H	-3.6858420	1.5272050	-1.9865050
C	-3.9815110	5.7958800	0.3859920
C	-3.1452830	6.3143210	1.3607870
H	-4.8621690	6.3366510	0.0610830
H	-3.3652670	7.2807390	1.7976690
C	-1.7279430	4.3513560	1.2504720
C	-2.0326610	5.5862520	1.7985790
H	-0.8758710	3.7963250	1.6192100
H	-1.4020560	5.9901310	2.5813290
C	1.7279430	-4.3513560	-1.2504720
C	2.0326610	-5.5862520	-1.7985790
H	0.8758710	-3.7963250	-1.6192100
H	1.4020560	-5.9901310	-2.5813290
C	3.9815110	-5.7958800	-0.3859920
C	3.1452830	-6.3143210	-1.3607870
H	4.8621690	-6.3366510	-0.0610830
H	3.3652670	-7.2807390	-1.7976690

5b – S_0 — G= -2575.246024 Eh — n^{imag}=0

C	0.8825800	2.4593670	-0.1051550
C	-0.4997000	2.3807660	-0.1100750
C	-0.7959750	0.9970990	-0.0434960
C	0.3861410	0.2872470	0.0143930
C	-1.3252340	-1.1812650	0.0275630
C	0.0800140	-1.1081700	0.0492890
H	-1.1706290	3.2197230	-0.1960690
N	1.4262770	1.1794020	-0.0437270
N	-1.8424690	0.0898660	-0.0218810
C	2.7992070	0.7993540	-0.0224980
C	3.2687520	-0.0694680	-1.0045300
C	3.6415970	1.2297440	0.9895600
C	4.5724810	-0.5350630	-0.9387060
H	2.6043920	-0.3827780	-1.8031880
C	4.9528200	0.7663430	1.0329530
H	3.2654570	1.9057260	1.7484240
C	5.4393600	-0.1376380	0.0868260
H	4.9146100	-1.2284410	-1.6983680
H	5.5874780	1.1054400	1.8406290
C	-3.2168870	0.4662190	0.0342810
C	-3.7008920	1.3770340	-0.9008490
C	-4.0593690	-0.0413530	1.0132190
C	-5.0301920	1.7670220	-0.8505290
H	-3.0366410	1.7631780	-1.6655630
C	-5.3942560	0.3498710	1.0404930

H	-3.6758990	-0.7399960	1.7468030
C	-5.9087460	1.2595860	0.1150160
H	-5.3886850	2.4751910	-1.5885970
H	-6.0302100	-0.0655360	1.8107380
C	6.8490730	-0.7267200	0.1501200
C	7.6613620	-0.1557810	1.3162860
C	6.7340190	-2.2497820	0.3366400
C	7.5980690	-0.4207810	-1.1573590
H	7.7778390	0.9279970	1.2329600
H	7.1972210	-0.3842140	2.2787830
H	8.6587450	-0.6011470	1.3131150
H	6.2110290	-2.7186910	-0.4996320
H	7.7304300	-2.6948820	0.4052980
H	6.1834440	-2.4839000	1.2508540
H	8.6055850	-0.8432300	-1.1159700
H	7.0918840	-0.8489410	-2.0248330
H	7.6833520	0.6580180	-1.3104930
C	-7.3696100	1.7136590	0.1201710
C	-8.1747190	1.0547680	1.2441980
C	-7.4259720	3.2385870	0.3126530
C	-8.0195680	1.3428000	-1.2236550
H	-8.1910360	-0.0333380	1.1429470
H	-7.7726800	1.3073070	2.2286110
H	-9.2070180	1.4094560	1.2041810
H	-6.9095220	3.7660070	-0.4919910

H	-8.4662990	3.5745950	0.3219580
H	-6.9627370	3.5254100	1.2598680
H	-9.0663110	1.6582970	-1.2308570
H	-7.5164310	1.8278890	-2.0626620
H	-7.9841960	0.2624620	-1.3840930
C	1.7151170	3.6610790	-0.1783820
C	2.8982310	3.8069570	-0.9781630
C	1.4091280	4.8129890	0.4932810
C	3.4376800	5.0504350	-0.8881070
H	3.3105190	3.0190600	-1.5935880
S	2.5299460	6.0709010	0.1619080
H	0.5913640	4.9743060	1.1788790
H	4.3141890	5.4341410	-1.3863230
C	-2.1772950	-2.3683960	0.1104050
C	-3.2748520	-2.6376350	-0.7711800
C	-1.9810290	-3.3749840	1.0151060
C	-3.8643530	-3.8323560	-0.5106120
H	-3.5821760	-1.9722520	-1.5664050
S	-3.1039620	-4.6526680	0.7992980
H	-1.2402600	-3.4239670	1.7992700
H	-4.6934300	-4.2928490	-1.0243890
C	1.0031680	-2.2182500	0.1387810
C	0.9439770	-3.3695580	-0.5910820
C	2.0736230	-2.1027380	1.0985510
N	2.8981370	-2.0300600	1.8967810

C	1.8544360	-4.4491630	-0.3624260
N	2.5891090	-5.3167630	-0.1888200
C	0.0011700	-3.5504430	-1.6501840
N	-0.7209490	-3.7138830	-2.5304080

5b – S_1 — G= -2575.185312 Eh— n^{imag}=0

C	-0.7141490	2.4203550	0.1385390
C	0.7044360	2.2722390	0.1563500
C	0.9283740	0.9131700	0.0927110
C	-0.3117500	0.2448900	0.0183700
C	1.3137620	-1.2786740	-0.0622370
C	-0.1180670	-1.1138910	-0.1076940
H	1.4096940	3.0813700	0.2493320
N	-1.3109610	1.1925260	0.0680090
N	1.9233130	-0.0479260	0.0492640
C	-2.6981340	0.8459120	-0.0667640
C	-3.3357160	0.2129420	0.9935290
C	-3.3516610	1.0582520	-1.2685090
C	-4.6330890	-0.2422380	0.8228650
H	-2.8044710	0.0569460	1.9259230
C	-4.6618510	0.6125090	-1.4161860
H	-2.8342510	1.5460360	-2.0863880
C	-5.3189890	-0.0632800	-0.3861800
H	-5.1064820	-0.7710000	1.6417290
H	-5.1540350	0.7744610	-2.3655160

C	3.3145540	0.2604650	-0.0493340
C	3.9067700	1.0340150	0.9435070
C	4.0597610	-0.1913950	-1.1280950
C	5.2545400	1.3458650	0.8471920
H	3.3152480	1.3698400	1.7877400
C	5.4140910	0.1175060	-1.2006620
H	3.5861820	-0.7868390	-1.9000110
C	6.0394800	0.8910860	-0.2202980
H	5.7025120	1.9468260	1.6298980
H	5.9767790	-0.2519830	-2.0473830
C	-6.7213040	-0.6509020	-0.5454930
C	-7.3419610	-0.3040480	-1.9020820
C	-6.6195230	-2.1820760	-0.4297450
C	-7.6410310	-0.1070690	0.5602770
H	-7.4307510	0.7770150	-2.0406500
H	-6.7581120	-0.7162350	-2.7285570
H	-8.3448640	-0.7329940	-1.9586310
H	-6.2128020	-2.4927880	0.5344840
H	-7.6105470	-2.6296740	-0.5454820
H	-5.9635390	-2.5840330	-1.2058300
H	-8.6442470	-0.5256240	0.4450350
H	-7.2818120	-0.3741050	1.5558020
H	-7.7145030	0.9821590	0.5031500
C	7.5248600	1.2531760	-0.2721730
C	8.2180800	0.6691030	-1.5066420

C	7.6711020	2.7838540	-0.3116790
C	8.2233090	0.7034320	0.9831790
H	8.1680260	-0.4226060	-1.5180250
H	7.7786040	1.0483850	-2.4326230
H	9.2721980	0.9544550	-1.4967290
H	7.2385060	3.2540050	0.5738390
H	8.7292410	3.0549980	-0.3530050
H	7.1753720	3.1965610	-1.1937390
H	9.2873180	0.9524490	0.9555260
H	7.8031420	1.1265840	1.8978740
H	8.1256550	-0.3837110	1.0345730
C	-1.4425050	3.6709600	0.1949700
C	-2.7898160	3.8613160	0.6580400
C	-0.8606250	4.8585460	-0.1877880
C	-3.1679780	5.1633890	0.6116710
H	-3.4284180	3.0708390	1.0232220
S	-1.9181170	6.1816320	0.0008720
H	0.1277680	5.0065320	-0.5953010
H	-4.1117060	5.5919250	0.9102580
C	2.0308360	-2.5235850	-0.0357110
C	3.2849880	-2.7545560	0.6340310
C	1.5479160	-3.6739450	-0.6329750
C	3.6934260	-4.0408620	0.5262770
H	3.8224100	-2.0031650	1.1942330
S	2.5909140	-4.9961090	-0.4005060

H	0.6511740	-3.7913070	-1.2210440
H	4.5721200	-4.4975660	0.9537890
C	-1.1443830	-2.1633800	-0.1187240
C	-1.7089970	-2.5887220	1.1186550
C	-1.6119730	-2.6258910	-1.3551560
N	-1.9438830	-3.0128010	-2.3981250
C	-2.8586920	-3.4062140	1.1722840
N	-3.8125700	-4.0592670	1.2106470
C	-1.1975320	-2.0922560	2.3326750
N	-0.7686850	-1.6348360	3.3074480

6a – S_0 — G= -2789.029678 Eh — n^{imag}=0

C	-1.4762690	-1.2619560	-0.1887000
C	-0.0763620	-1.5357210	0.0007340
C	0.5796910	-0.2887790	-0.2408660
C	-0.3868870	0.6746970	-0.4540980
C	1.5877430	1.6952210	-0.4024150
C	0.2264100	1.9399750	-0.5362620
H	-0.2216770	2.9034570	-0.7158330
N	-1.6497430	0.0907520	-0.4280270
N	1.8032860	0.3391310	-0.2164420
C	-2.8193300	0.8628480	-0.1845350
C	-3.1445020	1.8966860	-1.0594810
C	-3.6244400	0.6045400	0.9151670
C	-4.2905070	2.6442410	-0.8380980

H	-2.5124950	2.0881140	-1.9193530
C	-4.7791660	1.3549830	1.1152480
H	-3.3605030	-0.1907470	1.6026950
C	-5.1411020	2.3838560	0.2451390
H	-4.5319090	3.4382370	-1.5354870
H	-5.3967520	1.1203430	1.9718690
C	3.0681770	-0.3219310	-0.1574340
C	3.4490920	-1.1500860	-1.2081420
C	3.9051420	-0.1372680	0.9299360
C	4.6663970	-1.8085440	-1.1487460
H	2.7843320	-1.2784590	-2.0555020
C	5.1269560	-0.8004240	0.9731800
H	3.5970990	0.5170300	1.7369710
C	5.5270920	-1.6556150	-0.0548750
H	4.9418230	-2.4652290	-1.9657700
H	5.7602210	-0.6509100	1.8372570
C	-6.4110970	3.2169700	0.4260180
C	-7.2061170	2.7942480	1.6649840
C	-6.0318040	4.6998800	0.5753660
C	-7.3091280	3.0387770	-0.8101310
H	-7.5285670	1.7520140	1.5997040
H	-6.6225480	2.9218480	2.5802090
H	-8.1001550	3.4160000	1.7495000
H	-5.5044470	5.0730360	-0.3049370
H	-6.9336460	5.3034740	0.7078980

H	-5.3877480	4.8473160	1.4457100
H	-8.2253390	3.6238540	-0.6937590
H	-6.8103210	3.3724810	-1.7225090
H	-7.5844470	1.9885810	-0.9365330
C	6.8375310	-2.4426090	-0.0145330
C	7.6540420	-2.1409560	1.2456500
C	6.5082680	-3.9453270	-0.0324020
C	7.6914170	-2.0850980	-1.2422370
H	7.9235420	-1.0833110	1.3074410
H	7.1089770	-2.4161710	2.1518400
H	8.5791050	-2.7214660	1.2234620
H	5.9830280	-4.2297610	-0.9466000
H	7.4304510	-4.5302540	0.0248120
H	5.8713720	-4.2082820	0.8149260
H	8.6273260	-2.6500030	-1.2219410
H	7.1771860	-2.3227160	-2.1756860
H	7.9330830	-1.0191220	-1.2478930
C	-2.4269520	-2.3005710	-0.1641390
C	-3.8342640	-2.3603080	-0.5502880
C	-1.9744190	-3.5519030	0.2968430
C	-4.3967970	-3.6143870	-0.2449470
S	-3.2414430	-4.7469800	0.4206430
C	2.6794790	2.6664990	-0.4960190
C	2.6292410	3.9767020	0.1268670
C	3.8365210	2.4790550	-1.1841630

C	3.7844130	4.7312800	-0.1487740
S	4.9099940	3.8358780	-1.1333810
H	4.1309440	1.6015470	-1.7406570
C	3.9595060	6.0219210	0.3552950
C	2.9620390	6.5559090	1.1496900
H	4.8561810	6.5866430	0.1309980
H	3.0753640	7.5559680	1.5498950
C	1.6398560	4.5346520	0.9527310
C	1.8107810	5.8115680	1.4518960
H	0.7583250	3.9578670	1.2047560
H	1.0498670	6.2431810	2.0905590
C	-5.9535770	-1.7343260	-1.5215260
H	-6.5601460	-1.0101690	-2.0511570
C	-5.7279140	-3.9200510	-0.5201830
C	-6.5070140	-2.9636430	-1.1476970
H	-6.1336230	-4.8908800	-0.2638120
H	-7.5436830	-3.1814130	-1.3735370
C	0.3297820	-2.8108940	0.4210070
C	-0.6327150	-3.8128690	0.6035310
C	-0.2642090	-5.1094050	1.0801740
N	-0.0093290	-6.1650020	1.4606910
C	1.6894750	-3.1172890	0.7515720
N	2.7553540	-3.4213630	1.0604950
C	-4.6339970	-1.4315910	-1.2352510
H	-4.2268790	-0.4868370	-1.5632630

6a – S_1 — G= -2788.922333 Eh — n^{imag}=0

C	-1.4104510	-1.2412760	-0.1499580
C	-0.0399540	-1.5143100	0.0536890
C	0.6045810	-0.2634030	-0.1370600
C	-0.3912660	0.7441210	-0.3298870
C	1.6012950	1.7304000	-0.2988260
C	0.2301280	1.9895820	-0.3910830
H	-0.2118740	2.9570090	-0.5574850
N	-1.6101180	0.1560220	-0.3279710
N	1.8152830	0.3456270	-0.1500010
C	-2.8121200	0.8770130	-0.0877160
C	-3.1464580	1.9423490	-0.9198520
C	-3.6422690	0.5237920	0.9664890
C	-4.3283180	2.6321500	-0.6999960
H	-2.5000770	2.1980220	-1.7519780
C	-4.8293420	1.2202080	1.1649790
H	-3.3733490	-0.3037610	1.6122300
C	-5.2026420	2.2805210	0.3373920
H	-4.5805450	3.4495340	-1.3657010
H	-5.4668980	0.9144570	1.9835410
C	3.0807240	-0.3208890	-0.0991770
C	3.4607490	-1.1179450	-1.1733270
C	3.9090520	-0.1633270	0.9979920
C	4.6781340	-1.7759630	-1.1265540

H	2.7972720	-1.2269740	-2.0240140
C	5.1292920	-0.8287880	1.0271780
H	3.5953360	0.4625250	1.8249980
C	5.5325730	-1.6544520	-0.0237050
H	4.9545000	-2.4142420	-1.9572960
H	5.7560160	-0.7101940	1.9005000
C	-6.5142290	3.0475650	0.5120890
C	-7.3269980	2.5352800	1.7048550
C	-6.2092820	4.5381630	0.7363560
C	-7.3632410	2.8822720	-0.7600950
H	-7.6023930	1.4848700	1.5823850
H	-6.7771840	2.6444560	2.6431070
H	-8.2491210	3.1146300	1.7884300
H	-5.6724040	4.9732020	-0.1091480
H	-7.1421940	5.0937820	0.8631150
H	-5.6015320	4.6768620	1.6338880
H	-8.3097920	3.4180390	-0.6493930
H	-6.8523500	3.2790570	-1.6400010
H	-7.5828640	1.8269310	-0.9401940
C	6.8352100	-2.4537150	0.0063010
C	7.6449920	-2.1907300	1.2793530
C	6.4873120	-3.9516730	-0.0491480
C	7.7011480	-2.0759640	-1.2067100
H	7.9222800	-1.1371090	1.3709760
H	7.0918550	-2.4864920	2.1739890

H	8.5657760	-2.7773710	1.2477470
H	5.9761660	-4.2102090	-0.9788150
H	7.4016250	-4.5482130	0.0127540
H	5.8291810	-4.2234470	0.7786530
H	8.6288790	-2.6542010	-1.1948310
H	7.1904340	-2.2831030	-2.1492950
H	7.9583590	-1.0137060	-1.1835600
C	-2.3845410	-2.2737300	-0.1949810
C	-3.7554530	-2.3344830	-0.6497650
C	-1.9164130	-3.5370410	0.2611850
C	-4.3351470	-3.5960860	-0.3795770
S	-3.1847340	-4.7199500	0.3312640
C	2.6785930	2.6859580	-0.3948020
C	2.5742250	4.0641120	0.0640150
C	3.8903880	2.4444210	-0.9920300
C	3.7300450	4.8015080	-0.2481700
S	4.9165680	3.8164160	-1.0647620
H	4.2271120	1.5207320	-1.4376930
C	3.8757880	6.1421550	0.1087720
C	2.8428990	6.7496110	0.7990940
H	4.7767880	6.6875640	-0.1432190
H	2.9300960	7.7901270	1.0859110
C	1.5558800	4.6954040	0.7952520
C	1.6945330	6.0248890	1.1484320
H	0.6795810	4.1409940	1.1051100

H	0.9089850	6.5108520	1.7135950
C	-5.8568920	-1.7140030	-1.6803370
H	-6.4483910	-0.9827660	-2.2185440
C	-5.6461070	-3.9078020	-0.7017950
C	-6.4195730	-2.9466090	-1.3461220
H	-6.0517150	-4.8853800	-0.4705950
H	-7.4464200	-3.1679560	-1.6080830
C	0.4228910	-2.8124030	0.4272900
C	-0.5829590	-3.8237050	0.5858730
C	-0.2532980	-5.1270570	1.0357530
N	-0.0209560	-6.2002710	1.3943050
C	1.7557370	-3.1025220	0.7719720
N	2.8279570	-3.4051570	1.0945150
C	-4.5455150	-1.4044190	-1.3486480
H	-4.1378250	-0.4505880	-1.651005

4. Copies of ^{1}H NMR, ^{13}C{^{1}H} NMR spectra and reports of HRMS of the compounds

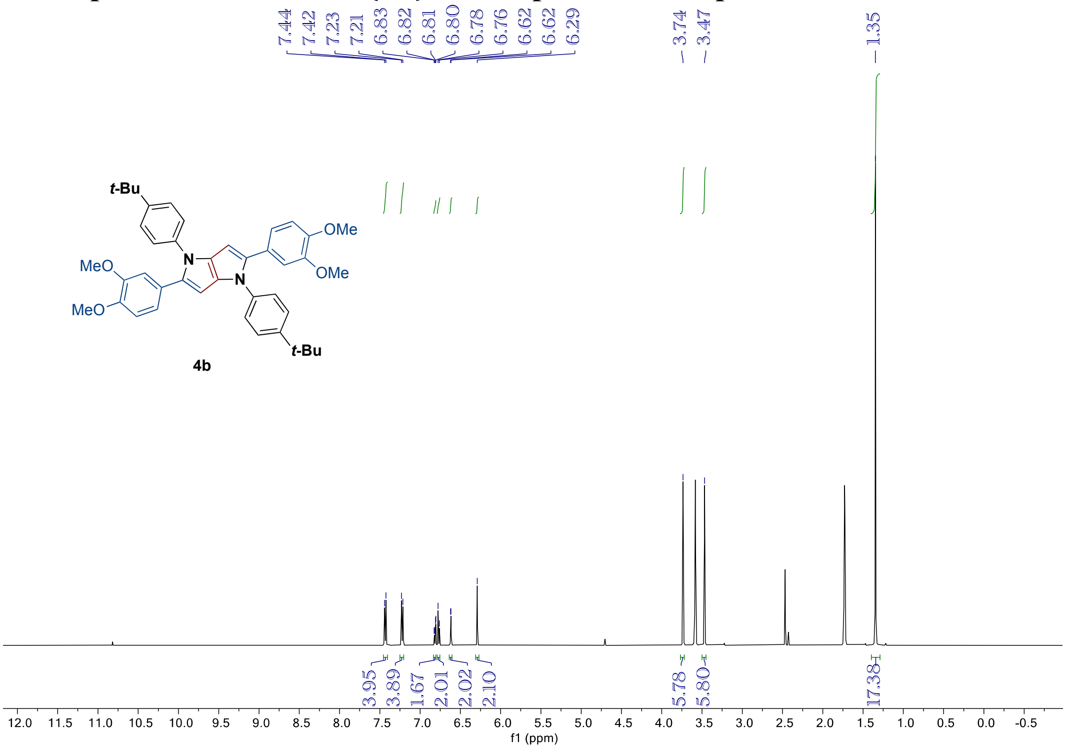

Figure S11. ^{1}H NMR (500 MHz, THF-d_8) spectrum of compound **4b**

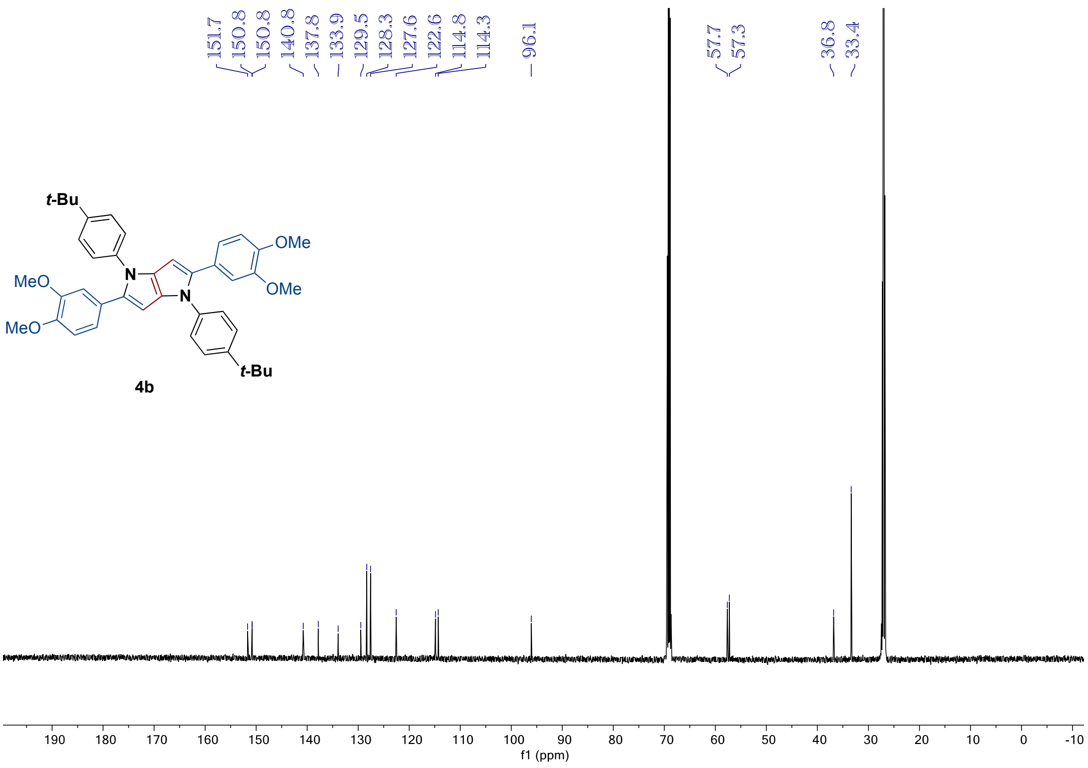

Figure S12. ^{13}C {^{1}H} NMR (126 MHz, THF-d_8) spectrum of compound **4b**

Single Mass Analysis
Tolerance = 3.0 mDa / DBE: min = -1.5, max = 300.0
Element prediction: Off
Number of isotope peaks used for i-FIT = 3
Monoisotopic Mass, Even Electron Ions
66 formula(e) evaluated with 1 results within limits (up to 50 closest results for each mass)
Elements Used:
C: 0-120 H: 0-200 N: 1-2 O: 1-4

Mass	Calc. Mass	mDa	PPM	DBE	Formula	i-FIT	i-FIT Norm	Fit Conf %	C	H	N	O
643.3530	643.3536	-0.6	-0.9	20.5	C42 H47 N2 O4	788.6	n/a	n/a	42	47	2	4

Figure S13. Report of HRMS of **4b**

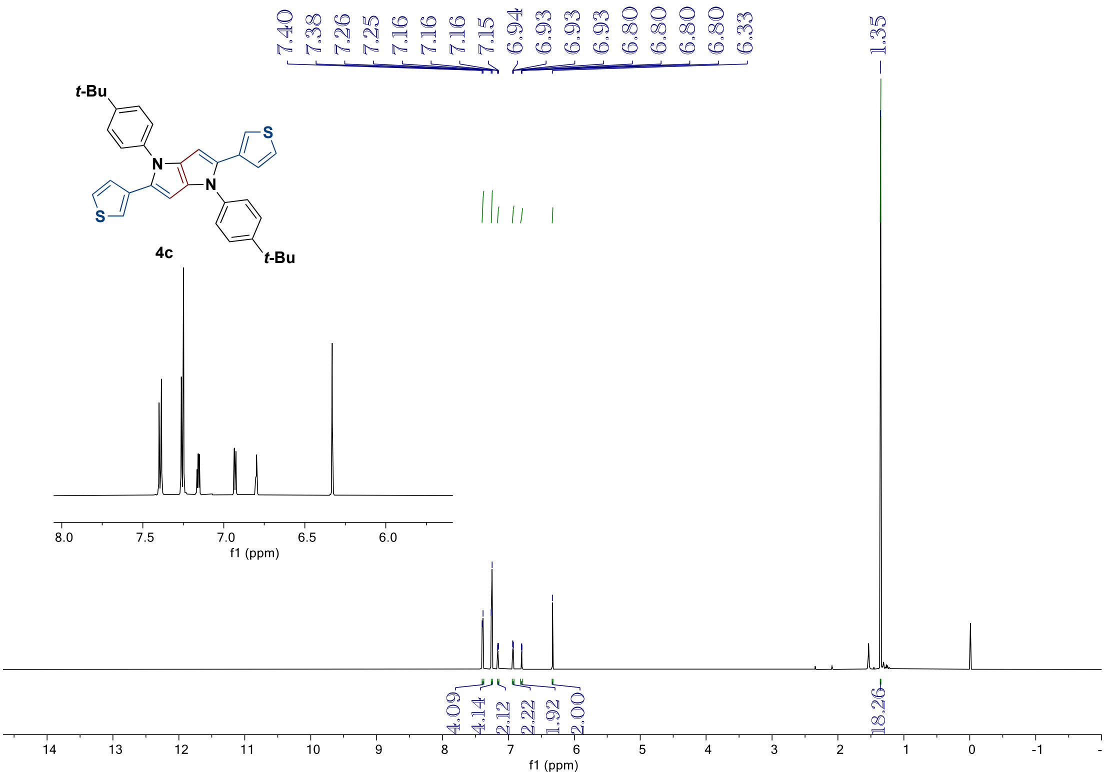

Figure S14. ^{1}H NMR (600 MHz, $CDCl_3$) spectrum of compound **4c**

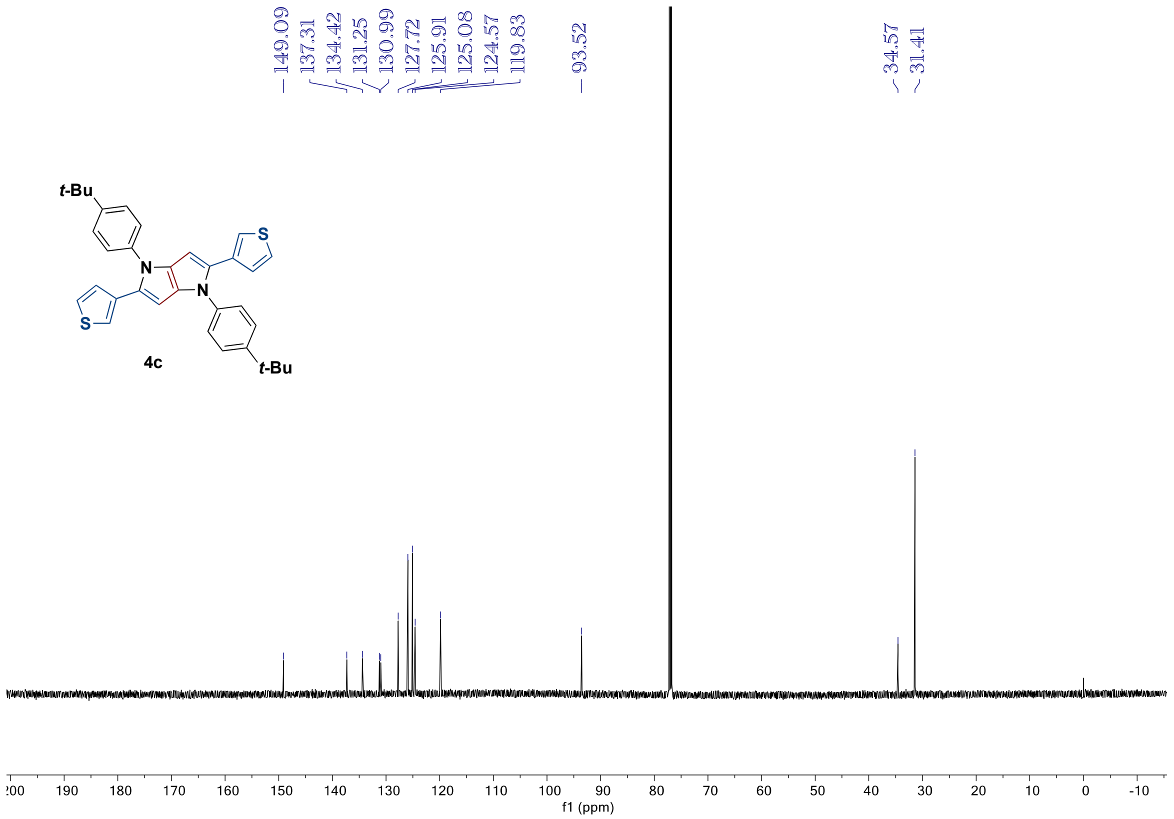

Figure S15. ^{13}C {^{1}H} NMR (151 MHz, $CDCl_3$) spectrum of compound **4c**

Single Mass Analysis
Tolerance = 3.0 mDa / DBE: min = -1.5, max = 300.0
Element prediction: Off
Number of isotope peaks used for i-FIT = 3
Monoisotopic Mass, Even Electron Ions
14 formula(e) evaluated with 1 results within limits (up to 50 closest results for each mass)
Elements Used:
C: 0-120 H: 0-200 N: 2-2 S: 1-2

Mass	Calc. Mass	mDa	PPM	DBE	Formula	i-FIT	i-FIT Norm	Fit Conf %	C	H	N	S
535.2247	535.2242	0.5	0.9	18.5	C34 H35 N2 S2	741.2	n/a	n/a	34	35	2	2

Figure S16. Report of HRMS of **4c**

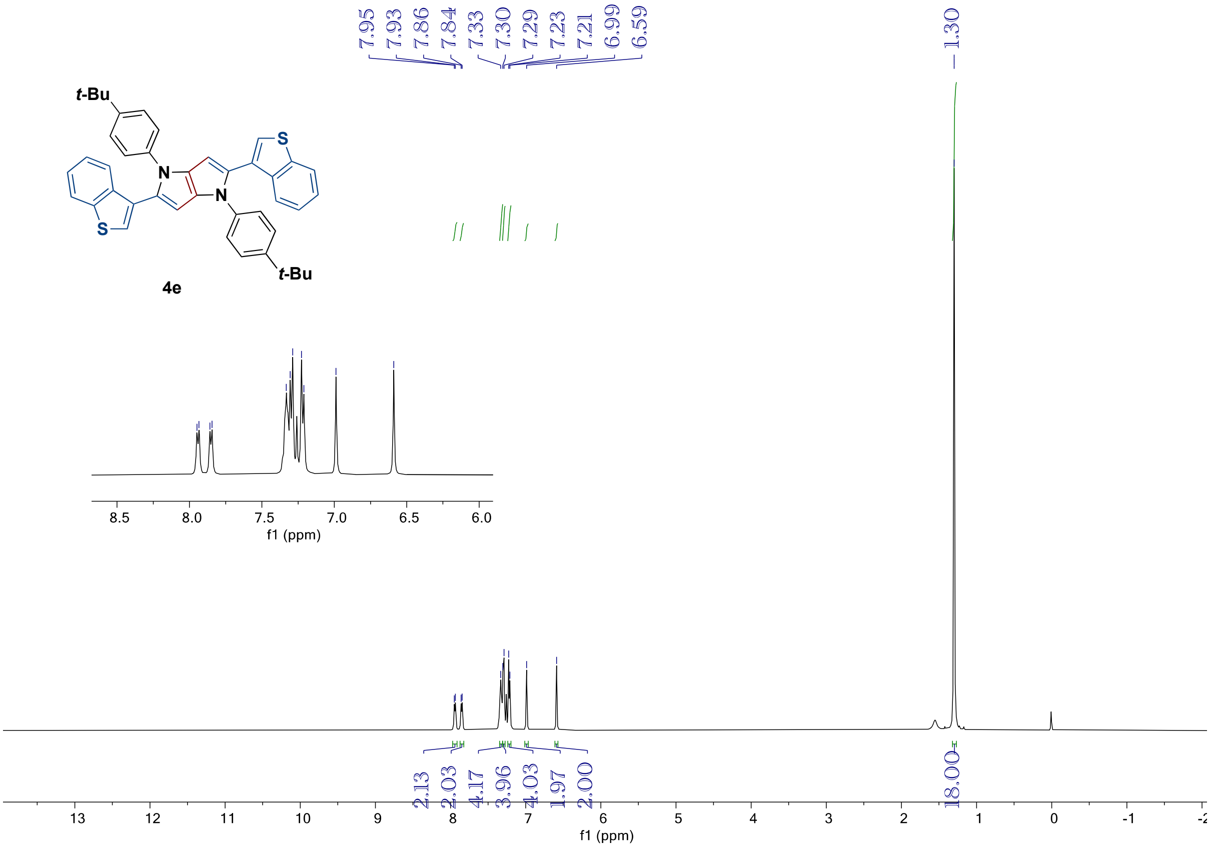

Figure S17. ^{1}H NMR (500 MHz, $CDCl_3$) spectrum of compound **4e**

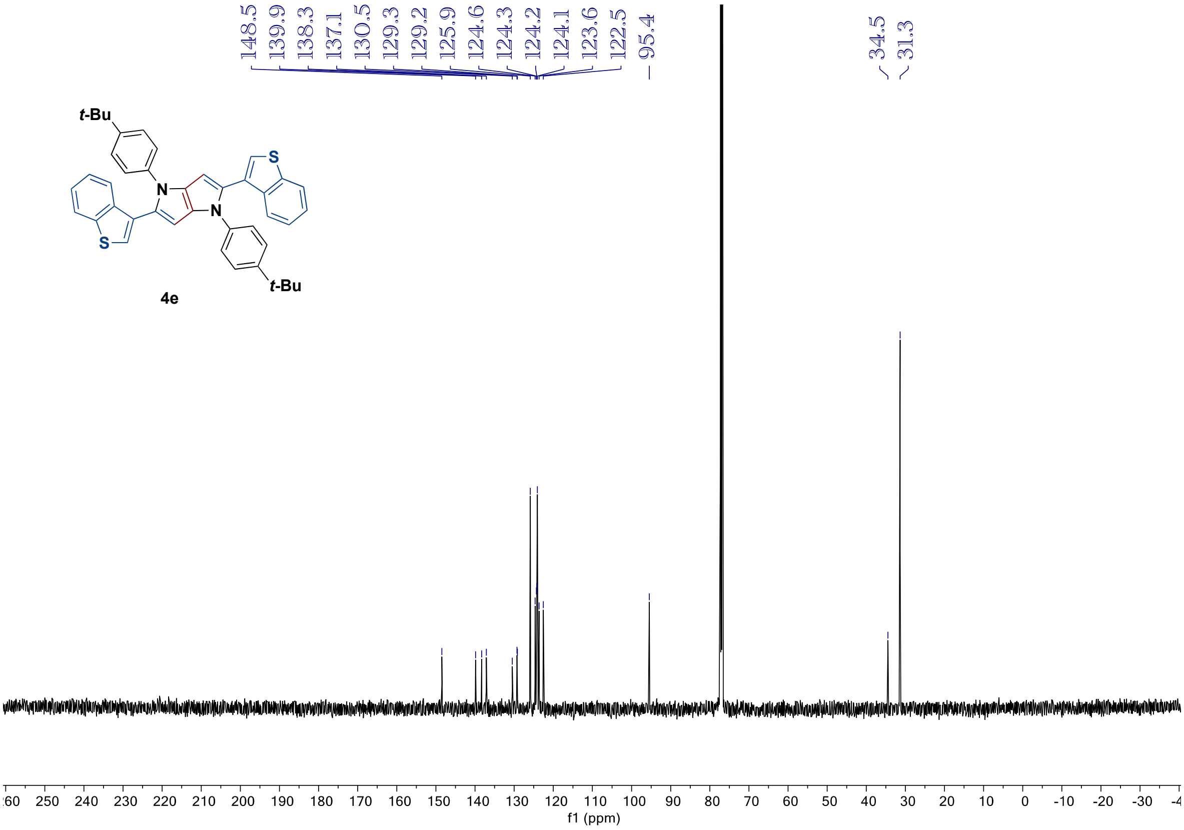

Figure S18. ^{13}C {^{1}H} NMR (126 MHz, $CDCl_3$) spectrum of compound **4e**

Single Mass Analysis
Tolerance = 3.0 mDa / DBE: min = -1.5, max = 300.0
Element prediction: Off
Number of isotope peaks used for i-FIT = 3
Monoisotopic Mass, Even Electron Ions
16 formula(e) evaluated with 1 results within limits (up to 50 closest results for each mass)
Elements Used:
C: 0-120 H: 0-200 N: 2-2 S: 1-2

Mass	Calc. Mass	mDa	PPM	DBE	Formula	i-FIT	i-FIT Norm	Fit Conf %	C	H	N	S
635.2552	635.2555	-0.3	-0.5	24.5	C42 H39 N2 S2	580.6	n/a	n/a	42	39	2	2

Figure S19. Report of HRMS of **4e**

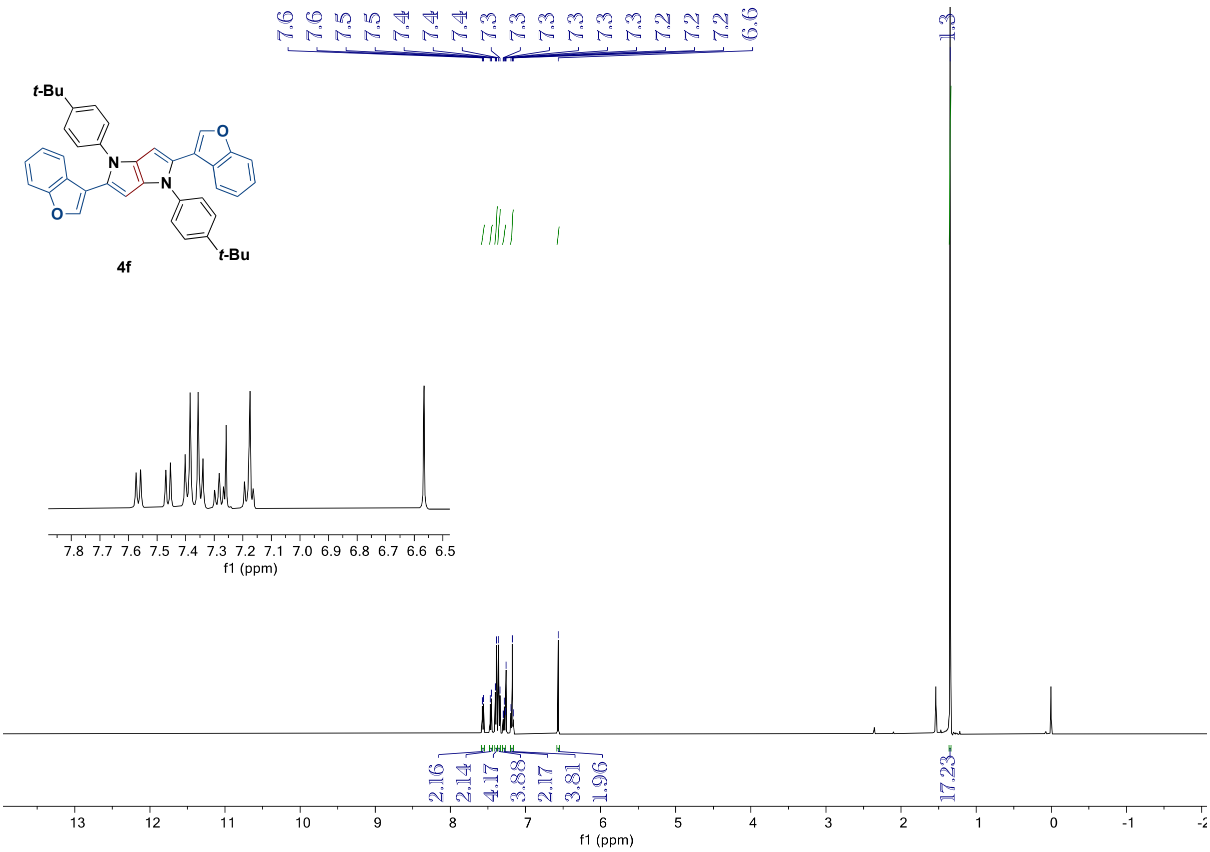

Figure S20. ^{1}H NMR (500 MHz, $CDCl_3$) spectrum of compound **4f**

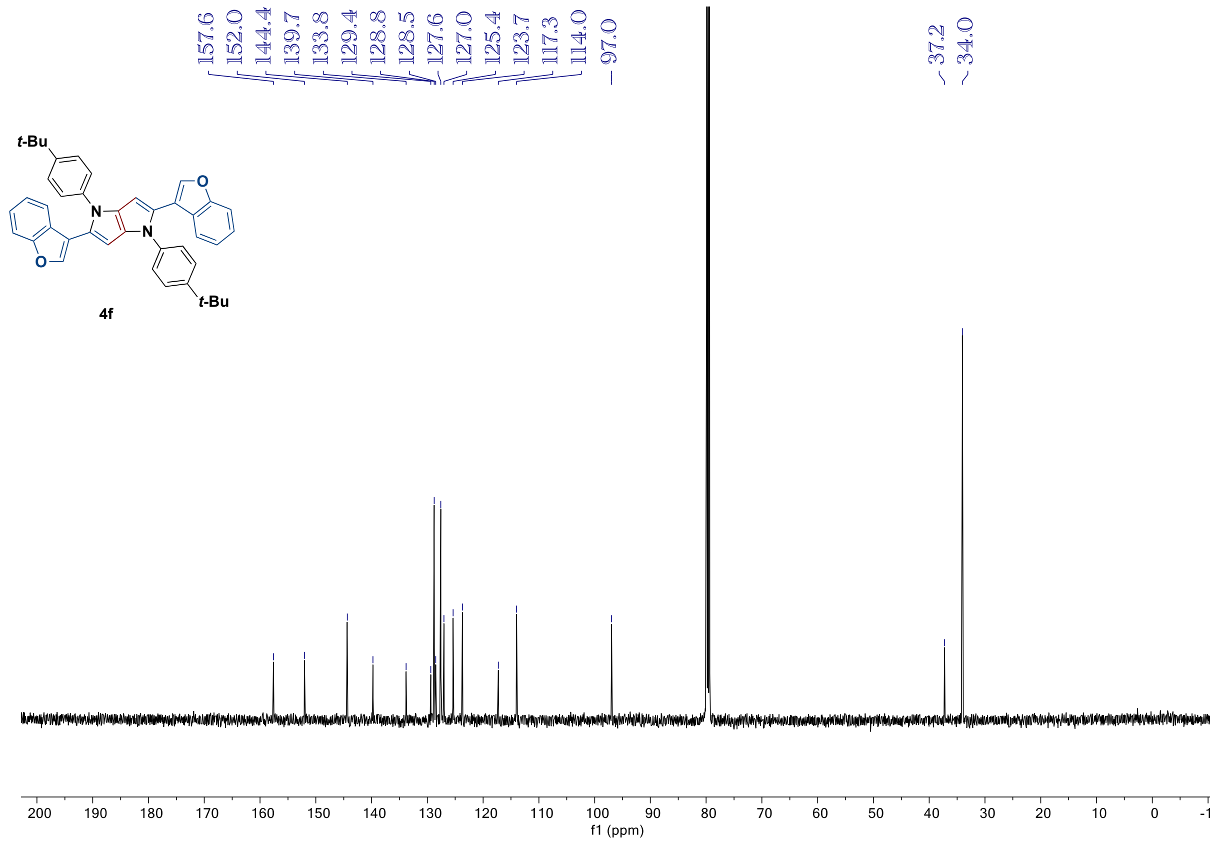

Figure S21. ^{13}C {^{1}H} NMR (126 MHz, $CDCl_3$) spectrum of compound **4f**

Single Mass Analysis
Tolerance = 3.0 mDa / DBE: min = -1.5, max = 300.0
Element prediction: Off
Number of isotope peaks used for i-FIT = 3
Monoisotopic Mass, Even Electron Ions
64 formula(e) evaluated with 1 results within limits (up to 50 closest results for each mass)
Elements Used:
C: 0-120 H: 0-200 N: 1-4 O: 1-2

Mass	Calc. Mass	mDa	PPM	DBE	Formula	i-FIT	i-FIT Norm	Fit Conf %	C	H	N	O
603.3015	603.3012	0.3	0.5	24.5	C42 H39 N2 O2	540.7	n/a	n/a	42	39	2	2

Figure S22. Report of HRMS of **4f**

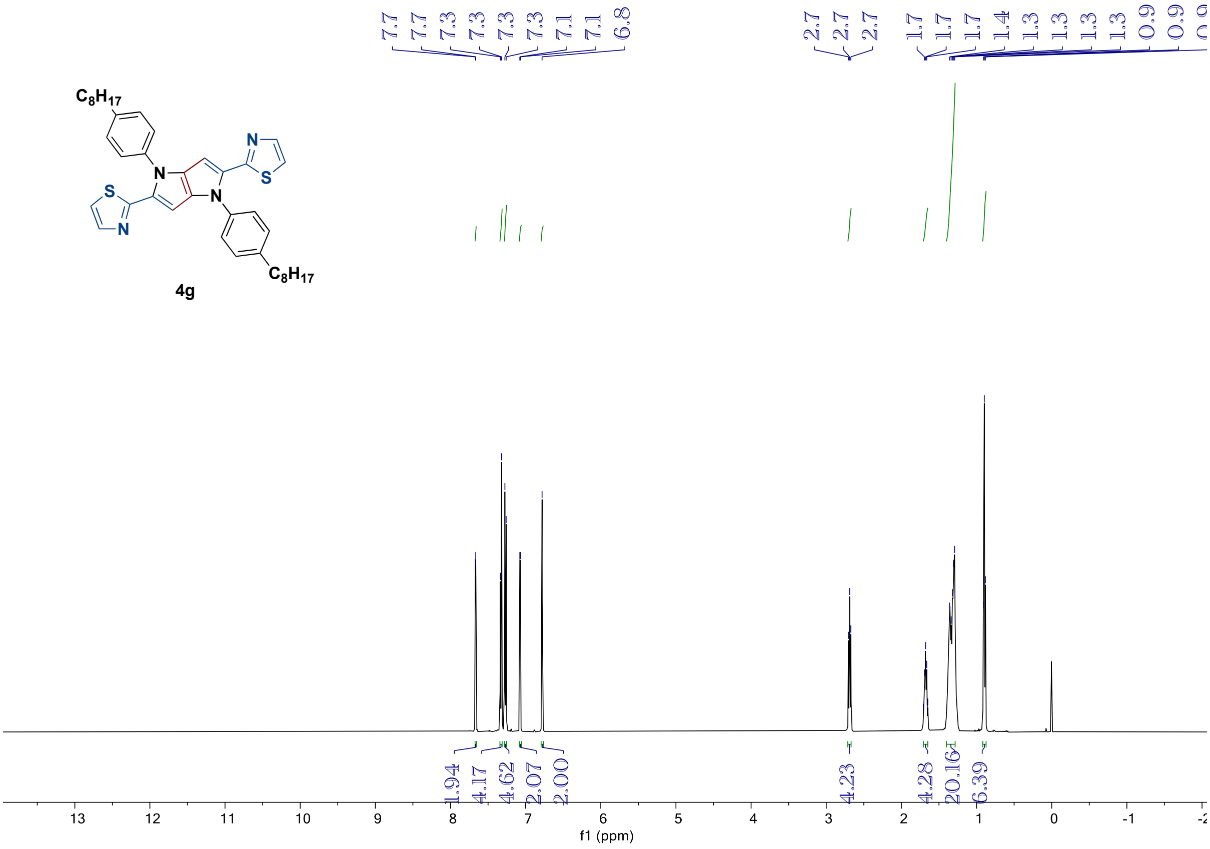

Figure S23. ^{1}H NMR (500 MHz, $CDCl_3$) spectrum of compound **4g**

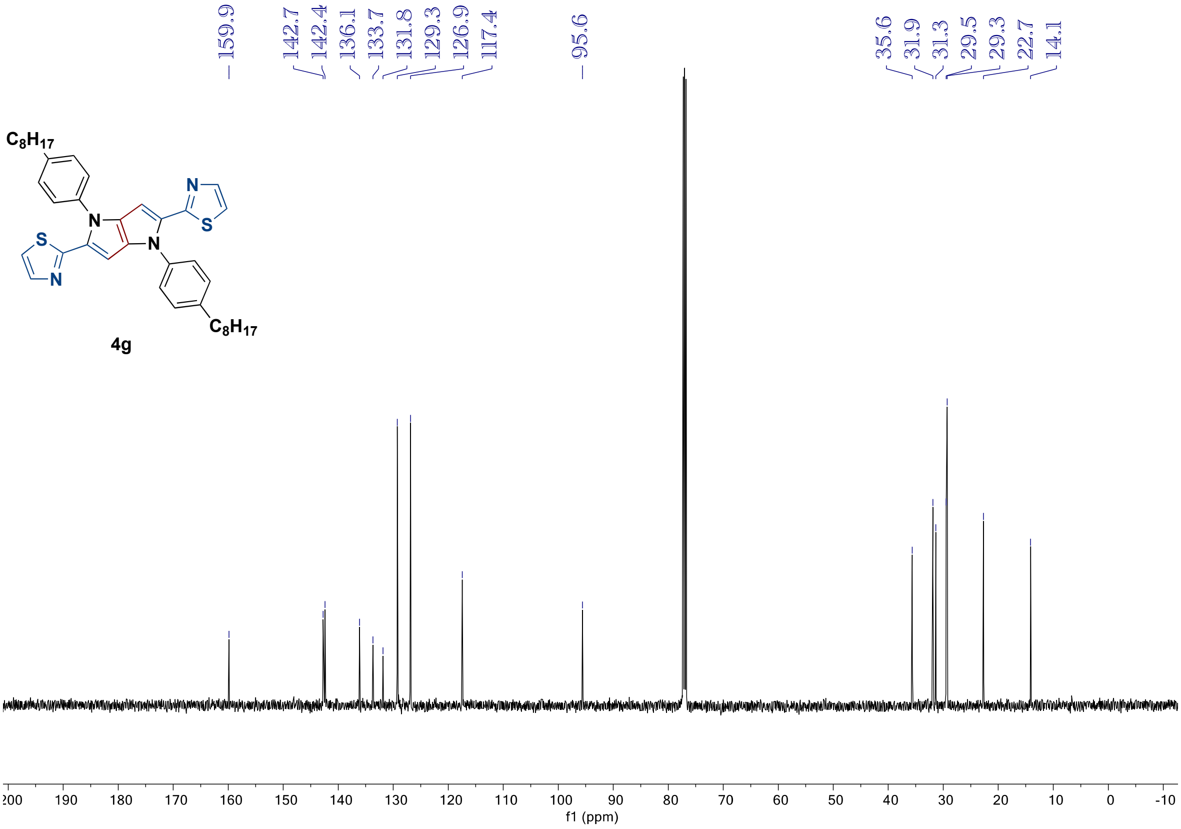

Figure S24. $^{13}C\ \{^{1}H\}$ NMR (600 MHz, $CDCl_3$) spectrum of compound **4g**

Single Mass Analysis
Tolerance = 5.0 mDa / DBE: min = -1.5, max = 300.0
Element prediction: Off
Number of isotope peaks used for i-FIT = 3
Monoisotopic Mass, Even Electron Ions
47 formula(e) evaluated with 1 results within limits (up to 50 closest results for each mass)
Elements Used:
C: 0-120 H: 0-200 N: 1-6 S: 2-2

Mass	Calc. Mass	mDa	PPM	DBE	Formula	i-FIT	i-FIT Norm	Fit Conf %	C	H	N	S
649.3403	649.3399	0.4	0.6	18.5	C40 H49 N4 S2	748.0	n/a	n/a	40	49	4	2

Figure S25. Report of HRMS of **4g**

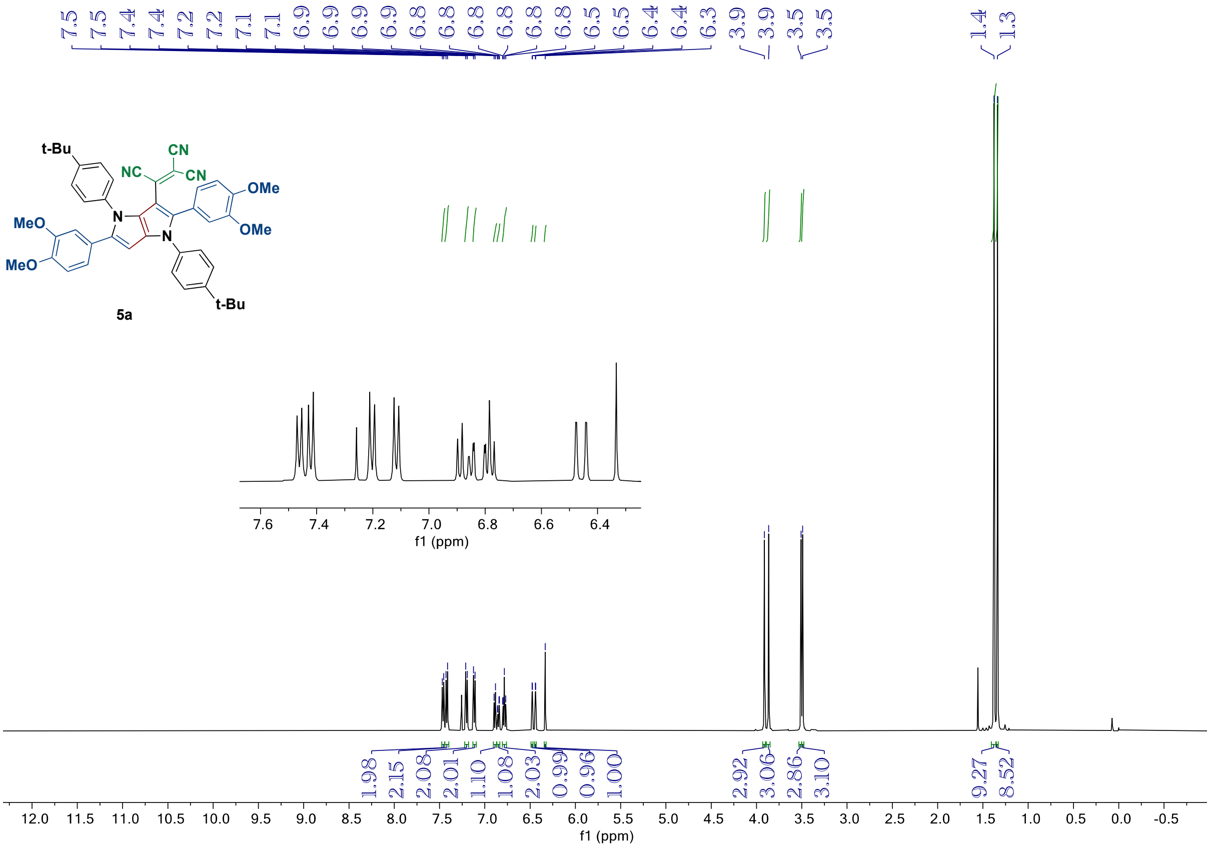

Figure S26. ^{1}H NMR (500 MHz, $CDCl_3$) spectrum of compound **5a**

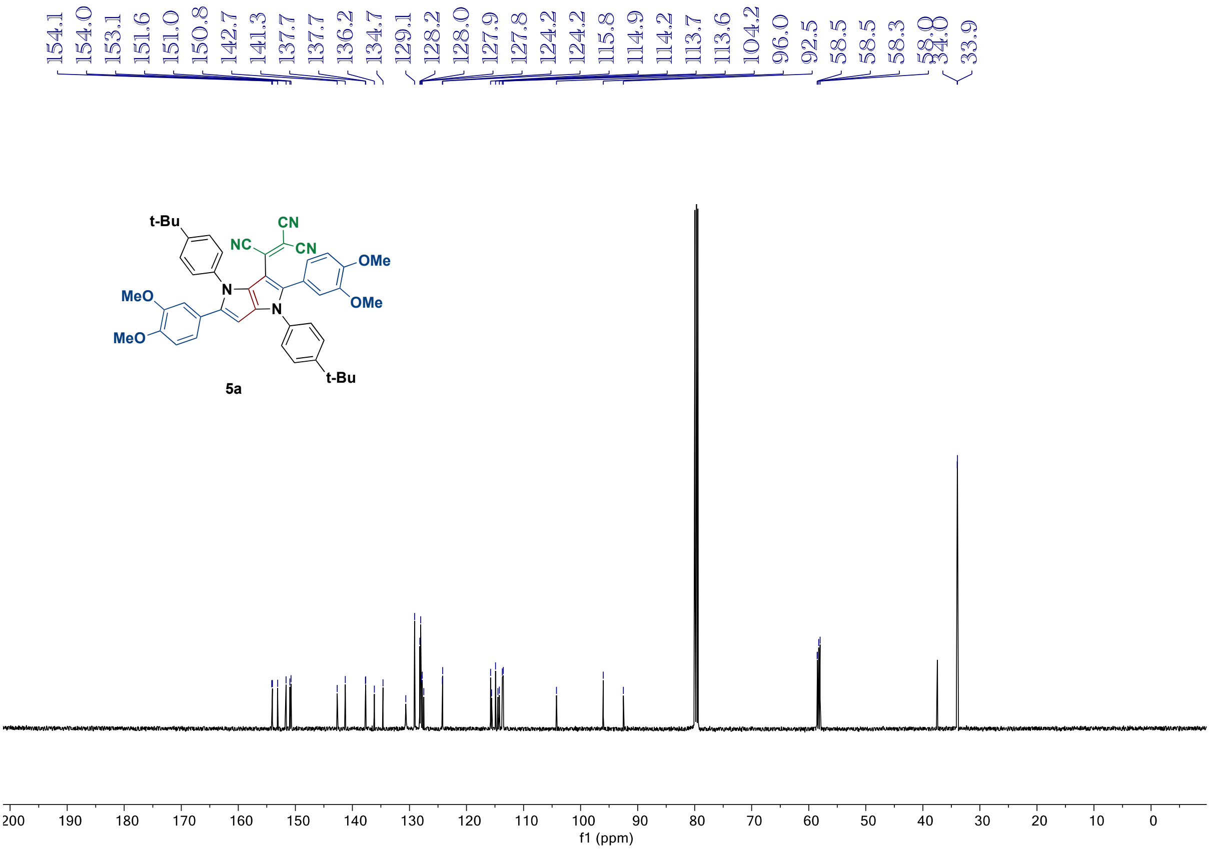

Figure S27. ^{13}C {^{1}H} NMR (126 MHz, $CDCl_3$) spectrum of compound **5a**

Single Mass Analysis
Tolerance = 3.0 mDa / DBE: min = -1.5, max = 300.0
Element prediction: Off
Number of isotope peaks used for i-FIT = 3
Monoisotopic Mass, Even Electron Ions
147 formula(e) evaluated with 1 results within limits (up to 50 closest results for each mass)
Elements Used:

Mass	Calc. Mass	mDa	PPM	DBE	Formula	i-FIT	i-FIT Norm	Fit Conf %	C	H	N	O
744.3554	744.3550	0.4	0.5	27.5	C47 H46 N5 O4	814.6	n/a	n/a	47	46	5	4

Figure S28. Report of HRMS of **5a**

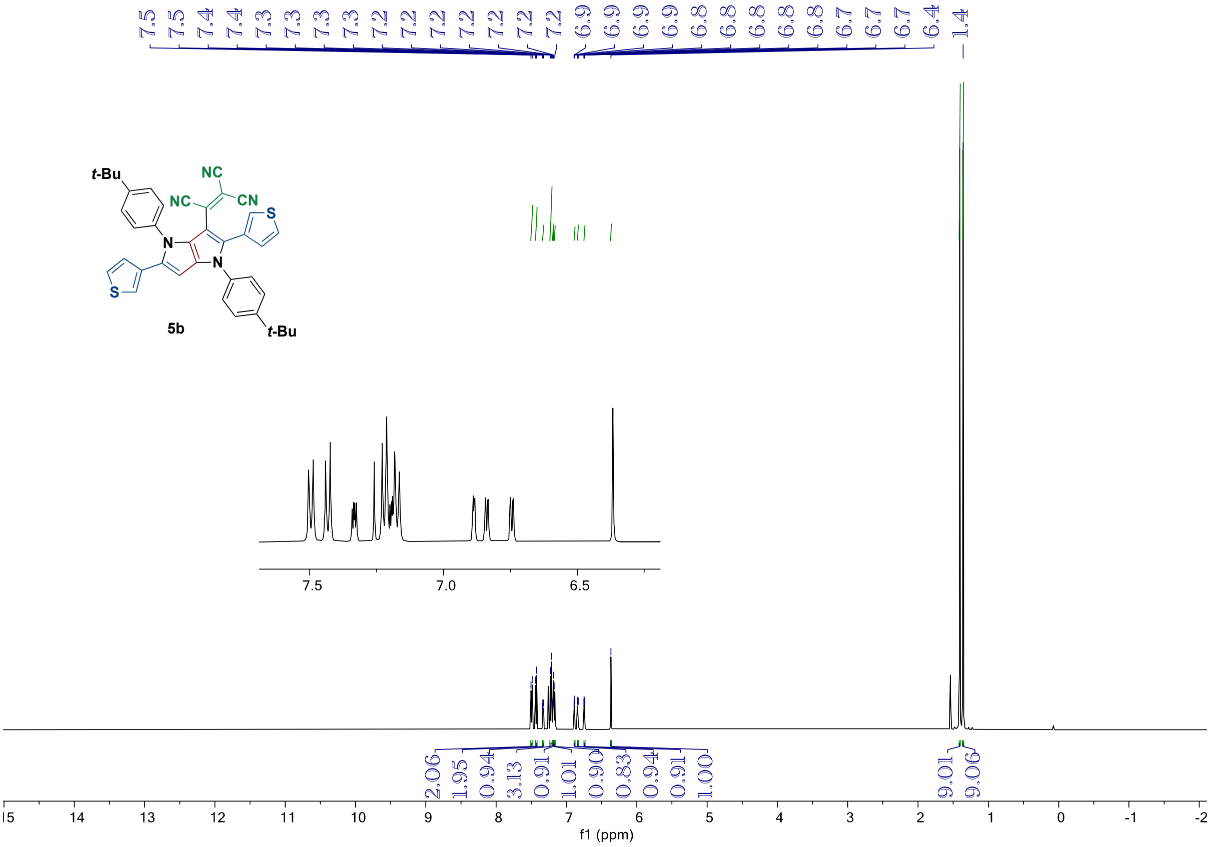

Figure S29. ^{1}H NMR (500 MHz, $CDCl_3$) spectrum of compound **5b**

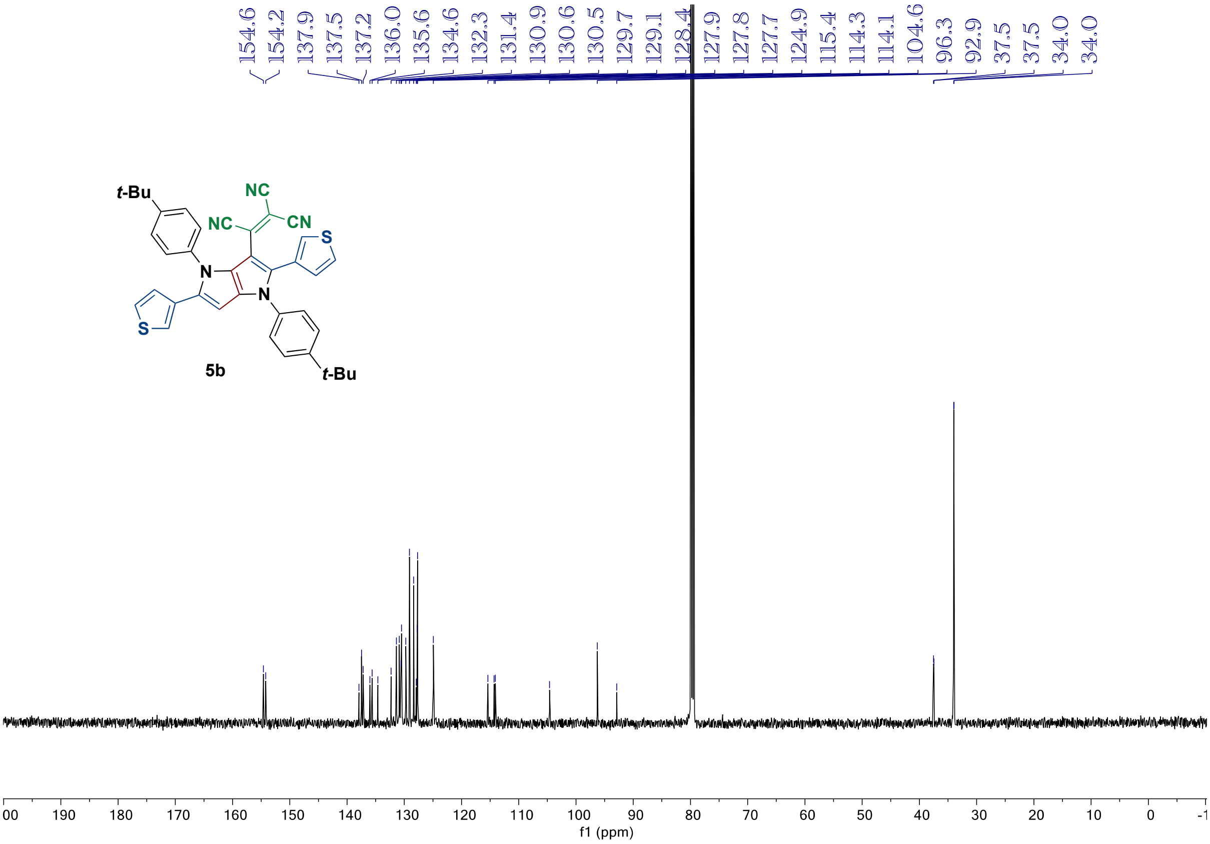

Figure S30. ^{13}C {^{1}H} NMR (126 MHz, $CDCl_3$) spectrum of compound **5b**

Single Mass Analysis
Tolerance = 5.0 mDa / DBE: min = -1.5, max = 300.0
Element prediction: Off
Number of isotope peaks used for i-FIT = 3
Monoisotopic Mass, Even Electron Ions
46 formula(e) evaluated with 1 results within limits (up to 50 closest results for each mass)
Elements Used:
C: 0-120 H: 0-200 N: 1-6 S: 2-2

Mass	Calc. Mass	mDa	PPM	DBE	Formula	i-FIT	i-FIT Norm	Fit Conf %	C	H	N	S
636.2258	636.2256	0.2	0.3	25.5	C39 H34 N5 S2	761.3	n/a	n/a	39	34	5	2

Figure S31. Report of HRMS of **5b**

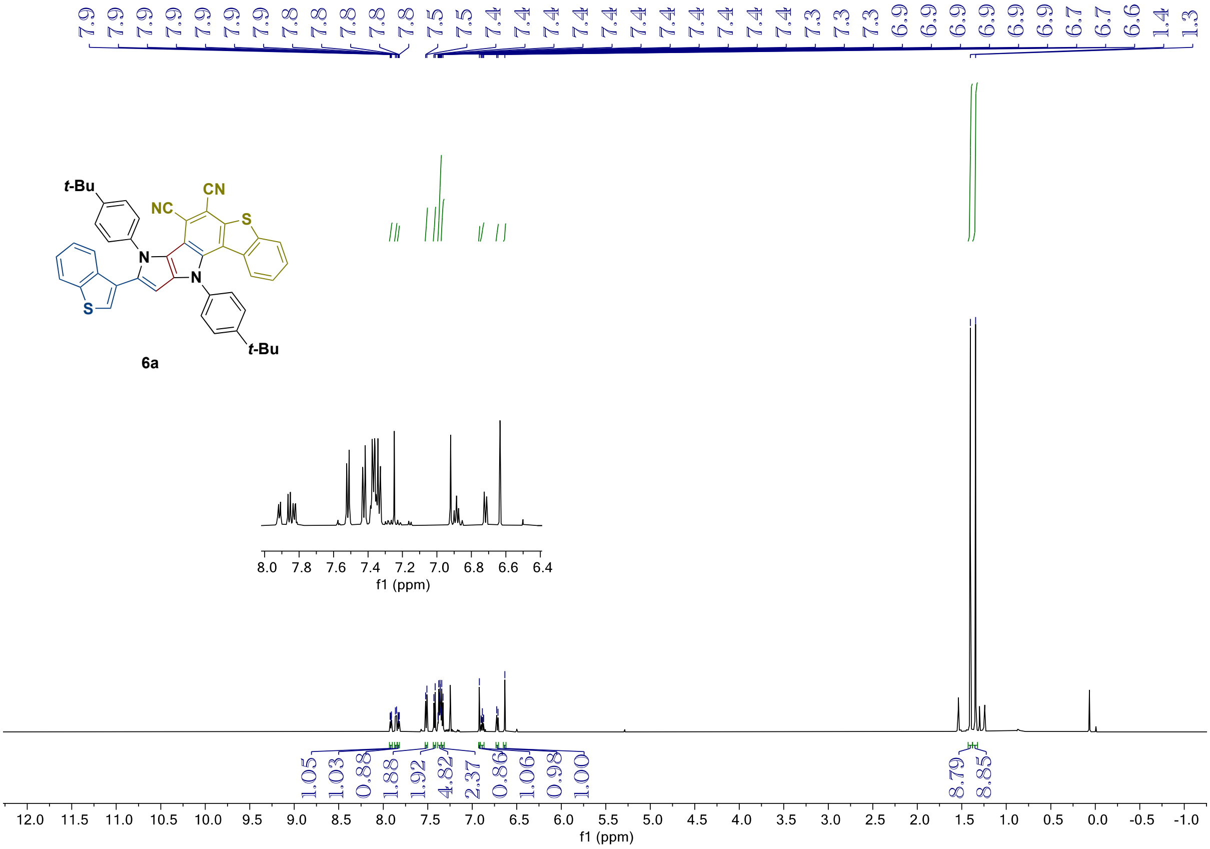

Figure S32. ^{1}H NMR (600 MHz, $CDCl_3$) spectrum of compound **6a**

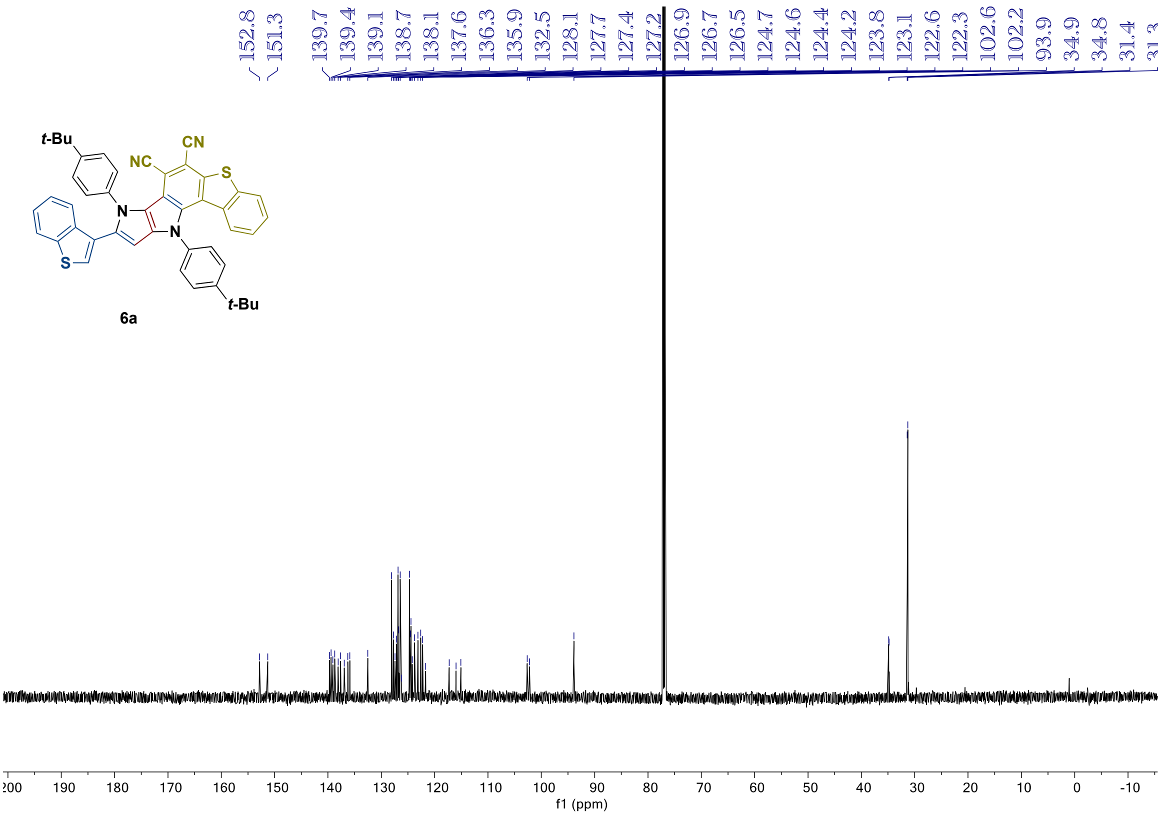

Figure S33. $^{13}C\ \{^{1}H\}$ NMR (151 MHz, $CDCl_3$) spectrum of compound **6a**

Single Mass Analysis
Tolerance = 3.0 mDa / DBE: min = -1.5, max = 500.0
Element prediction: Off
Number of isotope peaks used for i-FIT = 3
Monoisotopic Mass, Odd and Even Electron Ions
52 formula(e) evaluated with 1 results within limits (up to 50 closest results for each mass)
Elements Used:
C: 0-100 H: 0-200 N: 2-4 S: 1-2

Mass	Calc. Mass	mDa	PPM	DBE	Formula	i-FIT	i-FIT Norm	Fit Conf %	C	H	N	S
708.2387	708.2381	0.6	0.8	31.0	C46 H36 N4 S2	903.9	n/a	n/a	46	36	4	2

Figure S34. Report of HRMS of **6a**

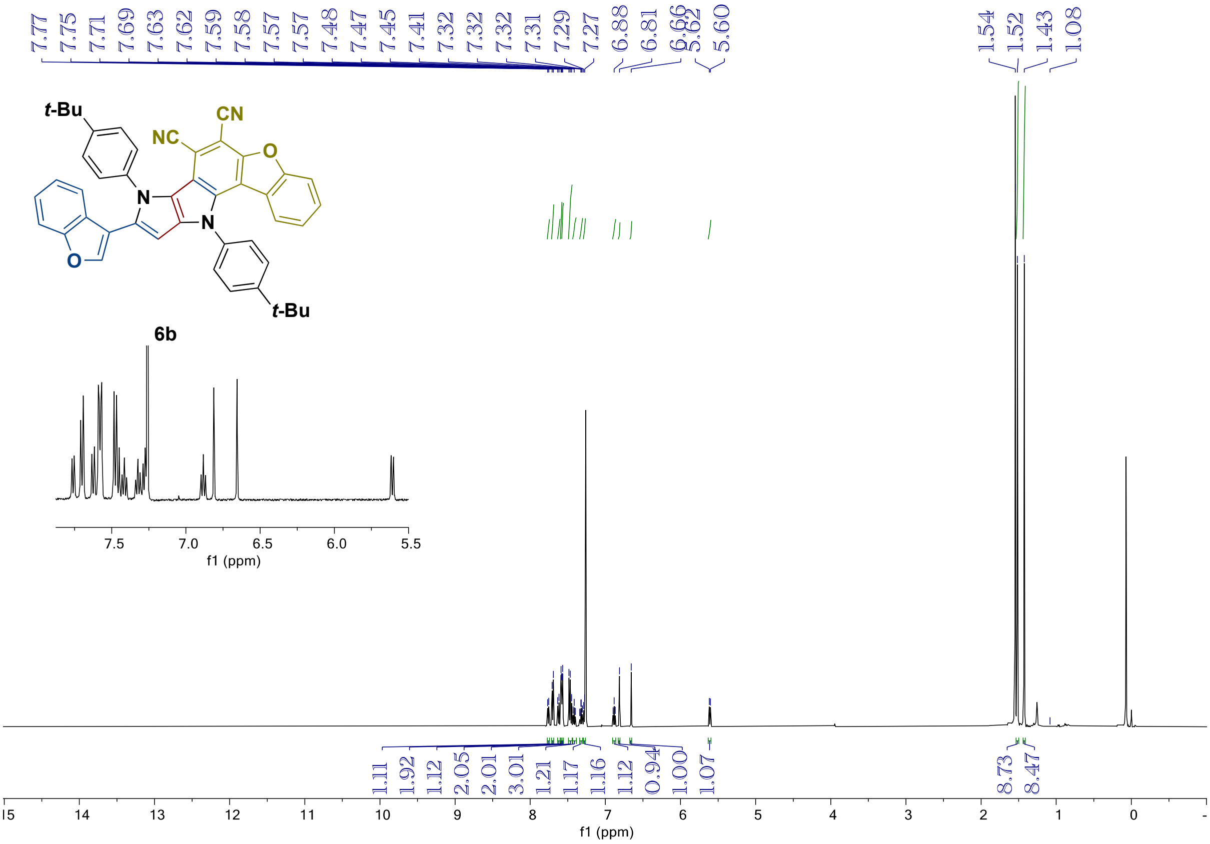

Figure S35. ^{1}H NMR (600 MHz, $CDCl_3$) spectrum of compound **6b**

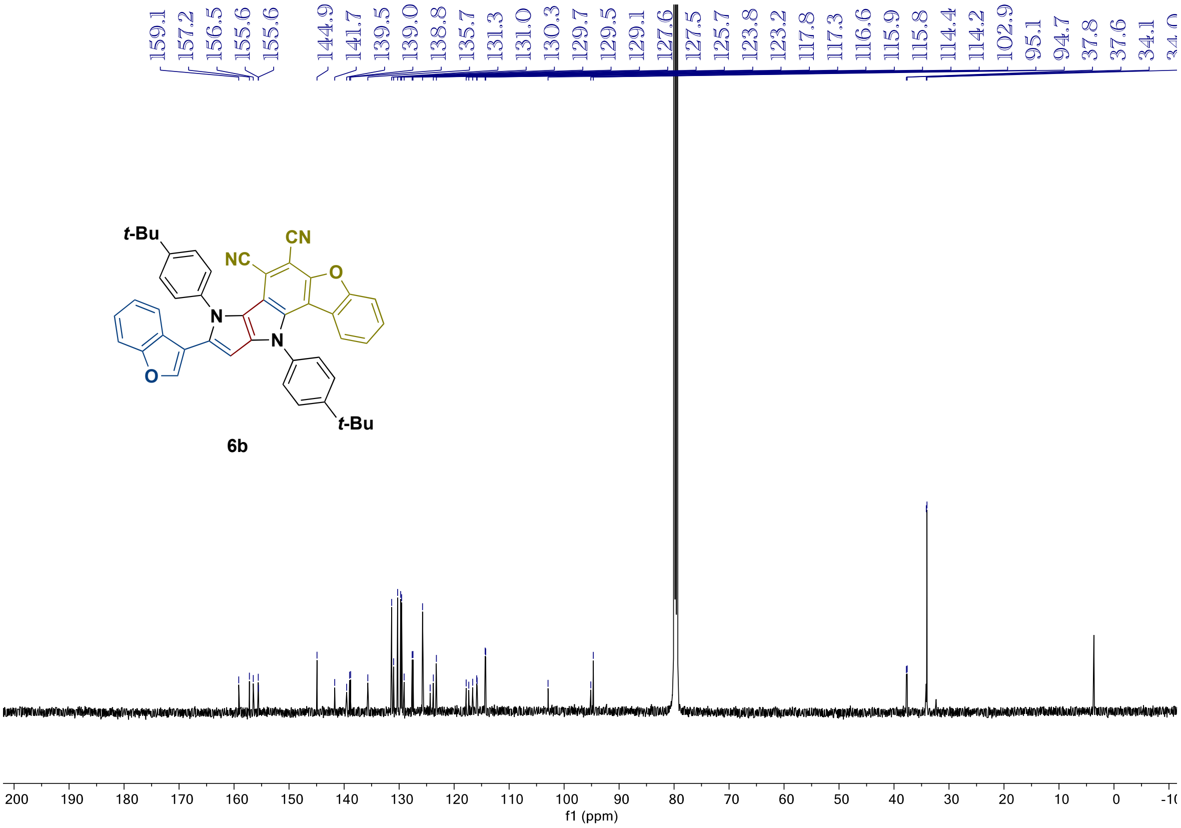

Figure S36. ^{13}C {^{1}H} NMR (126 MHz, $CDCl_3$) spectrum of compound **6b**

Single Mass Analysis
Tolerance = 5.0 mDa / DBE: min = -1.5, max = 100.0
Element prediction: Off
Number of isotope peaks used for i-FIT = 3
Monoisotopic Mass, Odd and Even Electron Ions
67 formula(e) evaluated with 1 results within limits (up to 50 closest results for each mass)
Elements Used:
C: 0-100 H: 0-200 N: 1-4 O: 1-2

Mass	Calc. Mass	mDa	PPM	DBE	Formula	i-FIT	i-FIT Norm	Fit Conf %	C	H	N	O
677.2919	677.2917	0.2	0.3	30.5	C46 H37 N4 O2	761.5	n/a	n/a	46	37	4	2

Figure S37. Report of HRMS of **6b**

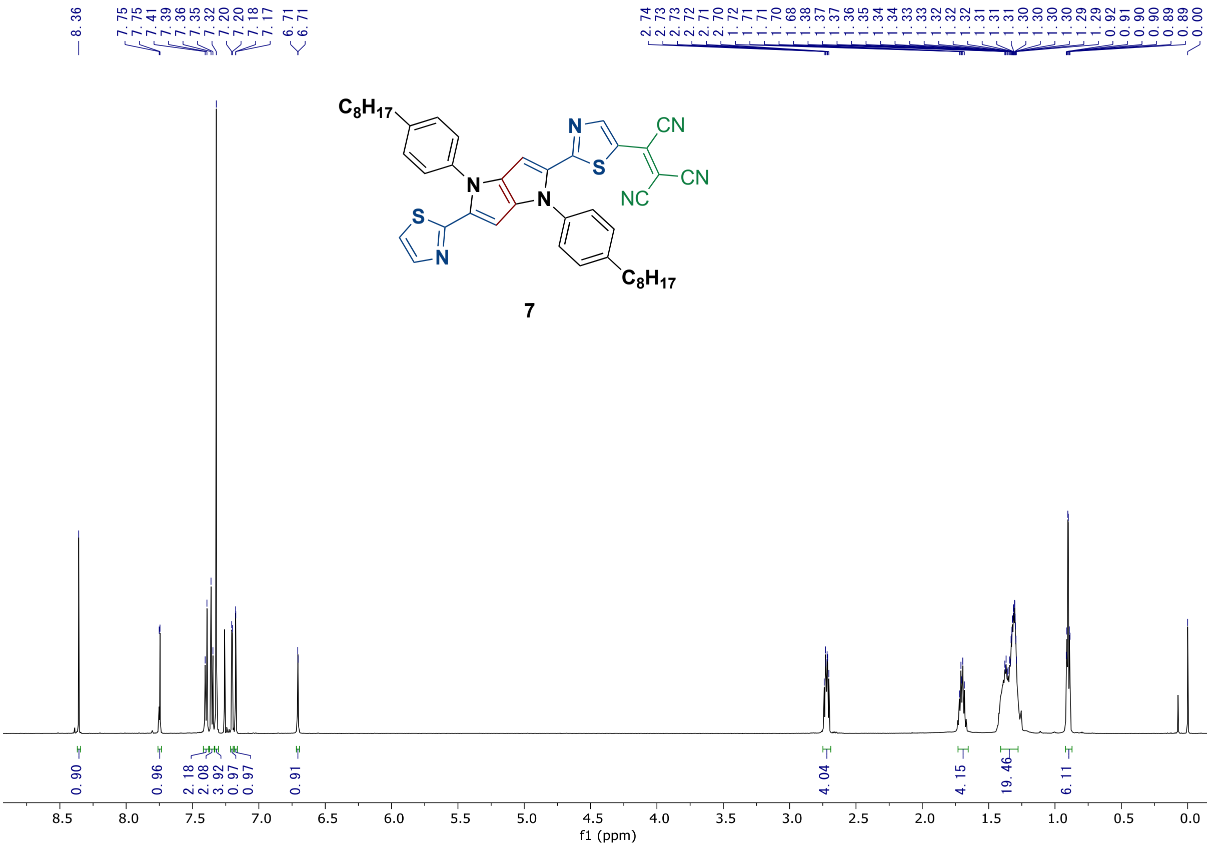

Figure S38. ^{1}H NMR (600 MHz, $CDCl_3$) spectrum of compound **7**

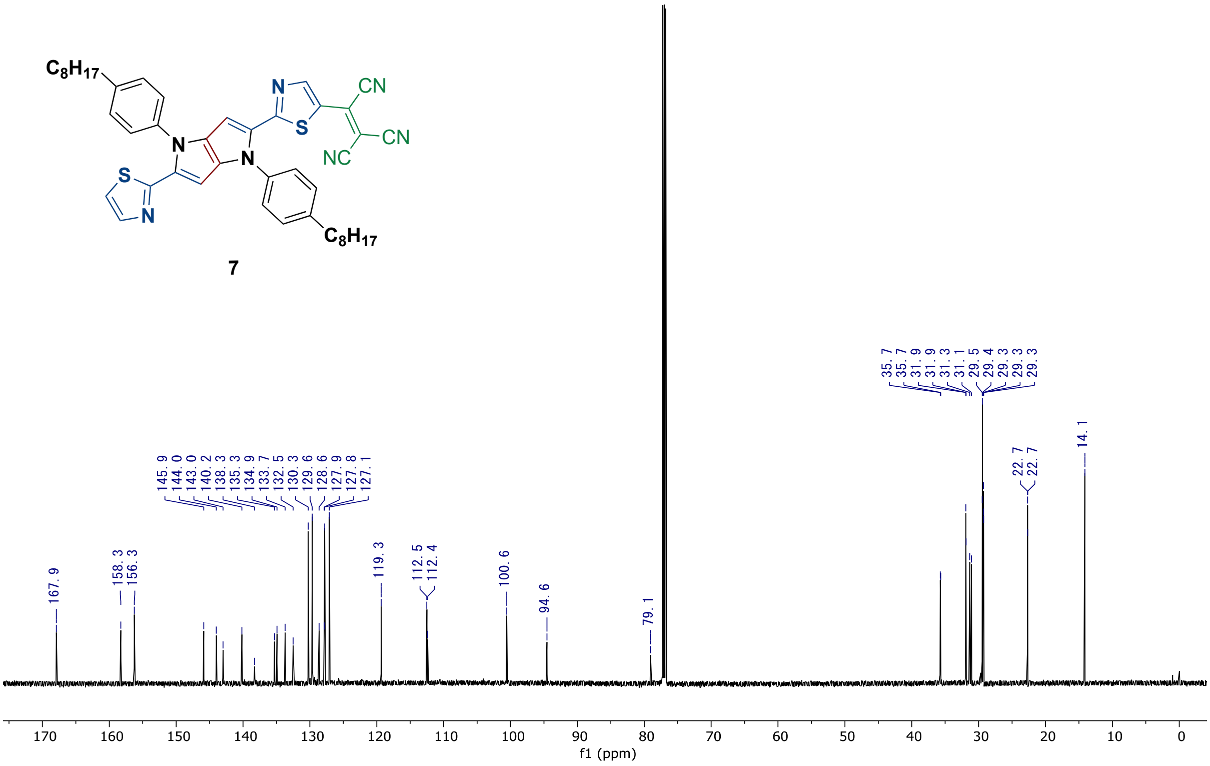

Figure S39. ^{13}C {^{1}H} NMR (151 MHz, $CDCl_3$) spectrum of compound **7**

Single Mass Analysis
Tolerance = 5.0 mDa / DBE: min = -1.5, max = 300.0
Element prediction: Off
Number of isotope peaks used for i-FIT = 3
Monoisotopic Mass, Even Electron Ions
54 formula(e) evaluated with 1 results within limits (up to 50 closest results for each mass)
Elements Used:
C: 0-120 H: 0-200 N: 2-7 S: 2-2

Mass	Calc. Mass	mDa	PPM	DBE	Formula	i-FIT	i-FIT Norm	Fit Conf %	C	H	N	S
750.3402	750.3413	-1.1	-1.5	25.5	C45 H48 N7 S2	609.2	n/a	n/a	45	48	7	2

Figure S40. Report of HRMS of **7**

5. Photophysical properties

Table S3. Photophysical properties of derivatives **4b**, **4c**, **4e**, **4f**, **4g** in toluene and **5a 5b**, **6a, 6b** and **7** obtained in toluene, DCM and DMSO

Comp.	Solvent	$\lambda_{max\,(Ab)}$ (nm)	ε @ λ_{max} ($M^{-1}cm^{-1}$)	$\lambda_{max\,(Em)}$ (nm)	Stokes Shift (cm^{-1})	Φ_{fl}
**4b**	Tol	353,300	40700, 26100	418	4400	0.64[a]
**4c**	Tol	350, 297	33300, 25400	402	3700	0.31[a]
**4e**	Tol	350, 305	23200, 17500	415	4500	0.17[a]
**4f**	Tol	335	20200	404	5100	0.22[a]
**4g**	Tol	394	42800	460	3600	0.46[b]
**5a**	Tol	630, 477, 335	3000, 6600, 36300	nd	nd	nd
	DCM	610, 482, 333	3400, 7500, 38500	nd	nd	nd
	DMSO	654, 481, 336	2900, 6000, 34800	nd	nd	nd
**5b**	Tol	590, 471, 328	2800, 6400, 33800,	nd	nd	nd
	DCM	604, 478, 329	3600, 7000, 32000,	nd	nd	nd
	DMSO	605, 475, 330	2500, 15700	nd	nd	nd
**6a**	Tol	436, 389, 331	6400, 9200, 40500	518	3600	0.08[b]
	DCM	443, 393, 330	6800, 10100, 46000	557	4600	0.11[b]
	DMSO	445,396, 329	2500, 3900, 17800	590	5500	0.05[b]
**6b**	Tol	429, 379, 315	5700, 1500, 37000	520	5400	0.45[b]
	DCM	440, 382, 313, 279	6100, 15100, 39300, 43400	557	4800	0.19[b]
	DMSO	443, 386, 313, 279	4300, 11900, 31600, 35500	587	5500	0.04[b]
**7**	Tol	643, 374	66700, 17200	nd	nd	nd
	DCM	660	62800	nd	nd	nd
	DMSO	639, 397	9100, 13300	nd	nd	nd

[a]Standard: Quinine sulphate in H_2SO_4 (0.5 M Φ_{fl} = 0.54); [b]Standard: Coumarin 143 in EtOH (Φ_{fl} = 0.54); nd: not detected.

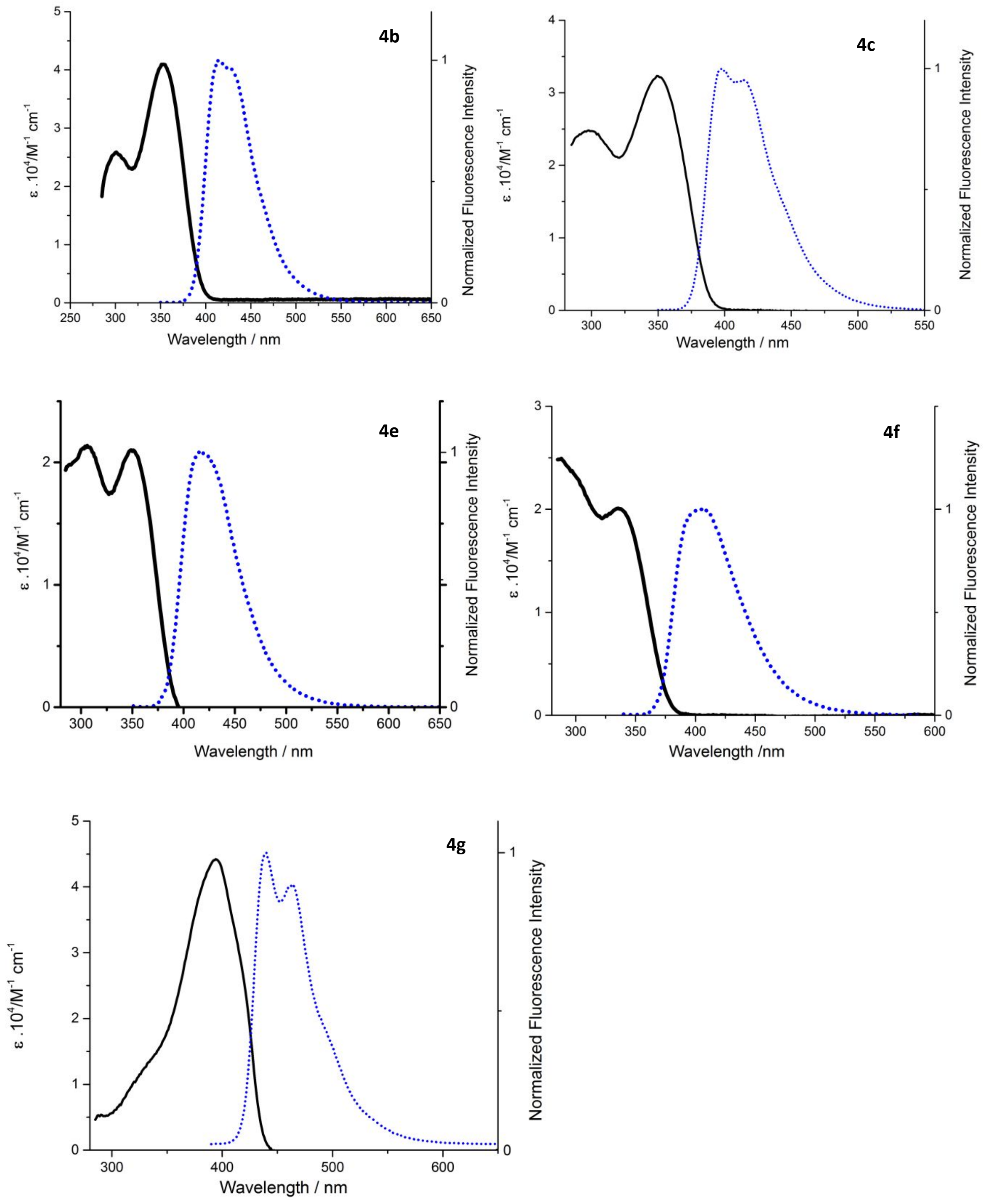

Figure S41. Molar extinction spectrum (solid black line) and normalized fluorescence emission spectrum (short-dotted blue line, excitation at 440 nm for **4b, 4c, 4e;** excitation at 330 nm for **4f** and excitation at 380 nm for **4g**) of derivatives **4b, 4c, 4e, 4f** and **4g** in toluene respectively.

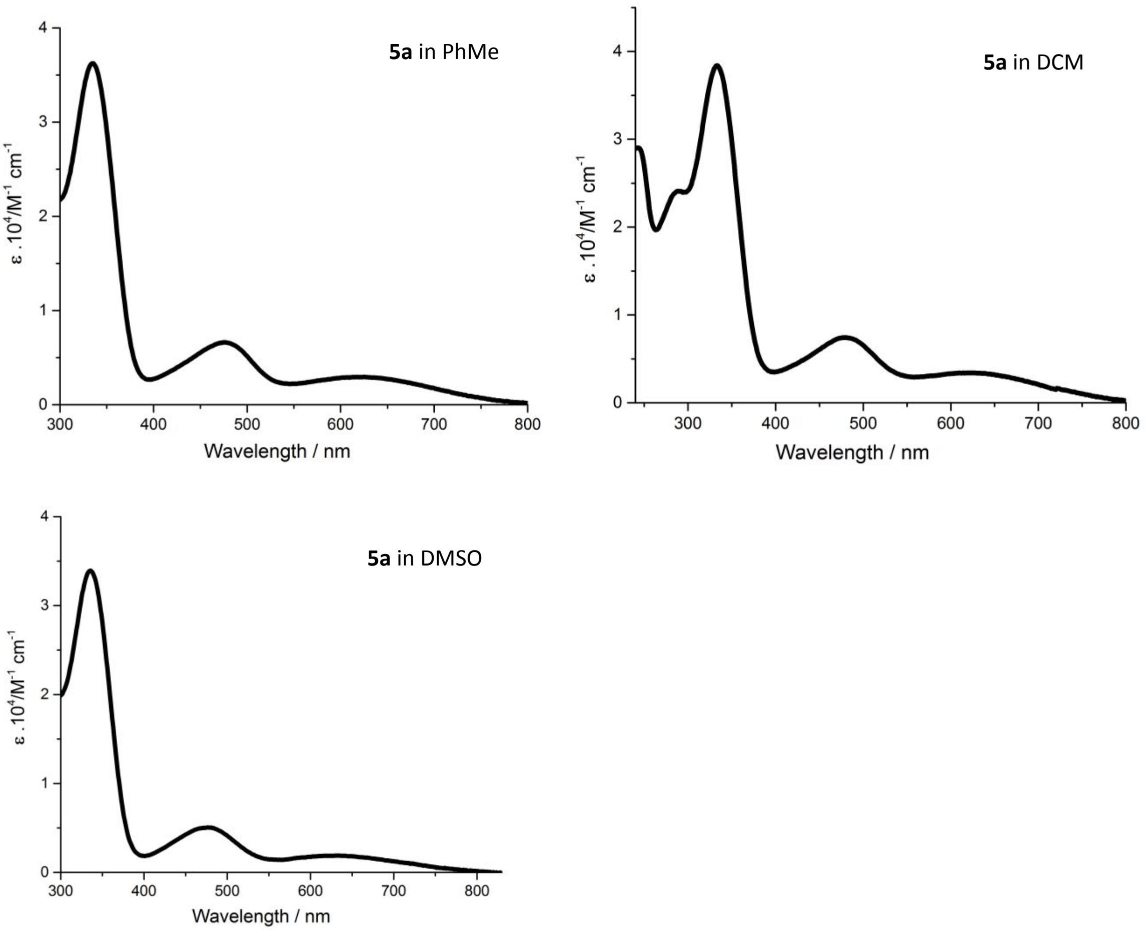

Figure S42. Molar extinction spectrum of **5a** in toluene, DCM and DMSO, respectively.

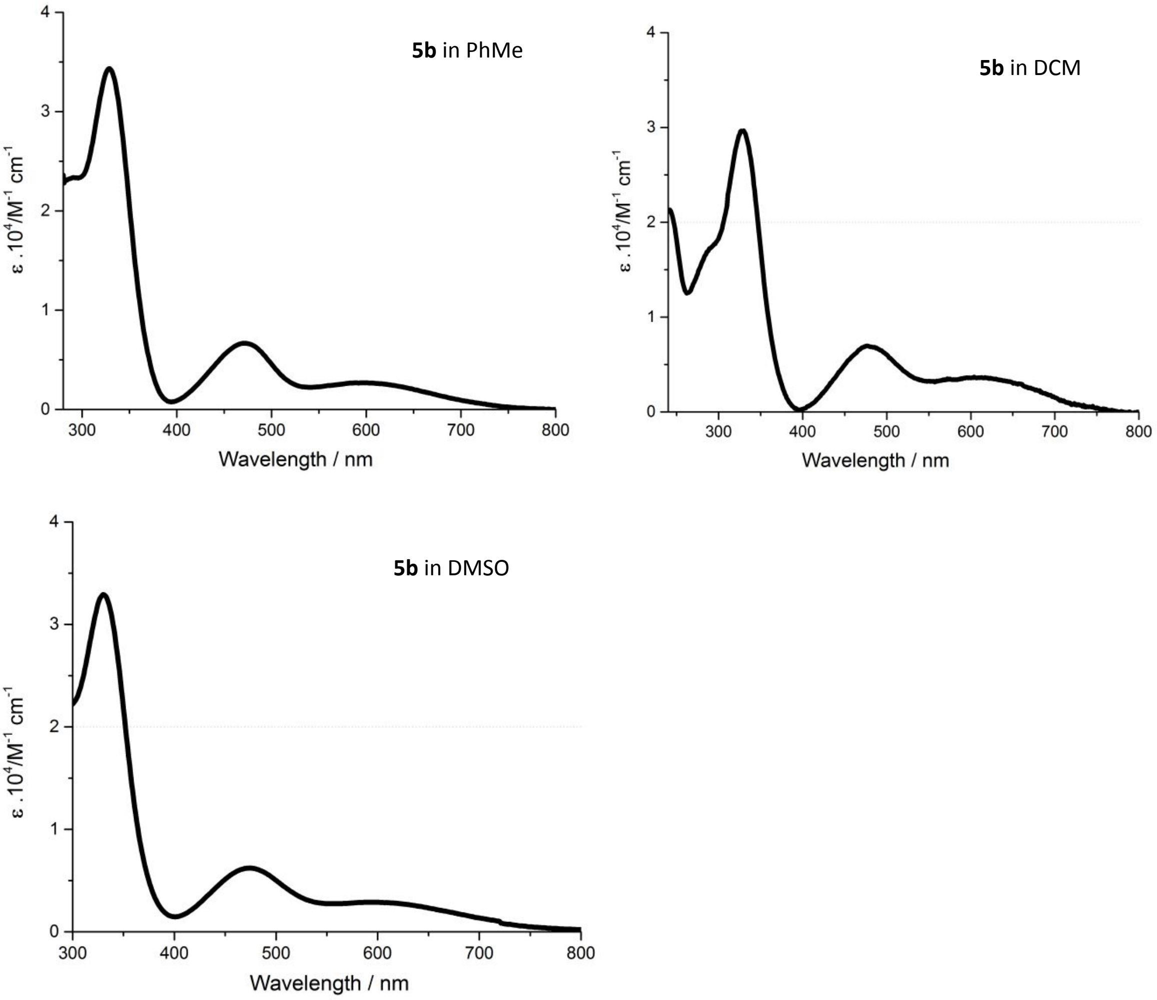

Figure S43. Molar extinction spectrum of **5b** in toluene, DCM and DMSO, respectively.

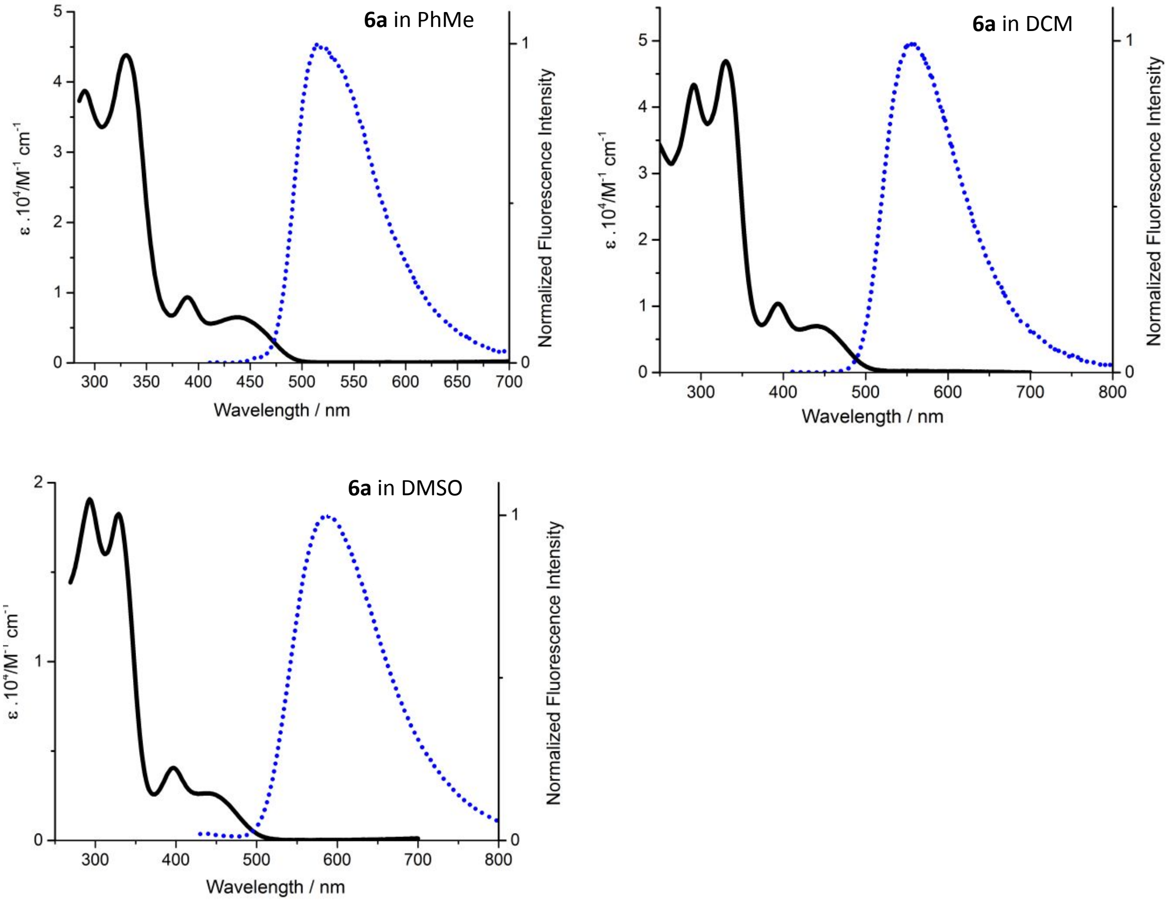

Figure S44. Molar extinction spectrum (solid black line) and normalized fluorescence emission spectrum (short-dotted blue line, excitation at 400 nm) of **6a** in toluene, DCM and DMSO, respectively.

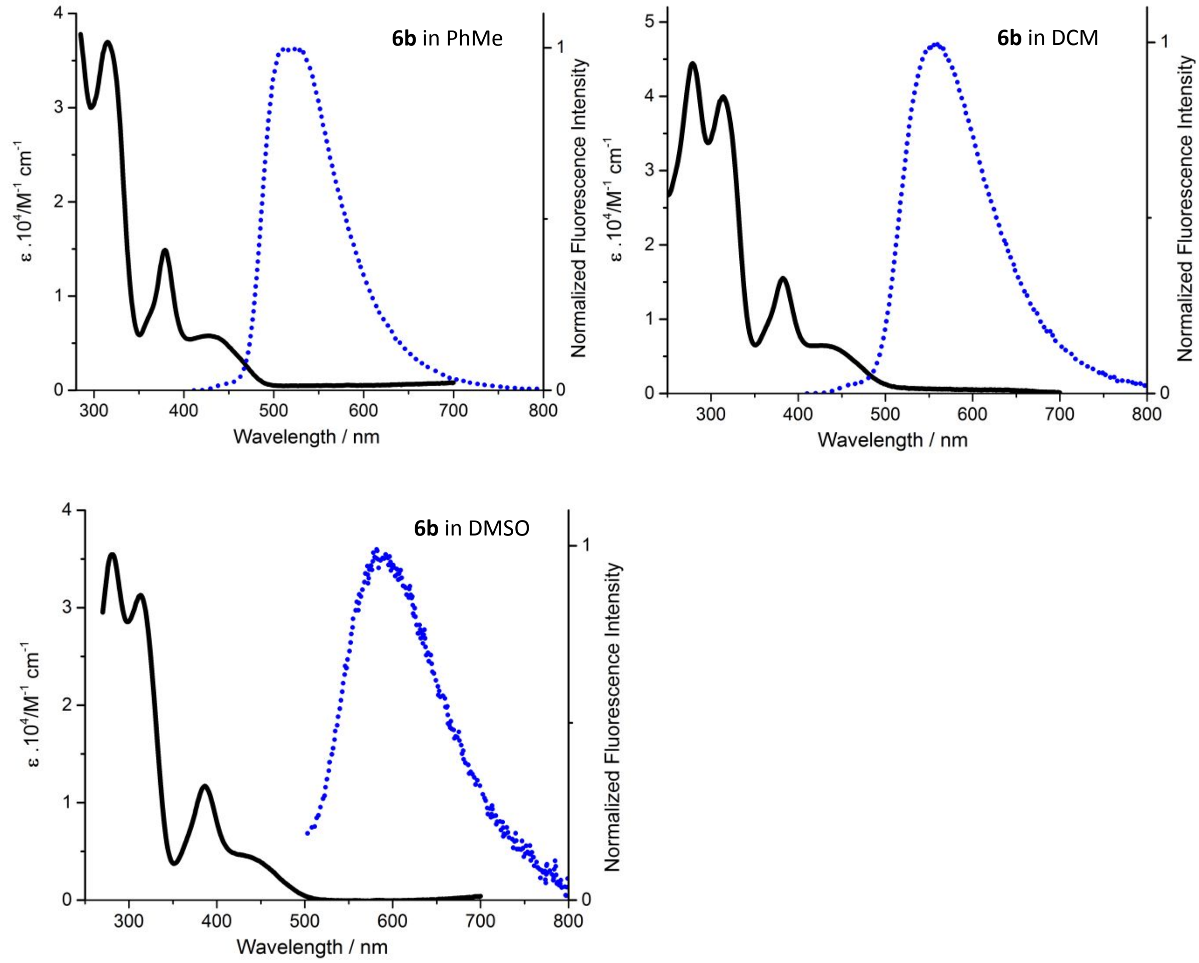

Figure S45. Molar extinction spectrum (solid black line) and normalized fluorescence emission spectrum (short-dotted blue line, excitation at 400 nm) of **6b** in toluene, DCM and DMSO, respectively.

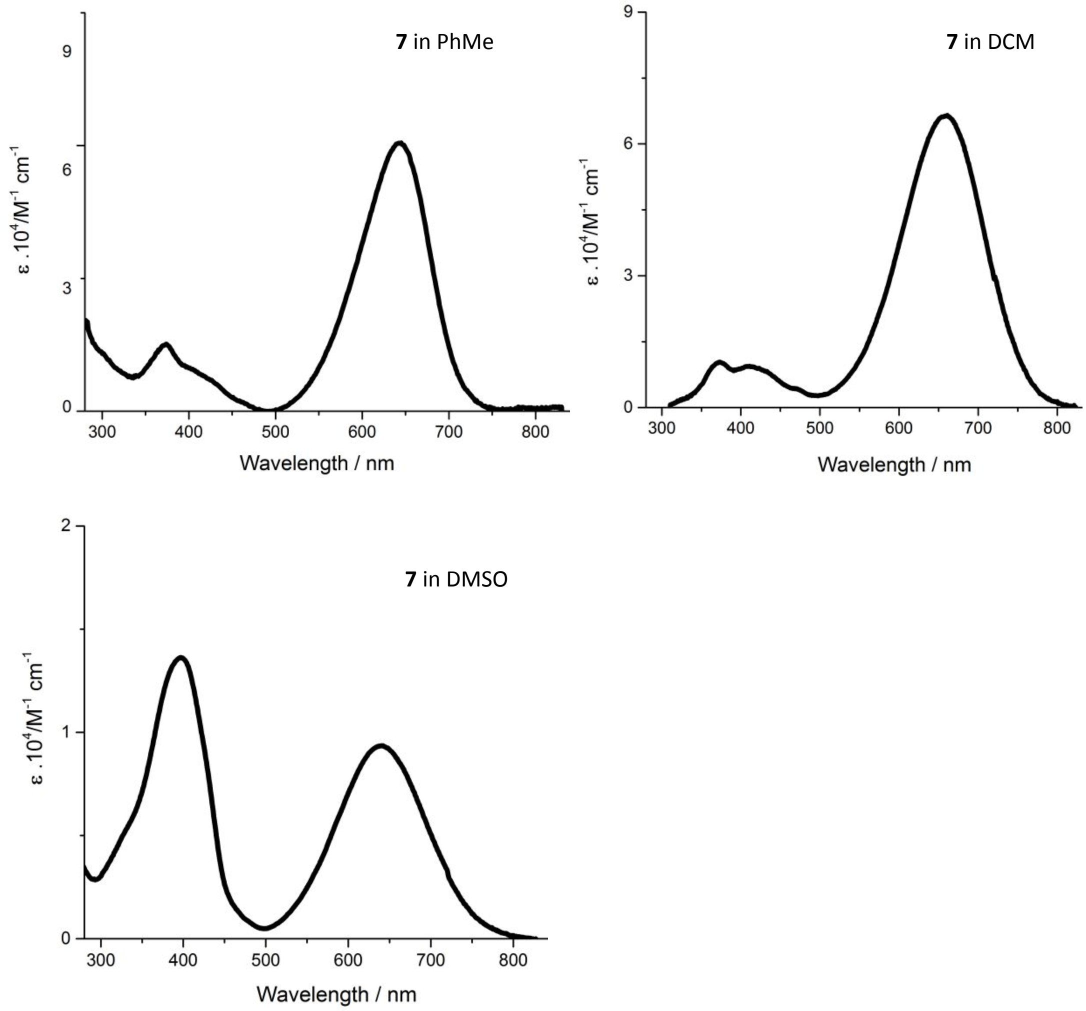

Figure S46. Molar extinction spectrum of **7** in toluene, DCM and DMSO, respectively.

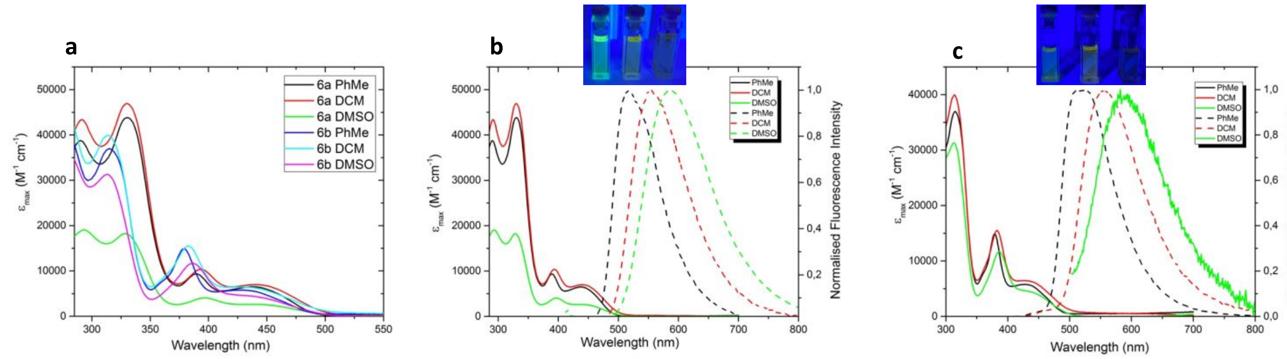

Figure S47. **a)** UV/vis spectra of **6a** and **6b**; **b)** Absorption (solid line) and normalized emission (dotted line) spectra of the pull-push chromophores (**6a**) (excitation at 400 nm); **c)** Absorption (solid line) and normalized emission (dotted line) spectra of the pull-push

chromophores (**6b**) (excitation at 400 nm) measured in PhMe (black), DCM (red) and DMSO (green).

6. References

(1) Tasior, M.; Vakuliuk, O.; Koga, D.; Koszarna, B.; Górski, K.; Grzybowski, M.; Kielesiński, Ł.; Krzeszewski, M.; Gryko, D.T. Method for the Large-Scale Synthesis of Multifunctional 1,4-Dihydro-pyrrolo[3,2-*b*]pyrroles. *J. Org. Chem.* **2020**, *85* (21), 13529-13543.

(2) Tasior, M.; Koszarna, B.; Young, D. C.; Bernard, B.; Jacquemin, D.; Gryko, D.; Gryko, D. T. Fe(III)-Catalyzed Synthesis of Pyrrolo[3,2-b]pyrroles: Formation of New Dyes and Photophysical Studies. *Org. Chem. Front.* **2019**, *6*, 2939-2948.

(3) Medici, A.; Pedrini, P.; Venturoli, C.; Dondoni. A. Functionalization of Thiazoles. Selectivity in the Reactions of 2-(Dimethylamino)-l,3-thiazoles with Electrophiles; Formation of Michael-Type Adducts and Thiazolium Salts. *J. Org. Chem*. **1981**, *46*, 2790 – 2793.

(4) Gaussian 16, Revision A.03, Frisch, M. J.; Trucks, G. W.; Schlegel, H. B.; Scuseria, G. E.; Robb, M. A.; Cheeseman, J. R.; Scalmani, G.; Barone, V.; Petersson, G. A.; Nakatsuji, H.; Li, X.; Caricato, M.; Marenich, A. V.; Bloino, J.; Janesko, B. G.; Gomperts, R.; Mennucci, B.; Hratchian, H. P.; Ortiz, J. V.; Izmaylov, A. F.; Sonnenberg, J. L.; Williams-Young, D.; Ding, F.; Lipparini, F.; Egidi, F.; Goings, J.; Peng, B.; Petrone, A.; Henderson, T.; Ranasinghe, D.; Zakrzewski, V. G.; Gao, J.; Rega, N.; Zheng, G.; Liang, W.; Hada, M.; Ehara, M.; Toyota, K.; Fukuda, R.; Hasegawa, J.; Ishida, M.; Nakajima, T.; Honda, Y.; Kitao, O.; Nakai, H.; Vreven, T.; Throssell, K.; Montgomery, J. A.; Peralta, Jr., J. E.;

Ogliaro, F.; Bearpark, M. J.; Heyd, J. J.; Brothers, E. N.; Kudin, K. N.; Staroverov, V. N.; Keith, T. A.; Kobayashi, R.; Normand, J.; Raghavachari, K.; Rendell, A. P.; Burant, J. C.; Iyengar, S. S.; Tomasi, J.; Cossi, M.; Millam, J. M.; Klene, M.; Adamo, C.; Cammi, R.; Ochterski, J. W.; Martin, R. L.; Morokuma, K.; Farkas, O.; Foresman, J. B.; Fox, D. J. Gaussian, Inc., Wallingford CT, 2016.

(5) Zhao, Y.; Truhlar, D. G. The M06 suite of density functionals for main group thermochemistry, thermochemical kinetics, noncovalent interactions, excited states, and transition elements: two new functionals and systematic testing of four M06-class functionals and 12 other functionals. *Theor. Chem. Acc.* **2008**, *120*, 215−241.

(6) Tomasi, J.; Mennucci, B.; Cammi, R. Quantum Mechanical Continuum Solvation Models. *Chem. Rev.* **2005**, *105*, 2999−3094.

(7) Guido, C. A.; Chrayteh, A.; Sclamani, G.; Mennucci, B.; Jacquemin D. Simple Protocol for Capturing Both Linear-Response and State-Specific Effects in Excited-State Calculations with Continuum Solvation Models. *J. Chem. Theory Comput.* **2021**, *17*, 5155−5164.

(8) (a) Laurent, A. D.; Jacquemin, D. TD-DFT benchmarks: A review. *Int. J. Quantum Chem.* **2013**, *113*, 2019−2039. (b) Christiansen, O.; Koch, H.; Jørgensen, P. The second-order approximate coupled cluster singles and doubles model CC2. *Chem. Phys. Lett.* **1995**, *243,* 409−418.

(9) TURBOMOLE V7.3/V7.5, *a development of University of Karlsruhe and Forschungszentrum Karlsruhe GmbH*, 1989−2007; TURBOMOLE GmbH. http://www.turbomole.com.

(10) Jacquemin, D.; Duchemin, I.; Blase, X. The second-order approximate coupled cluster singles and doubles model CC2. *J. Chem. Theory Comput.* **2015**, *11*, 5340−5359.

(11) Hellweg, A.; Grün, S.; Hättig, C. Benchmarking the performance of spin-component scaled CC2 in ground and electronically excited states. *Phys. Chem. Chem. Phys.* **2008**, *10,* 1159−1169.

(12) Pershin, A.; Hall, D.; Lemaur, V.; Sanchi-Garcia, J. C.; Muccioli, L.; Zysman-Colman, E.; Beljonne, D.; Olivier, Y. Highly emissive excitons with reduced exchange energy in thermally activated delayed fluorescent molecules. *Nat. Commun.* **2019**, 10, 597.

(13) Neese, F.; Wennmohs, F.; Becker, U.; Riplinger, C. The ORCA quantum chemistry program package. *J. Chem. Phys.* **2020**, 152, 224108.

(14) do Casal, M. T.; Veys, K.; Bousquet, M. H. E. ; Escudero, D.; Jacquemin, D. First-Principles Calculations of Excited-State Decay Rate Constants in Organic Fluorophores. *J. Phys. Chem. A.,* **2023**, 127, 10033−10053.

(15) Cerezo, J. ; Santoro, F. FCClasses 3.0, can be found under http://www.pi.iccom.cnr.it/fcclasses

(16) Cerezo, J.; Santoro, F. FCclasses3: Vibrationally-resolved spectra simulated at the edge of the harmonic approximation. *J. Comput. Chem*. **2023**, 44, 626−643.

(17) Santoro, F.; Jacquemin, D. Going beyond the vertical approximation with time-dependent density functional theory. *Wires Comput. Mol. Sci*. **2016**, 6, 460–486.

(18) Peng, Q.; Yi, Y.P.; Shuai, Z.G.; Shao, J.S. Excited state radiationless decay process with Duschinsky rotation effect: Formalism and implementation. *J. Chem. Phys*. **2007**, 126, 114302.

(19) Humemiuk, A.; Buzancic, M.; Hoche, J.; Cerezo, J.; Mitric, R.; Santoro, F.; Bonacic-Koutecy, V. Predicting fluorescence quantum yields for molecules in solution: A critical assessment of the harmonic approximation and the choice of the lineshape function. *J. Chem. Phys.* **2020**, 152, 054107.

(20) Ou, Q.; Peng, Q.; Suhai, Z. Toward Quantitative Prediction of Fluorescence Quantum Efficiency by Combining Direct Vibrational Conversion and Surface Crossing: BODIPYs as an Example. *J. Phys. Chem. Lett.,* **2020**, 11, 7790–7797.

(21) Rybczynski, P.; Bousquet, M. H. E.; Kaczmarek-Kedziera, A.; Jedrzejewska, B.; Jacquemin, D.; Osmiałowski, B. Controlling the fluorescence quantum yields of benzothiazole-difluoroborates by optimal substitution. *Chem. Sci.,* **2022**, 13, 13347–13360.

(22) Epifanovsky, E. et al. Software for the frontiers of quantum chemistry: An overview of developments in the Q-Chem 5 package. *J. Chem. Phys.*, **2021**, *155*, 084801.
